# Self-Assembled Multilayers Reduce Interfacial Energy Loss in Perovskite Solar Cells

**DOI:** 10.1038/s41467-026-76497-1

**Published:** 2026-08-08

**Authors:** Yi Pan, Lei Liu, Haoxuan Guo, Changqing Lin, Pengfei Wu, Zeping Ou, Can Wang, Peidong Chen, Qin Gao, Mingyang Gao, Xiaoxue Lin, Dingqin Hu, Tingming Jiang, Yujie Zheng, Zeyun Xiao, Ke Yang, Zeyu Zhang, Rui Wang, Nabonswende Aida Nadege Ouedraogo, Zedong Lin, Wei Wei, Kuan Sun, Qiang Liao

**Affiliations:** 1 MOE Key Laboratory of Low-grade Energy Utilization Technologies and Systems, School of Energy & Power Engineering, Chongqing University, Chongqing, China; 2 Department of Chemistry and Materials Engineering, Kansai University, Suita, Osaka, Japan; 3 Institute of Systems and Physical Biology, Shenzhen Bay Laboratory, Shenzhen, Guangdong China; 4 Wu Jieh Yee School of Interdisciplinary Studies, Lingnan University, Tuen Mun, Hong Kong China; 5 Department of Mechanical Engineering, City University of Hong Kong, Kowloon Tong, Hong Kong China; 6 Chongqing Institute of Green and Intelligent Technology, Chinese Academy of Sciences, Chongqing, China; 7 School of Physics and Optoelectronic Engineering, Hangzhou Institute for Advanced Study, University of Chinese Academy of Sciences, Hangzhou, China; 8 School of Engineering, Westlake University, Hangzhou, China; 9 School of Materials Science and Engineering, Taizhou University, Taizhou, China; 10 Key Laboratory of Optoelectronic Technology and System, Ministry of Education of China, College of Optoelectronic Engineering Chongqing University, Chongqing, China

**Keywords:** Solar cells, Devices for energy harvesting, Energy

## Abstract

The use of self-assembled multilayer (SAM) layers as hole transport layers (HTLs) represents a major advance for high-efficiency perovskite solar cells (PSCs). However, many SAMs materials suffer from aggregation, poor wettability, and weak interactions with the perovskite, which hinder charge transfer and cause energy losses that limit both power conversion efficiency (PCE) and long-term stability. In this study, we synthesized two SAMs, namely 2-(10-(3,5-dimethoxyphenyl)−7H-benzo[c]carbazol-7-yl)ethyl)phosphonic acid (denoted as DMPA) and 2-(7H-benzo[c]carbazol-7-yl)ethyl)phosphonic acid (denoted as BCPA). DMPA SAM effectively suppresses self‑aggregation and enhances substrate coverage. The methoxy groups in DMPA interact with the perovskite, thereby enabling DMPA to passivate defects at the buried interface and optimize perovskite crystallization. These interfacial improvements facilitate more efficient charge extraction and enhance interfacial stability. As a result, DMPA-based PSCs achieve a PCE of 27.59% (certified PCE of 27.2%) and show remarkable photothermal stability, retaining 94.5% of their initial efficiency after 1600 hours of continuous illumination under 1 Sun at 65 °C.

## Abstract (web summary)

Self-assembled interlayers improve perovskite solar cells but suffer aggregation, incomplete coverage, and weak interfaces, limiting efficiency and stability. Pan et al. design a methoxy-functionalized molecule improving wettability and interface contact, boosting efficiency and long-term stability.

## Introduction

Photovoltaics, which convert light into clean electricity without greenhouse gas emissions, are becoming increasingly crucial for supporting society’s shift to net-zero carbon dioxide-equivalent emissions^1^. Typical metal halide perovskite solar cells (PSCs) comprise five layers including a transparent conductive layer, an n-type electron-collecting layer, a light-absorbing perovskite layer, a p-type hole-collecting layer, and a metal electrode. These devices are categorized as regular (n-i-p) or inverted (p-i-n) structures, based on whether electrons or holes are extracted to the transparent bottom electrode^2,3^. Inverted PSCs exhibit superior durability, compatibility with tandem configurations, and market-integration potential compared to their conventional counterparts. Moreover, inverted PSCs have achieved notable advancements, with certified power conversion efficiency (PCE) surpassing 27%^4^. A key development in inverted device technology was the introduction of self-assembled multilayer (SAM) as a hole transport layer (HTL)^5^. SAMs generally consist of three components: (1) an anchoring group that facilitates chemical bonding to the substrate (phosphonic acid groups are favored for their strong affinity to metal oxides); (2) a terminal group that interacts with the overlying layer and can generate a dipole moment to modify energy levels; and (3) a linker that connects the anchoring and terminal groups, with its length and structure (conjugated or nonconjugated) influencing packing density and electronic properties^6^. The attractive features of SAMs, including well-defined structure, minimal parasitic absorption, structural tunability, adjustable energy levels, low synthesis cost, and the ability to passivate defects at the perovskite-HTL interface, have driven substantial performance gains through tailored SAM designs.

Although SAM-based PSCs have demonstrated impressive PCEs, several challenges remain. Common problems include incomplete SAM coverage, molecular agglomeration, and poor wettability caused by the mismatch between nonpolar SAM head groups and polar perovskite precursor solutions^7^. A range of strategies has been developed to address these issues^8^. For instance, increasing SAM concentration or using mixtures of SAM can reduce vacancy defects, but this may also lead to aggregation. Importantly, only a dense and uniform SAM can reliably function as an effective HTL, offering optimized energy levels for charge extraction^9^. Post‑deposition treatments such as solvent rinsing remove excess molecules and diminish aggregation, while the addition of small molecules that promote π–π stacking at the interface can lower heterojunction energy losses^10^. However, these approaches often increase fabrication complexity. Additionally, the interactions between the planar π-conjugated SAM cores and the perovskite, typically involving electrostatic or van der Waals forces, are susceptible to UV-induced disruption, which can impair hole extraction and undermine long-term stability^11^.

Chemical engineering strategies have therefore been applied to modify the anchoring group, terminal group, or linker of SAM, thereby controlling SAM orientation, packing density, wettability, and energy alignment between the HTL and perovskite. Recent studies show that tuning the terminal group, which interfaces with the perovskite (PVK) layer, is particularly effective for adjusting energy levels and molecular packing. For instance, introducing extended π-systems or methoxy substituents has been shown to enhance intermolecular interactions and perovskite anchoring. For example, Du et al. designed (4-(9’-phenyl-9H,9’H-[3,3’-bicarbazol]−9-yl) butyl) phosphonic acid (4PABCz) with a specific π-extended structure that induces an out-of-plane dipole^12^. This dipole prompts the molecules to adopt a parallel, face-on orientation relative to the substrate, producing a densely packed layer that functions as an effective HTL with improved charge extraction and reduced interfacial recombination compared with related analogs. Similarly, Qu et al. reported a self-assembled hole-selective layer comprising 4-(7H-dibenzo[c,g]carbazol-7-yl)phenyl)phosphonic acid (Bz-PhpPACz) featuring π-expanded conjugation^13^. Bz‑PhpPACz molecules align parallel through π–π stacking and form a layered structure along the b axis, with phosphate groups alternating up and down in each repeat unit. This enhanced intermolecular π–π interaction facilitates the formation of an ordered bilayer with a hydrophilic surface, passivating defects at the buried perovskite interface and supporting high-quality, large-area perovskite film preparation, while enhancing charge extraction and transport. Meanwhile, Wu et al. incorporated methoxy groups on the benzene ring at various positions (ortho, meta, and para)^11^. These functional groups enhance anchoring to the perovskite and fine-tune the molecular dipole moment. Consequently, M-PhPACz demonstrates optimal properties, with a maximum dipole moment and an O − O distance that aligns precisely with the diagonal lead ions in the adjacent perovskite lattice, thereby improving SAM-perovskite interactions, charge extraction efficiency, and interfacial stability. Despite these advances, a key challenge remains: designing a SAM molecule whose terminal group is systematically engineered to simultaneously promote dense, uniform molecular packing and establish robust, defect-passivating chemical bonds with the perovskite, thereby delivering both high efficiency and long‑term stability.

**Fig. 1:**
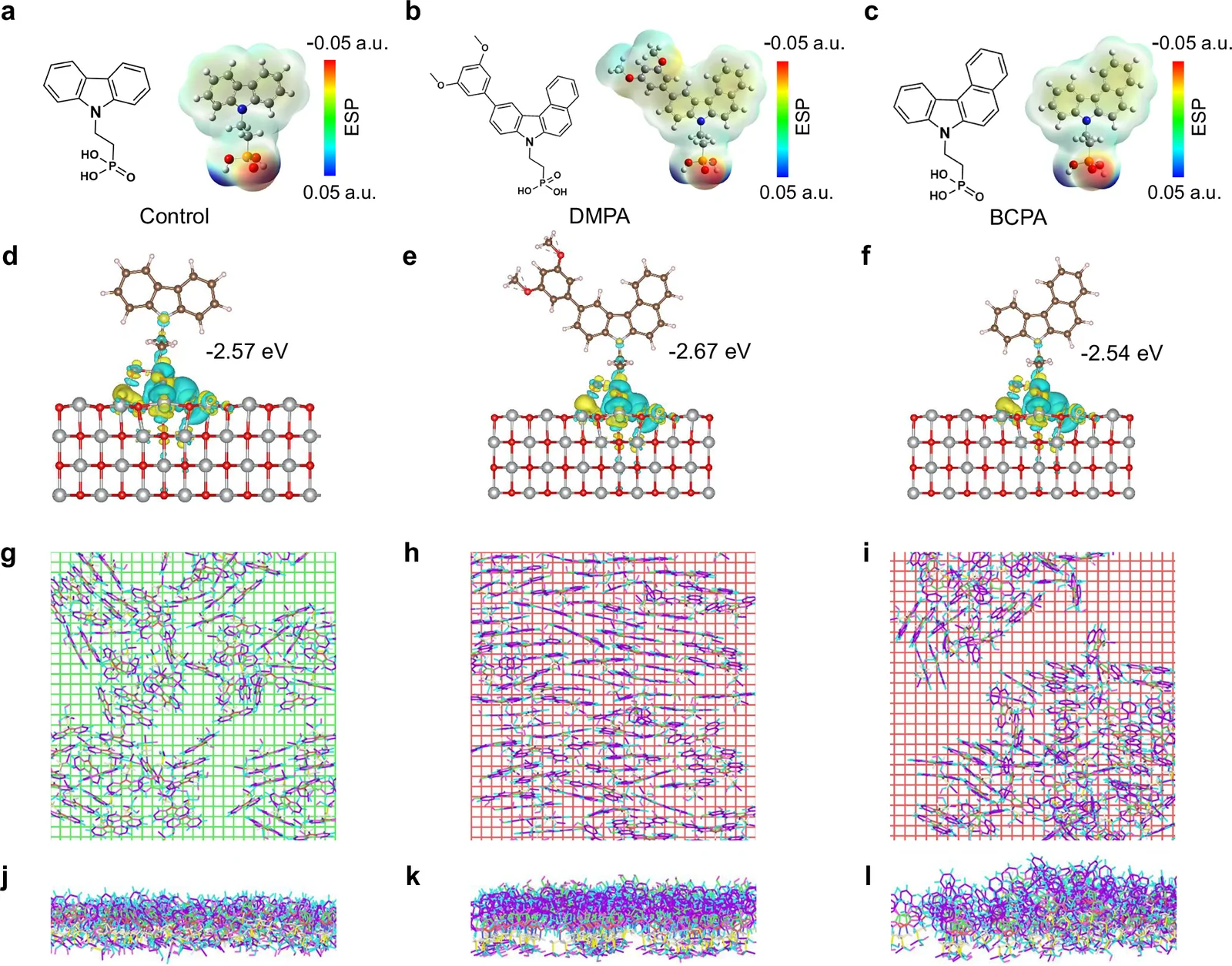
Computational analysis of self‑assembled multilayers. **a**–**c** Chemical structures and electrostatic potential surfaces (EPSs) of the control, DMPA, and BCPA molecules. **d**–**f** Electron density difference plots for the three SAMs adsorbed on the NiO_x_ substrate. **g**–**i** Top-view and **j**–**l** side-view molecular dynamics snapshots of the SAM configurations on NiO_x_, illustrating differences in packing order, molecular orientation, and surface coverage.

**Fig. 2:**
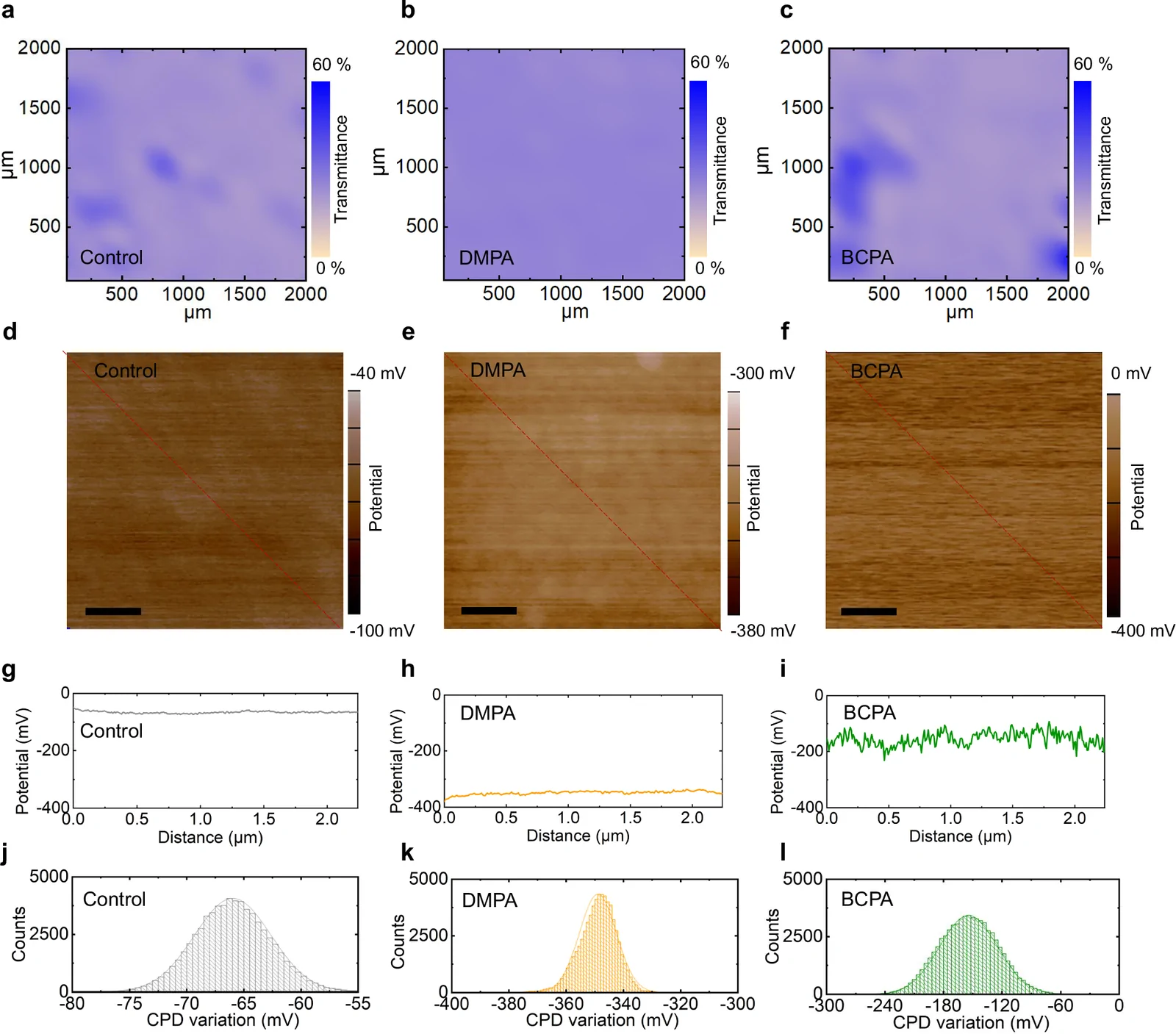
The interaction between SAM and NiO_x_. FT-IR mapping of **a** NiO_x_/ Control SAM at 1040 cm^−1^, **b** NiO_x_/DMPA at 1042 cm^−1^, and **c** NiO_x_/BCPA at 1042 cm^−1^. KPFM surface potential images of **d** NiO_x_/ Control SAM, **e** NiO_x_/DMPA and **f** NiO_x_/BCPA (scale bar of 1 µm). The CPD line scan extracted from the dotted line position for **g** NiO_x_/ Control SAM, **h** NiO_x_/DMPA, and **i** NiO_x_/BCPA. The CPD distribution histogram of **j** NiO_x_/ Control SAM, **k** NiO_x_/DMPA and **l** NiO_x_/BCPA.

**Fig. 3:**
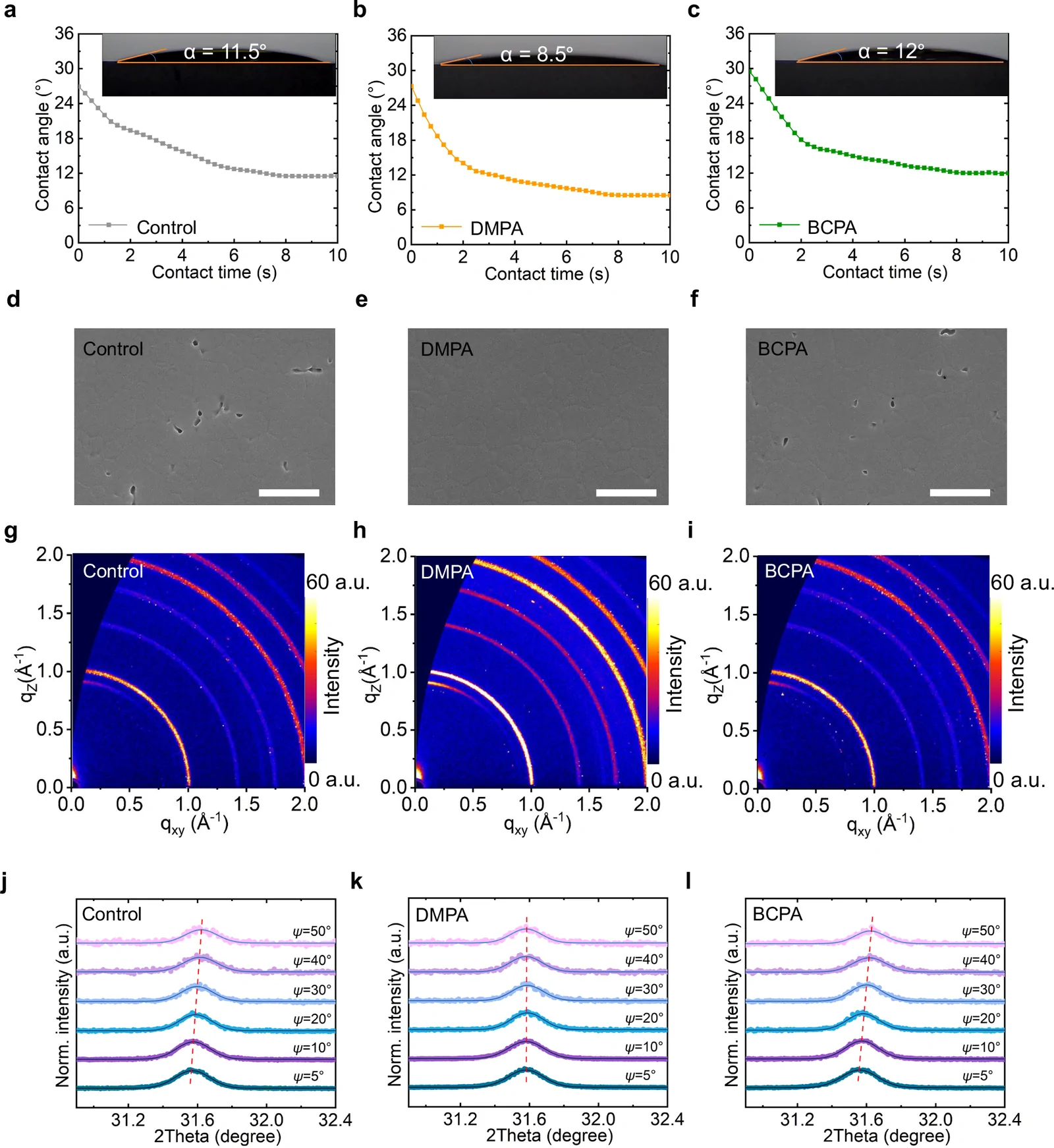
Crystallization study of perovskite films. **a**–**c** Contact angle of the perovskite precursor solution on different SAMs varying with time; insert final 10 s contact angle image. **d**–**f** SEM images of the bottom surface of perovskite deposited on different SAMs (scale bar of 2 µm). **g**–**i** GIWAXS patterns of the perovskite films deposited on different SAMs. **j**–**l** GIXRD patterns measured at different *ψ* values ranging from 5° to 50° for perovskite films on different SAMs.

**Fig. 4:**
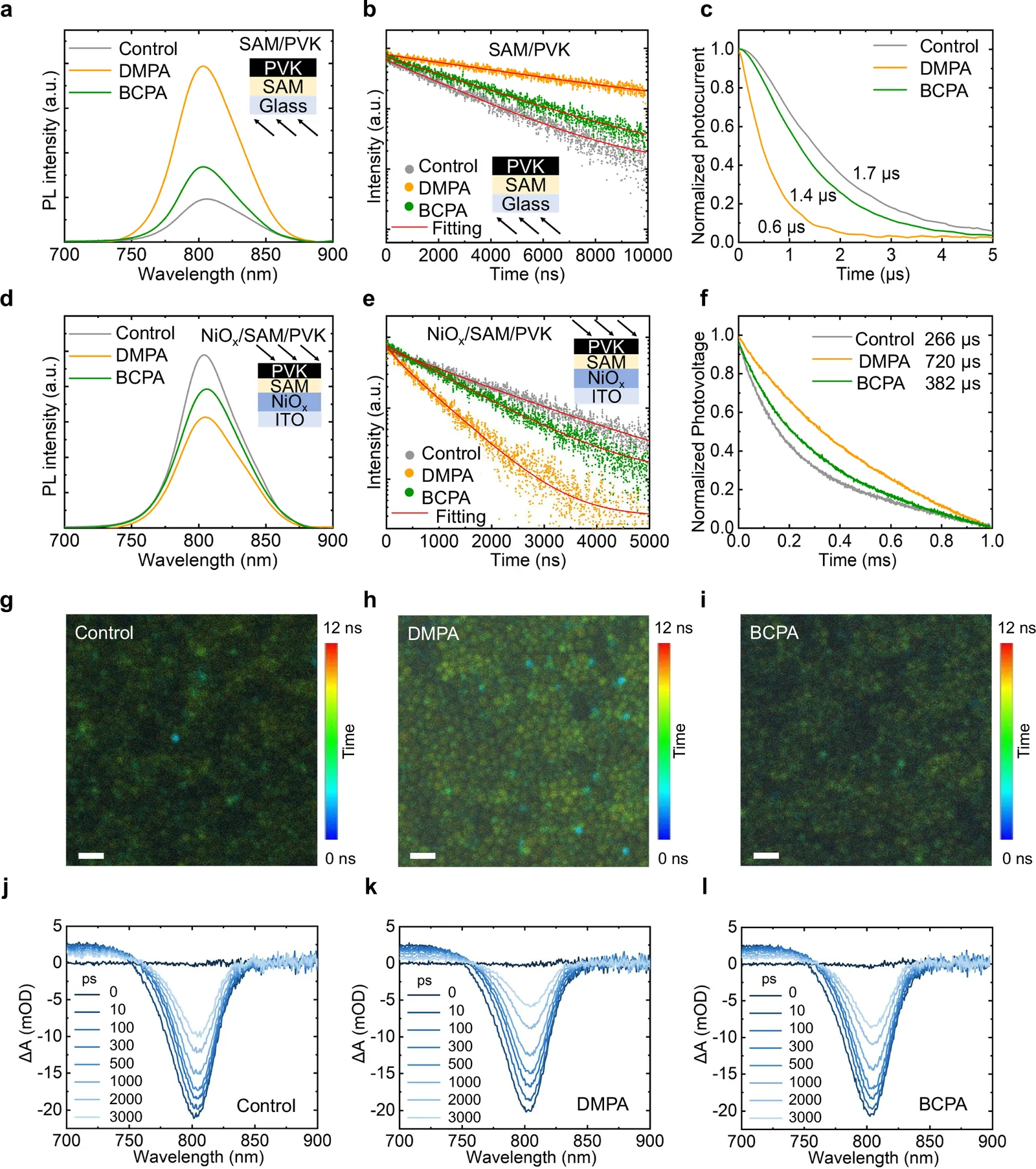
Carrier transport and recombination dynamics. **a** Steady-state PL spectra and **b** TRPL spectra of perovskite films (Shine the film from one side of the glass). **c** TPC curves-based devices. **d** Steady-state PL spectra and **e** TRPL spectra of perovskite films (Shine the film from one side of the PVK film). **f** TPV curves-based devices. **g-i** PL lifetime mapping images of perovskite films (scale bar of 2 µm). Femtosecond transient absorption spectra at a selected pump–probe delay time of **j**, NiO_x_/control/perovskite films, **k** NiO_x_/DMPA/perovskite films, and **l**, NiO_x_/BCPA/perovskite films. ΔA, difference in optical density between the excited state and the ground state of a sample.

**Fig. 5:**
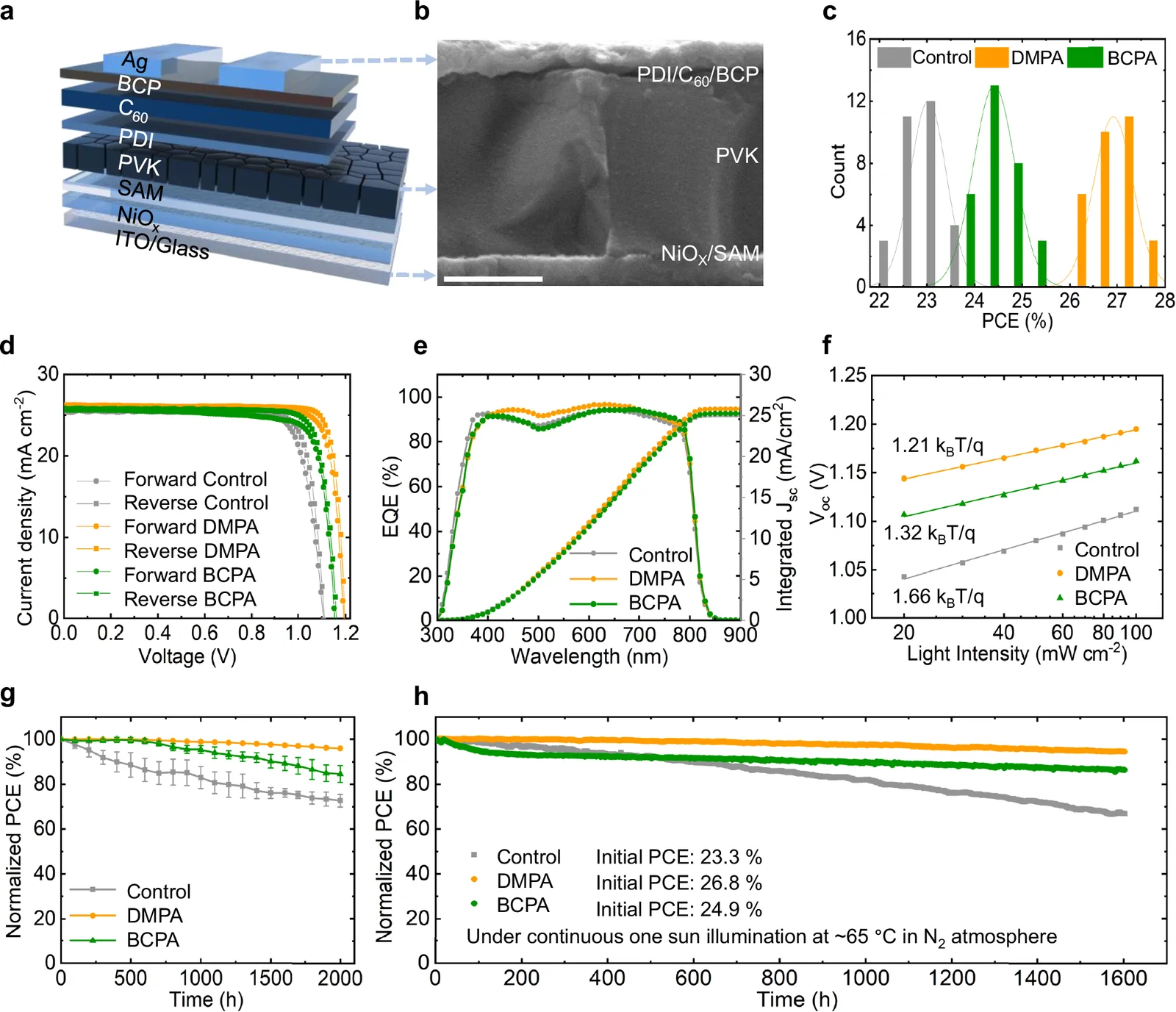
Configuration and photovoltaic performance of PSCs. **a** Device structure of PSC. **b** Cross-sectional SEM image of the DMPA-based device (scale bar of 500 nm). **c** The statistics of the PCE values for the PSCs. **d** *J*-*V* curves of different devices. **e** EQE spectrum of the devices with an integrated *J*_*sc*_. **f** Light intensity dependence on *V*_OC_ plots from devices. **g** Stability of unencapsulated devices in N_2_ atmosphere (For each type of device, the data points represent average values obtained from 3 devices, while the error bars indicate the maximum and minimum values at each point). **h** MPP tracking under N_2_ and simulated 1 sun AM 1.5 G illumination for devices (reaching an operating temperature of 65 ± 10 °C).

Here, we hypothesize that integrating a methoxy-functionalized π-extended aromatic terminal group, would synergistically enhance intermolecular order and promote direct chemical interaction with the perovskite. To test this, building on the popular (2-(9H-carbazol-9-yl)ethyl) phosphonic acid (denoted as control, Fig. 1a), we designed and synthesized two SAMs materials: 2-(10-(3,5-dimethoxyphenyl)−7H-benzo[c]carbazol-7-yl)ethyl) phosphonic acid (denoted as DMPA, Figs. 1b) and 2-(7H-benzo[c]carbazol-7-yl)ethyl) phosphonic acid (denoted as BCPA, Fig. 1c). Results demonstrated that DMPA SAM effectively suppresses their own aggregation and enhance substrate coverage. Moreover, the methoxy groups in DMPA were proven to interact with the perovskite, thereby effectively passivating defects at the buried interface and thus optimizing perovskite crystallization. These combined effects not only improved charge extraction and transport, but also enhanced interfacial stability. Consequently, PSCs incorporating DMPA achieved a PCE of 27.59 % (Certified PCE is 27.2 %) and excellent photothermal stability, retaining 94.5% of their initial efficiency after 1600 hours of continuous illumination under 1 Sun at 65 °C. This work demonstrates that terminal-group engineering is a promising strategy for creating multifunctional SAM that meet the efficiency and stability requirements for advancing perovskite photovoltaics.

## Results

### Synthesis and structural characterization of SAMs

The synthetic routes of DMPA and BCPA are described in the supporting information, where their chemical structures were confirmed by nuclear magnetic resonance (1H NMR and ¹³C NMR), and high-resolution mass spectrometry (HRMS) spectra as shown in Supplementary Figs. 1–6.

### Theoretical simulation of SAMs

We first employed density functional theory (DFT) to investigate the electrostatic potential (ESP) of the three molecules (Fig. 1a–c). The results show that the conjugated carbazole moiety is electron‑rich, endowing the molecules with excellent hole‑transport properties. The phosphonic acid headgroups also contain electron‑rich oxygen atoms, supporting strong binding to NiO_x_ and confirming phosphonic acids as effective anchors for this inorganic HTL. Subsequently, DFT was applied to compute the adsorption energy (*E*_*ad*_) of each SAMs on NiO_x_ (Fig. 1d–f). Among the three SAMs, DMPA exhibits the most negative *E*_*ad*_ (−2.67 eV), followed by the control SAM (−2.57 eV) and BCPA (−2.54 eV), indicating the highest affinity of DMPA for the NiO_x_ surface.

Molecular dynamics (MD) simulations visualized the top and side views of Control, BCPA, and DMPA on the NiO_x_ substrate (Fig. 1g–l). The results show that DMPA SAM effectively suppresses its own aggregation and improves substrate coverage. This can be attributed to the optimal π-π interactions in DMPA, which enhance surface coverage, while the rotating phenyl ring introduces steric hindrance that effectively inhibits molecular stacking. In contrast, BCPA displays a thickness of approximately 1.6 nm, likely due to its greater tendency for molecular aggregation (Supplementary Fig. 7).

Additionally, we utilized DFT to determine *E*_*ad*_ of these SAMs on PVK. Notably, compared to *E*_*ad*_ of control (−0.28 eV) and BCPA (−0.32 eV), the *E*_*ad*_ of DMPA (−0.39 eV) with perovskite is the most negative (Supplementary Fig. 8a), signifying that DMPA possesses the strongest affinity among the three SAMs. Additionally, DFT indicates that the methoxy oxygens in DMPA can chemically interact with undercoordinated Pb^2+^ at the perovskite interface. Dual O···Pb bonds strongly chelate antisite Pb^2+^ ions, reducing local octahedral distortion and increasing the defect formation energy from 1.31 eV (control) and 1.22 eV (BCPA) to 2.05 eV (DMPA) (Supplementary Fig. 8b). The interaction of O···Pb bonds is expected to lower interfacial defect densities and reduce transport losses, thereby improving SAM/perovskite interfacial performance.

### Interaction between SAMs and NiO_x_ substrate

We used Fourier‑transform infrared (FT‑IR) spectroscopy to identify molecular vibrational modes and confirm successful anchoring of the synthesized SAMs to NiO_x_^14,15^. The spectra show characteristic stretching vibrations of the phosphonic acid anchors, with bands near 954 cm^−1^ and 1036 cm^−1^ attributable to P-OH functional groups, and a band near 1068 cm^−1^ assigned to P = O (Supplementary Fig. 9a–c). Upon adsorption onto NiO_x_, these vibrational peaks shift for all SAMs (control, DMPA and BCPA), directly evidencing the chemical interaction between the phosphonic-acid anchoring groups and the NiO_x_ surface.

FT-IR mapping was also performed to evaluate the spatial homogeneity of the SAMs. The mapping was based on the absorption band at approximately 1042 cm⁻¹, which corresponds to the stretching vibration of the P–OH group^14,15^. The map of DMPA shows a uniform, continuous coverage across the NiO_x_ substrate, a marked improvement over the heterogeneous distribution observed for the control and BCPA SAM (Fig. 2a-c). The more uniform DMPA coverage is consistent with its stronger substrate affinity and more ordered assembly, which is expected to facilitate more efficient interfacial charge transfer and to promote uniform perovskite crystallization.

Subsequently, Kelvin probe force microscopy (KPFM) was employed to evaluate the surface potential distribution of the NiO_x_/SAM interface (Fig. 2d–f). The average surface contact potential difference (CPD) for NiO_x_/DMPA was substantially lower (−349.0 mV), as compared to −65.9 mV for the Control and −154.3 mV for BCPA (Fig. 2j–l). To quantify this energy-level shift, we performed ultraviolet photoelectron spectroscopy (UPS). The UPS results corroborate the KPFM findings, revealing a work function (WF) of −5.06 eV for NiO_x_/DMPA, distinctly shallower than the −4.97 eV, and −5.0 eV values for the Control and BCPA, respectively (Supplementary Fig. 10a). The corresponding HOMO levels were determined as −5.59 eV for DMPA, −5.35 eV for the Control, and −5.44 eV for BCPA (Supplementary Fig. 10b). The UPS and KPFM results indicate that the energy levels of DMPA align well with PVK^16,17^. The tailored energy level alignment imparted by DMPA reduces the hole-transfer barrier at the HTL/PVK interface, thereby facilitating charge extraction and contributing to an increased open-circuit voltage (*V*_OC_)^18^.

To assess how the SAM affect charge transport through the NiO_x_/SAM layer, we measured conductivity and hole mobility using devices with the structure of ITO/NiO_x_/SAM (Control, DMPA and BCPA)/Ag (Supplementary Fig. 11). The conductivity (σ) was calculated using the following formula (1)^19^:

1 $${{{\rm{\sigma }}}}=\frac{d}{{AR}}$$

where *R*, *d*, and *A* represent the resistance, thickness of the HTL, and the thin film area, respectively. The average conductivity (σ) values based on five samples of NiO_x_/Control, NiO_x_/DMPA, and NiO_x_/BCPA were 9.6 × 10^−5^ S cm^−1^, 1.5 × 10^−4^ S cm^−1^, and 1.1 × 10^−4^ S cm^−1^, respectively. NiO_x_/DMPA exhibits the highest conductivity, consistent with the improved energy-level alignment shown above. This enhanced conductivity indicates that DMPA better facilitates hole extraction and transport across the HTL compared with the control and BCPA.

Hole mobilities were extracted from the current density–voltage (*J*–*V*) curves of the HTL-only devices by fitting to the Mott–Gurney law^19^. Among the samples, NiO_x_/DMPA exhibited the highest hole mobility (2.8 × 10^−4^ cm^2^ V^−1^ S^−1^), surpassing those of NiO_x_/Control SAM (1.6 × 10^−4^ cm^2^ V^−1^ S^−1^) and NiO_x_/BCPA (1.7 × 10^−4^ cm^2^ V^−1^ S^−1^) (Supplementary Fig. 12). This enhanced mobility facilitates more efficient hole transfer and mitigates charge accumulation at the HTL/perovskite interface.

Subsequently, we quantified the surface density of SAMs on the ITO substrate by cyclic voltammetry (CV)^2,20,21^. The surface molecular density of NiO_x_/DMPA (6.8 × 10^13^ molecules cm^−^^2^) was higher than that of NiO_x_/Control SAM (4.5 × 10^13^ molecules cm^−^^2^) and NiO_x_/BCPA (5.0 × 10^13^ molecules cm^−^^2^) (Supplementary Fig. 13a–f). These findings confirm that DMPA yields a more homogeneous, higher-coverage HTL, consistent with the FT-IR mapping data.

### Crystallization of perovskite films

The quality of the PVK film is critically influenced by the underlying substrate. To investigate the impact of SAM on perovskite crystallization, we first analyzed the temporal changes in contact angle to evaluate wetting properties across various surfaces (Fig. 3a–c). The perovskite precursor on NiO_x_/DMPA substrates exhibited a low contact angle of 8.5°, which was lower and associated with a shorter spreading time compared to NiO_x_/Control and NiO_x_/BCPA substrates. This suggests that the DMPA layer improves wetting, thereby promoting perovskite crystal growth.

Additionally, we inspected the morphologies of the buried interface between the perovskite layer and the charge transport layer by detaching the perovskite film from the substrate (Fig. 3d–f). Perovskite films on the control substrate showed evident defects, such as pinholes, which are likely attributable to SAM-reduced wettability during perovskite film formation. Conversely, films deposited on DMPA were markedly denser and more uniform, with no detectable pinholes, consistent with improved carrier transport across the interface. Tensile testing revealed that the DMPA-treated interface possessed higher tensile strength (51.5 N cm^−2^) than the Control (37.3 N cm^−^^2^) or BCPA (33.0 N cm^−2^) SAM, suggesting stronger adhesion and tighter molecular contact at the SAM/perovskite interface (Supplementary Fig. 14). These observations demonstrate that DMPA produces a more cohesive, defect-suppressed buried interface, favoring efficient charge extraction and mechanical robustness.

We then performed X-ray diffraction (XRD) on perovskite films deposited on the different SAMs prior to device assembly^22,23^. The PVK film grown on the DMPA-treated NiO_x_ exhibits a sharper and more intense diffraction peak for the (100) plane, with a reduced full width at half maximum (FWHM = 0.10°) compared with control and BCPA films (FWHM = 0.11°) (Supplementary Fig. 15), indicating improved bulk crystallinity. We attribute this enhancement to the uniform DMPA SAM distribution and its favorable wetting properties, which promote more homogeneous nucleation and crystal growth for PVK.

To gain a deeper, spatially resolved understanding of this crystallographic advantage, we employed grazing incidence wide-angle X-ray scattering (GIWAXS). The GIWAXS patterns (Fig. 3g–i), unequivocally show that DMPA-treated perovskite films possess brighter and more homogeneous diffraction rings for the (100) plane (*q* ≈ 1.0 Å⁻¹), in contrast to the film on Control or BCPA SAM, suggesting that DMPA-treated PVK films have higher crystalline quality^24^.

To uncover the underlying mechanism for crystal growth, we further conducted in-situ GIWAXS measurement under the programmed spin-coat and annealing procedure. The corresponding false-color maps of the control, DMPA, and BCPA of the diffraction intensity versus scattering vector *q* (in units per angstrom) and time (in seconds) are shown in Supplementary Fig. 16a–c. At the moment of antisolvent dripping, a solvent phase emerged. Subsequently, during the annealing process, characteristic diffraction peaks were observed at *q* = 1.00, 1.42, and 1.73 Å^−1^, corresponding to the (100), (110), and (111) facets of the perovskite, respectively. Compared to the Control and BCPA, DMPA exhibits a prolonged duration of the solvent phase, suggesting that the SAM molecule modulates perovskite growth. In other words, DMPA retards the crystallization kinetics of the perovskite, which can enhance perovskite crystallization and growth.

To probe the influence of SAM on longitudinal stress distribution in perovskite films, we performed depth-dependent grazing incidence X-ray diffraction (GIXRD) measurements (Fig. 3j–l). For the control or BCPA-based perovskite (PVK) films, increasing the incidence angle ψ from 5° to 50° caused obvious shifts in the (012) diffraction peaks toward larger angles, indicating a contracted lattice with residual compressive stress at the bottom interface. In contrast, the DMPA PVK film showed no noticeable shift in the (012) diffraction peak, suggesting negligible compressive stress.

To quantitatively estimate the residual stress, values were calculated using this following Eq. (2) ^25,26^:

2 $${{{\rm{\sigma }}}}=-\frac{{E}_{p}}{\left(1+{\nu }_{p}\right)}\frac{\pi }{{180}^{\circ }}\cot {\theta }_{0}\frac{\partial \left(2{\theta }_{0}\right)}{\partial \left[{(\sin \varPsi )}^{2}\right]}$$

ere *E*_*p*_ is Young’s modulus (10.2 GPa), *ν*_*p*_ is Poisson’s ratio (0.3), and *θ*_*0*_ is half the scattering angle for the stress-free (012) diffraction peak. Residual stress in perovskite films was determined by fitting 2*θ*_*0*_ as a function of sin²*ψ*; the slope indicates the strain, with negative values signifying tensile stress and positive values compressive stress^27^. The residual stresses for the control, DMPA, and BCPA-based PVK films were estimated to be 12.2, 0.8, and 14.7 MPa, respectively. The substantially reduced compressive strain in the DMPA-based film correlates with improved crystal quality and a more relaxed lattice, which helps explain the enhanced device performance and mechanical stability observed for DMPA‑based PSCs.

### Carrier transport and recombination dynamics

To examine charge transfer and recombination dynamics at the buried interface, ITO or glass/SAM/perovskite stacks were fabricated and characterized using steady-state photoluminescence (PL) and time-resolved photoluminescence (TRPL) spectroscopies. The glass/DMPA/perovskite stack displayed the highest PL intensity (Fig. 4a), indicating suppression of non-radiative recombination. TRPL measurements (Fig. 4b) yield an average PL lifetime τ_ave_ of 6.4 µs for DMPA, substantially longer than for the Control (2.3 µs) and BCPA (3.0 µs) samples (Fig. 4b), confirming reduced carrier trapping and slower non‑radiative decay. PL lifetime maps (Fig. 4g–i) show that DMPA‑templated films have both higher and more spatially uniform lifetimes, consistent with improved interfacial quality. This superior performance is attributed to the unique molecular structure of DMPA, which improves interfacial wettability and reduces pinhole formation, thereby effectively passivating the perovskite layer interfacial defects and minimizing recombination losses^17,28^.

However, the ultimate metric for a charge-transport layer is not only its passivation quality, but also its ability to selectively extract charges. To assess this, we studied the ITO/NiO_x_/SAM/perovskite stacks. The ITO/NiO_x_/DMPA/perovskite stack exhibited the lowest PL intensity and shortest carrier lifetime (625 ns) relative to the Control (1.5 µs) and BCPA (1.1 µs) (Fig. 4d and 4e). The reduced PL and accelerated decay indicate that DMPA does more than passivate the buried interface; it facilitates rapid hole extraction from the perovskite into the NiO_x_ layer^29,30^.

Space-charge-limited-current (SCLC) measurements were used to assess charge defect density in hole-only devices (ITO/NiO_x_/SAM/PVK/Spiro-OMeTAD/MoO_x_/Ag), with trap density (n_trap_) calculated using the standard formula (3)^31–33^:

3 $${n}_{{trap}}=\frac{2{\varepsilon }_{r}{\varepsilon }_{0}{V}_{{TFL}}}{q{L}^{2}}$$

where *V*_TFL_ is the trap-filled limit voltage, *ε*_r_ is the relative dielectric constant, *ε*_0_ is the vacuum permittivity, and *L* is the perovskite film thickness. Extracted values (Supplementary Fig. 17a–c) show that the DMPA‑modified device has a substantially lower hole trap-state density (2.9 × 10^15 ^cm^−3^) than the Control (6.0 × 10^15 ^cm^−3^) and BCPA (3.6 × 10^15 ^cm^−3^) devices. This reduction in n_trap_ corroborates the PL and TRPL findings, confirming that DMPA effectively reduces interfacial defect sites, thereby lowering carrier capture and non‑radiative losses. We ascribe the improvement to the combination of enhanced bottom‑interface morphology and chemical passivation provided by the DMPA SAM, which together produce a cleaner, more defect‑free perovskite/NiO_x_ interface and facilitate more efficient hole transport.

Transient photovoltage (TPV) and transient photocurrent (TPC) spectroscopy were utilized to further analyze charge carrier lifetime and extraction properties (Fig. 4c, 4f). The charge transport time for the DMPA device (0.6 µs) is shorter than that for the control (1.7 µs) and BCPA (1.4 µs) devices, confirming superior charge transport efficiency. At the same time, TPV reveals substantially longer carrier lifetimes under open‑circuit perturbation: the DMPA device exhibits a longer carrier recombination time (720 µs) compared to the control (266 µs) and BCPA (382 µs) devices. The simultaneous observation of faster extraction and extended recombination lifetimes implies that DMPA accelerates charge removal from the perovskite, which reduces the probability of interface recombination. It also lowers the density of recombination centers that would otherwise shorten lifetimes^34^.

To quantify the non-radiative recombination losses at the HTL/perovskite interface, photoluminescence quantum yield (PLQY) measurements were executed to quantify quasi-Fermi level splitting (QFLS) (Supplementary Fig. 18). The DMPA sample showed the highest QFLS value of 1.20 eV than control (1.12 eV) and BCPA (1.17 eV), which aligns well with the corresponding open-circuit voltage (*V*_*OC*_) trend. Due to the reduction of morphological defects such as pinholes at the interface in the DMPA group, DMPA more effectively suppresses non-radiative recombination compared to the control and BCPA. The QFLS measurements also corroborate this result.

We further investigated perovskite film formation on various SAMs using UV-vis absorption spectroscopy and corresponding Tauc analysis (Supplementary Fig. 19a, b). The PVK film grown on DMPA retained a higher absorption intensity across the visible range than films on the control and BCPA substrates. Tauc plots also indicated a slightly reduced bandgap (*E*_*g*_) for the DMPA-based PVK film, which is advantageous for generating higher photocurrents^35^.

We further employed femtosecond transient absorption (TA) spectroscopy to investigate the interfacial charge dynamics in NiOₓ/SAM/perovskite stacks (Fig. 4j–l). All samples exhibit a characteristic ground-state bleaching (GSB) signal centered at ~803 nm, corresponding to state filling of the perovskite absorber. It should be noted that the GSB signal primarily reflects the population of excited states and does not directly represent free-carrier dynamics such as recombination or transport. In device-relevant structures, the recovery of the GSB signal is governed by a combination of carrier recombination and interfacial charge extraction processes.

The DMPA sample exhibits a faster GSB recovery compared to the control and BCPA samples (Supplementary Fig. 20). This behavior suggests more efficient interfacial hole extraction at the NiOₓ/SAM/perovskite interface in the presence of DMPA

### Photovoltaic performance of PSCs

We configured and fabricated inverted PSCs with the structure of ITO/NiO_x_/SAM/perovskite/PDI/C_60_/BCP/Ag (Figs. 5a, 5b). To elucidate the underlying physical mechanisms of photovoltaic performance enhancement, electrochemical impedance spectroscopy (EIS) measurements were conducted to investigate interfacial charge transport (Supplementary Fig. 21). The DMPA-based device displayed higher recombination resistance (*R*_*rec*_) compared to the control and BCPA devices, indicating optimized interface contact and suppressed charge recombination^36–38^.

Current density-voltage (*J-V*) curves were measured under AM 1.5 G illumination to evaluate PSC performance. Statistical analysis of 30 devices revealed that the DMPA-based devices achieve the highest average PCE (Fig. 5c). For the champion devices, *J*-*V* parameters under reverse and forward scans are summarized as follows: for the Control device (Fig. 5d), *V*_*OC*_ = 1.112 V and 1.111 V, *J*_*SC*_ = 25.6 mA cm^−2^ and 25.56 mA cm^−2^, *FF* = 82.64% and 80.71%, PCE = 23.53% and 22.92%, respectively; for the DMPA device, *V*_*OC*_ = 1.195 V and 1.193 V, *J*_*SC*_ = 26.58 mA cm^−2^ and 26.53 mA cm^−2^, *FF* = 86.89% and 84.86%, PCE = 27.59% and 26.86%, respectively; for the BCPA device, *V*_*OC*_ = 1.162 V and 1.159 V, *J*_*SC*_ = 25.85 mA cm^−2^ and 25.68 mA cm^−2^, *FF* = 83.66% and 81.25%, PCE = 25.13% and 24.19%, respectively. Moreover, the DMPA and control PSCs showed lower hysteresis indices of 2.6% versus 3.7% for the BCPA PSC, where the index is defined as (PCE_reverse_ − PCE_forward_)/PCE_reverse_. Importantly, an independent third‑party test (National Institute of Measurement and Testing Technology, NIMTT) certified the DMPA device with 27.2% PCE. External quantum efficiency (EQE) measurements demonstrated that the integrated *J*_*SC*_ values (Control: 25.09 mA cm^−2^, DMPA: 25.8 mA cm^−2^, BCPA: 25.26 mA cm^−2^) exhibited negligible deviations (less than 2.9%) from the *J-V* curves (Fig. 5e).

To assess trap-assisted nonradiative recombination in device performance, the light intensity dependence of current-voltage (*J-V*) characteristics was analyzed. Specifically, the *V*_*OC*_ versus logarithm of light intensity was examined (Fig. 5f). The DMPA-treated device showed a slope of 1.21 *k*_*B*_T/q, compared to 1.66 *k*_*B*_T/q for the control and 1.32 *k*_*B*_T/q for the BCPA-based device. This result demonstrates effective suppression of nonradiative recombination at the buried interface^22^.

The long-term stability of unencapsulated PSCs was also assessed under nitrogen (N_2_) conditions. After 2,000 hours of storage, the DMPA devices retained an average of 95.9% of their initial efficiency, whereas the control and BCPA devices retained 72.7% and 84.6%, respectively (Fig. 5g). Additionally, the operational stability of encapsulated PSCs was examined under continuous one sun equivalent white-light LED illumination at 65 °C in an N_2_ atmosphere with maximum power point tracking (MPPT). The DMPA device retained 94.5% of its initial efficiency after 1600 hours, surpassing the Control (66.9%) and BCPA (86.4%) devices (Fig. 5h). This improved stability in DMPA PSCs is attributed to a higher-quality buried interface, reduced pinholes, and effective passivation, thereby enhancing overall performance and reliability.

## Discussion

In this study, we demonstrate that targeted molecular engineering of SAM provides an effective route to simultaneously address the charge transport, interfacial recombination, and operational stability issues in PSCs. Through the rational design and synthesis of two SAMs, namely 2-(10-(3,5-dimethoxyphenyl)-7H-benzo[c]carbazol-7-yl)ethyl)phosphonic acid (abbreviated as DMPA) and its analogue BCPA, we deciphered the specific molecular functionalities governing interfacial passivation. DMPA SAM effectively suppresses self‑aggregation and enhances substrate coverage. The methoxy groups in DMPA interact with the perovskite, thereby passivating the defects at the buried interface and optimizing perovskite crystallization. This synergistic effect of structural ordering and chemical passivation yielded PSCs with a high PCE of 27.59% (certified PCE is 27.2%). The devices also exhibited remarkable photothermal stability, retaining 94.5% of their initial efficiency after 1600 hours of continuous illumination under 1 Sun at 65 °C. We therefore postulate that tuning terminal groups in SAM is a promising key strategy for developing high-performance inverted PSCs.

## Methods

### Materials

Materials were obtained from commercial suppliers and used as received without purification. N,N-dimethylformamide (DMF, anhydrous, 99.8%), dimethyl sulfoxide (DMSO, anhydrous, 99.9%), isopropanol (IPA, anhydrous, 99.5%), hydrogen peroxide (H_2_O_2_), and ethyl acetate (EA, anhydrous, 99.8%) were purchased from Sigma-Aldrich. Methylammonium chloride (MACl, 99%), cesium iodide (CsI, 99.9%), lead (II) iodide (PbI_2_, 99.99%), (2-(9H-carbazol-9-yl)ethyl) phosphonic acid (2PACZ, 98%), and piperazine dihydroiodide (PDI, 99.99%) were sourced from TCI. NiO_x_ nanoparticle powder, C_60_, and formamidinium iodide (FAI, 99.9%) were obtained from Advanced Election Technology Company (China). Methylammonium iodide (MAI) and bathocuproine (BCP) were purchased from Xi’an Polymer Light Technology Corp. Additionally, 3,6-dibromocarbazole was acquired from Adamas, 3,5-dimethoxyphenylboronic acid (Cat No. 1024705) from Leyan (Shanghai, China), diethyl 1,2-bromoethylphosphonate and 1,2-dibromoethane from Macklin, and deionized water and MgF_2_ from Bositai Corp.

### Synthesis of 10-(3,5-dimethoxyphenyl)-7H-benzo[c]carbazole (2)

Compound 1 (1 g, 3.3 mmol), 3,5-dimethoxyphenylboronic acid (0.73 g, 4 mmol), and potassium carbonate (1.85 g, 9.9 mmol) were dissolved in a mixed solvent of dioxane (50 mL) and water (5 mL) at a ratio of 10:1. A catalytic amount of tetrakis(triphenylphosphine)palladium(0) (128 mg, 0.03 mmol) was then added, and the mixture was stirred at 90 °C for 24 hours. The reaction mixture was extracted with ethyl acetate and saturated brine. The organic layer was collected, dried over anhydrous sodium sulfate, filtered, and concentrated under reduced pressure. Purification by silica gel column chromatography using a 1:3 eluent of dichloromethane and petroleum ether yielded white solid Compound 2 (0.8 g, yield 67%). ^1^H NMR (600 MHz, DMSO-*d*_*6*_) δ 11.89 (s, 1H), 8.92 – 8.86 (m, 1H), 8.75 (s, 1H), 8.10 – 8.05 (m, 1H), 7.97 – 7.93 (m, 1H), 7.79 (d, J = 8.7 Hz, 1H), 7.75 (d, J = 9.6 Hz, 3H), 7.52 – 7.47 (m, 1H), 6.97 (s, 2H), 6.54 (s, 1H), 3.89 (s, 6H). ^13^C NMR (151 MHz, DMSO-*d*_6_) δ 161.35, 144.44, 138.86, 138.52, 132.69, 129.88, 129.58, 129.06, 127.72, 127.58, 124.09, 123.90, 123.65, 123.24, 120.07, 114.61, 113.99, 112.40, 105.96, 98.81, 66.85, 55.79, 26.84.

### Synthesis of (2-(10-(3,5-dimethoxyphenyl)-7H-benzo[c]carbazol-7-yl)ethyl) phosphonic acid (4) (abbreviated as DMPA)

A mixture of the white compound 2 (400 mg, 1.13 mmol), diethyl 2-bromoethylphosphonate (332 mg, 1.35 mmol), and potassium carbonate (234 mg, 1.69 mmol) was dissolved in DMSO. The resulting solution was heated to 110°C and stirred overnight to complete the reaction. After cooling, the reaction mixture was extracted with ethyl acetate and washed with saturated brine. The organic phase was collected and subsequently purified by column chromatography using a mixture of ethyl acetate and petroleum ether (EA:PE = 2:1) as the eluent, affording compound 3 as a pale-yellow oil (420 mg, 72%). Compound 3 was added to anhydrous dichloromethane. Trimethylsilyl bromide was then added at room temperature, and the mixture was stirred overnight. Upon completion of the reaction, it was quenched by pouring into methanol. The mixture was then concentrated under reduced pressure until only a small amount of liquid remained. Water was added, resulting in the formation of a white precipitate. After standing for 5 minutes, the product 4 was isolated by filtration, yielding 310 mg (83%). ^1^H NMR (600 MHz, DMSO-*d*_*6*_) δ 8.93 (d, J = 8.3 Hz, 1H), 8.79 (s, 1H), 8.11 (d, J = 8.0 Hz, 1H), 8.06 (d, J = 8.9 Hz, 1H), 7.90 (s, 1H), 7.84 (d, J = 8.6 Hz, 1H), 7.82 – 7.76 (m, 2H), 7.55 – 7.50 (m, 1H), 6.99 (s, 2H), 6.56 (s, 1H), 5.77 (s, 1H), 4.77 (q, J = 8.0 Hz, 2H), 2.14 (dt, J = 17.6, 8.0 Hz, 2H). ^13^C NMR (151 MHz, DMSO) δ = 161.36, 144.18, 138.58, 138.22, 133.17, 129.75, 129.63, 129.12, 128.01, 127.84, 124.37, 123.73, 123.52, 120.39, 114.72, 111.79, 110.49, 106.06, 98.89, 55.81, 55.42, 40.44, 40.30, 40.16, 40.03, 39.89, 39.75, 39.61, 38.20, 28.84, 27.97. HRMS (ESI⁺) m/z calcd for C_26_H_24_NO_5_P [M + H]⁺ 462.1465, found 462.1466 (Δ = 0.22 ppm).

### Synthesis of compound (2-(7H-benzo[c]carbazol-7-yl)ethyl)phosphonic acid (7) (abbreviated as BCPA)

The commercially available white compound 5 (400 mg, 1.8 mmol), diethyl 2-bromoethylphosphonate (541 mg, 2.2 mmol), and potassium carbonate (381 mg, 2.76 mmol) were dissolved in DMSO. The resulting solution was heated to 110°C and stirred overnight to complete the reaction. After cooling, the reaction mixture was extracted with ethyl acetate and washed with saturated brine. The organic phase was collected and purified by column chromatography using a mixture of ethyl acetate and petroleum ether (EA:PE = 2:1) as the eluent, affording compound 6 as a pale-yellow oil (450 mg, 64%). Compound 6 was then added to anhydrous dichloromethane. Trimethylbromosilane was added at room temperature, and the mixture was stirred overnight. Upon completion, the reaction was quenched by pouring into methanol. The mixture was concentrated under reduced pressure until only a small amount of liquid remained. Water was added, leading to the formation of a white precipitate. After standing for 5 minutes, the product 7 was isolated by filtration (350 mg 91%). ^1^H NMR (600 MHz, DMSO-*d*_*6*_) δ 8.82 (d, J = 8.3 Hz, 1H), 8.65 (d, J = 7.9 Hz, 1H), 8.10 (d, J = 8.0 Hz, 1H), 8.03 (d, J = 8.8 Hz, 1H), 7.89 (d, J = 8.9 Hz, 1H), 7.74 (d, J = 8.0 Hz, 2H), 7.59 – 7.48 (m, 2H), 7.39 (t, J = 7.5 Hz, 1H), 4.75 (q, J = 7.9 Hz, 2H), 2.17 – 2.09 (m, 2H). ^13^C NMR (151 MHz, DMSO) δ = 138.83, 137.64, 129.72, 129.67, 129.03, 127.73, 127.65, 124.83, 123.39, 123.33, 123.26, 122.38, 120.49, 114.52, 111.71, 110.13, 40.44, 40.30, 40.16, 40.02, 39.88, 39.74, 39.60, 38.06, 28.81, 27.95. HRMS (ESI⁺) m/z calcd for C_18_H_16_NO_3_P [M + H]⁺ 326.0941, found 326.0942 (Δ = 0.31 ppm).

### Precursor solutions preparation

NiO_x_ (10 mg) was dissolved in 1 mL of deionized water containing 30 µL of H_2_O_2_. 2.4 mg SAMs (control, DMPA, and BCPA; 2.4 mg) was dissolved in 4 mL of IPA. The perovskite precursor was formed by dissolving CsI (22.1 mg), MAI (27 mg), FAI (248.5 mg), PbI_2_ (822.9 mg), and MACl (10.3 mg) in a solvent mixture of DMF (800 µL) and DMSO (200 µL). PDI (4 mg) was dissolved in 4 mL of IPA. All solutions were shaken for 4 hours and filtered through a 0.22 µm poly(tetrafluoroethylene) (PTFE) membrane.

### Fabrication of perovskite solar cells (PSCs)

ITO glass substrates were cleaned sequentially with detergent, deionized water, acetone, and IPA, followed by ultraviolet ozone treatment for 25 minutes. A NiO_x_ layer was spin-coated at 5000 rpm for 30 seconds and annealed in ambient air at 150 °C for 10 minutes. SAMs were then spin-coated onto the NiO_x_ layer at 5000 rpm for 30 seconds and annealed in nitrogen (N_2_) at 100 °C for 10 minutes. The perovskite layer was deposited by spin-coating the precursor solution at 1000 rpm for 10 seconds (acceleration rate of 2500 rpm/s) and 4000 rpm for 40 seconds (acceleration rate of 6000 rpm/s), with ethyl acetate (EA) added as an anti-solvent at the 17th second. The film was annealed in N_2_ at 100 °C for 30 minutes. Subsequently, the PDI layer was spin-coated at 4500 rpm for 25 seconds and annealed at 100 °C for 5 minutes. Finally, C_60_ (24 nm), BCP (4 nm), and an Ag electrode (100 nm) were deposited by thermal evaporation. An anti-reflection layer of MgF_2_ (thickness approximately 160 nm) was applied to the glass side via thermal evaporation.

### Calculations

First-principles pseudopotential calculations were carried out utilizing projector augmented-wave pseudopotentials implemented in the Vienna ab initio Simulation Package. For the exchange-correlation functional, the Perdew-Burke-Ernzerhof approximation was employed. A cutoff energy of 450 eV was set for the plane-wave basis set, and a Γ-centered Monkhorst-Pack k-point grid with a 2 × 2 × 1 configuration was utilized. The convergence threshold for electronic energy was set to 1 × 10^−5 ^eV, while structural relaxation was performed until the force acting on each atom was reduced to below 0.02 eV/Å. All calculations incorporated van der Waals interactions, which were treated using the Grimme DFT-D3 method. To streamline the computational process, four-layer slabs were constructed to represent the NiO_x_ surface with the bottom three layers fixed, while a three-layer slab model was built for the perovskite surface, where the bottom two layers were constrained to simulate a semi-infinite substrate. A vacuum layer of 15 Å was set over the slab structure to avoid spurious interactions due to periodic boundary conditions.

Electrostatic potential surface potential calculations were performed using the Gaussian 09 package. All computations employed the hybrid B3LYP exchange-correlation functional, a widely utilized choice for molecular system studies. The def2-TZVP basis set was used throughout to ensure adequate numerical precision.

MD simulations are as follows. Molecular models of 2PACz (Control), DMPA, and BCPA were constructed separately, with each molecule adsorbed on the NiO_x_ surface. The model system had dimensions of 85 Å × 25 Å × 85 Å. The three systems considered for control, DMPA, and BCPA consisted of 12,127, 12,617, and 12,587 atoms, respectively. Classical MD simulations with full atomic resolution were performed using the LAMMPS code. The integration of the equations of motion employed the velocity Verlet algorithm, with a time step of 1 fs. To maintain isothermal conditions, a Nosé-Hoover thermostat with a time constant of 0.1 fs was used. The simulations were conducted in the NVT ensemble, with constant number of particles, volume, and temperature at a temperature of 300 K. Periodic boundary conditions were applied in the x and z directions, while reflective boundary conditions were used in the y direction. The system was first equilibrated for 1 nanosecond, followed by a 10 ns production simulation. During this period, sampling was performed every 500 fs to calculate ensemble averages. For visualization purposes, snapshots of the system were captured every 1000 fs, and the results were visualized using Ovito. The all-atom version of the Consistent Valence Force Field (CVFF) was employed to describe interatomic interactions. A real-space cutoff radius of 10 Å was used for electrostatic nonbonded interactions, while the particle-particle-particle-mesh (PPPM) method. It was used with a precision of 10^−4^ (kcal mol⁻¹) Å^−1^ and was utilized to account for long-range electrostatic interactions.

### Nuclear magnetic resonance (NMR)

NMR spectra were recorded on a Bruker Avance III Ultra Shield Plus instrument at 600 MHz for 1H and 150 MHz for ^13^C. Proton (1H) NMR data are reported with multiplicity (s, singlet; d, doublet; t, triplet; q, quartet; qui, quintet; sept, septet; m, multiplet), coupling constants (J) in Hertz (Hz), and the number of protons. Carbon (^13^C) NMR spectra are reported in ppm (δ) relative to residual DMSO (δ 77.16).

### High-resolution mass spectrometry (HRMS)

Data for SAMs were obtained using a Japan Shimadzu LCMS-IT-TOF mass Spectrometer.

### Device and film characterizations

Photocurrent density–voltage (J–V) curves were measured using a solar simulator with a 450 W Xenon lamp (Newport 6279 NS) and a Keithley 2400 source meter, with an active area of 0.08 cm² defined by a calibrated mask. Light intensity was calibrated to AM 1.5 G one sun (100 mW/cm^2^) using a standard Si solar cell. External quantum efficiency (EQE) was determined with an EQE measurement system. X-ray diffraction (XRD) patterns were obtained using a PANalytical Empyrean diffractometer with Cu Kα radiation (λ = 1.5406 Å). Kelvin probe force microscopy (KPFM) was performed using an MFP-3D-BIO system. Absorption spectra were recorded with a Shimadzu UV-1800 UV-vis spectrophotometer. Ultraviolet photoelectron spectroscopy (UPS) was conducted on a Thermo-Fisher ESCALab 250Xi system with a He I (21.2 eV) discharge lamp under a pressure of 5.0 × 10^−7 ^Pa. Fourier-transform infrared (FTIR) mapping and spectra were collected using a PerkinElmer Spotlight 400 instrument. Contact angles were measured with a DSA-100 system. Steady-state photoluminescence (PL) spectra were acquired with a Cary Eclipse fluorescence spectrophotometer at an excitation wavelength of 450 nm. PLQY was measured using a LuQY Pro system from Quantum Yield Berlin. In-situ GIWAXS was performed in Beijing Synchrotron Radiation Facility and processed with software GIWAXS tools. Tensile forces were evaluated using a microcomputer-controlled ETM102B electron universal testing machine from Wance Technologies Ltd. Scanning electron microscopy (SEM) images were captured using a Thermo Scientific Quattro ESEM, with electron beam acceleration between 200 V and 30 kV. Cyclic voltammetry (CV) was carried out using a CHI760 electrochemical workstation. Electrochemical impedance spectra (EIS) were measured on a Zennium-IM6 workstation at open-circuit voltage in the dark. The stability test following the ISOS-L-2 protocol was conducted under continuous heating at ~65 °C, AM1.5 G illumination (100 mW cm^−2^, white LED), and open-circuit conditions. The device is ITO/NiO_x_/SAM/PVK/PDI/C_60_/SnO_2_/Ag. The PCEs of the devices were tracked over time, and the J–V measurements were performed after cooling the devices down to room temperature. The MPPT of the devices was conducted under AM1.5 G illumination at ~65 °C. The voltage at the maximum power point was automatically adjusted and applied, and the power output of the devices was tracked over time.

### The hole mobility analysis method

The hole mobility of ITO/NiO_x_/SAM(Control, DMPA and BCPA)/Ag is extracted by fitting the J-V curves with the Mott-Gurney Eq. (4):

4 $$J=\frac{9}{8}{\varepsilon }_{0}{\varepsilon }_{r}{{{\rm{\mu }}}}\frac{{\left(V-{V}_{r}-{V}_{{bi}}\right)}^{2}}{{L}^{3}}$$

where *J* is the current density, *ε*_*0*_ is the vacuum permittivity, *ε*_*r*_ is the dielectric permittivity of the NiO_x_/SAM, *L* is the film thickness, *V* is the applied voltage of the device, *V*_*r*_ is the voltage drop due to constant resistance and series resistance across the electrodes, *V*_*bi*_ is the built-in voltage due to the different work function of the two electrodes, and μ is the hole mobility.

### Transient absorption spectroscopy (TAs)

The system employs a 1046 nm Yb laser as the excitation source, with a laser power of 500 μJ/pulse, a pulse repetition rate (PPR) of 2 kHz, and an average output power of approximately 1 W. The pulse duration (FWHM) is 283.9 fs (as per the test report). The probe beam is generated as a white light continuum via a sapphire crystal, covering a spectral range of 480–950 nm, with optional extensions into the ultraviolet (350–650 nm), near-infrared (1100–1600 nm), and short-wave infrared (1600–2400 nm) regions.

### Quasi-fermi level splitting (QFLS)

QFLS can be calculated from PLQY by the following formula (5):

5 $${QFLS}={{QFLS}}_{{rad}}+{k}_{B}T{{\mathrm{ln}}}\left({PLQY}\right)={k}_{B}T{{\mathrm{ln}}}\left(\;\frac{{J}_{G}}{{J}_{0,{rad}}}\times {PLQY}\right)$$

Where, *QFLS*_rad_ is the radiation limit of semiconductor materials, which sets the maximum achievable splitting of the quasi-Fermi level without considering nonradiative radiation recombination. *k*_B_ is the Boltzmann constant, *T* is the thermodynamic temperature. *J*_G_ is the photogenerated current density, *J*_0,rad_ is the dark state radiative recombination saturation current density. According to the detailed balance theory, the *J*_0,rad_ can be calculated by the following Eqs. (6 and 7):

6 $${J}_{0,{rad}}=q{\int }_{0}^{\infty }{{EQE}}_{{PV}}\left(E\right){\phi }_{{BB}}\left(E\right){dE}$$

7 $${\phi }_{{BB}}\left(E\right)=\frac{2\pi {E}^{2}}{{h}^{3}{c}^{2}}\frac{1}{\exp \left(\frac{E}{{k}_{B}T}\right)-1}$$

Where *q* is the elementary charge, *EQE*_PV_ is the photovoltaic external quantum efficiency, *ϕ*_BB_ is the black-body radiative spectrum, *E* is the photo energy, *h* is the Planck constant, and *c* is the light speed in vacuum.

### Reporting summary

Further information on research design is available in the Nature Portfolio Reporting Summary linked to this article.

## Supplementary information

Supplementary Information

Reporting Summary

Transparent Peer Review file

## Source data

Source Data

## Data Availability

The data generated in this study are provided in the manuscript and Supplementary Information. The data supporting the findings of this study are provided as a Source Data file. Source data have also been deposited in the Figshare database under the link: https://doi.org/10.6084/m9.figshare.33033215. Source data are provided with this paper.
