# Supplementary material for: Self-Assembled Multilayers Reduce Interfacial Energy Loss in Perovskite Solar Cells
Source: Nat Commun. 2026 Aug 8;17:9993. doi: 10.1038/s41467-026-76497-1 (PMC13590575; doi:10.1038/s41467-026-76497-1)
Supplement: Supplementary file 1 [file 41467_2026_76497_MOESM1_ESM.pdf]

Supplementary Materials for

# Self-Assembled Multilayers Reduce Interfacial Energy Loss in Perovskite Solar Cells

Yi Pan[1†], Lei Liu[1†], Haoxuan Guo[2†], Changqin Lin[3†], Pengfei Wu[4], Zeping Ou[1], Can Wang[1], Peidong Chen[1], Qin Gao[1], Mingyang Gao[1], Xiaoxue Lin[1], Dingqin Hu[5], Tingming Jiang[1], Yujie Zheng[1], Zeyun Xiao[6], Ke Yang[6], Zeyu Zhang[7], Rui Wang[8], Nabonswende Aida Nadege Ouedraogo[1*], Zedong Lin[9*], Wei Wei[10*], Kuan Sun[1*], Qiang Liao[1*]

[1]MOE Key Laboratory of Low-grade Energy Utilization Technologies and Systems, School of Energy & Power Engineering, Chongqing University, Chongqing 400044, China

[2]Department of Chemistry and Materials Engineering, Kansai University, Suita, Osaka 564-8680, Japan

[3]Institute of Systems and Physical Biology, Shenzhen Bay Laboratory, Shenzhen, Guangdong 518055, China

[4]Wu Jieh Yee School of Interdisciplinary Studies, Lingnan University, Tuen Mun, Hong Kong 999077, China

[5]Department of Mechanical Engineering, City University of Hong Kong, Kowloon Tong, Hong Kong 999077, China

[6]Chongqing Institute of Green and Intelligent Technology, Chinese Academy of Sciences, Chongqing 400714, China

[7]School of Physics and Optoelectronic Engineering, Hangzhou Institute for Advanced Study, University of Chinese Academy of Sciences, Hangzhou 310024, China

[8]School of Engineering, Westlake University, Hangzhou 310030, China

[9]School of Materials Science and Engineering, Taizhou University, Taizhou 318000, China

[10]Key Laboratory of Optoelectronic Technology and System, Ministry of Education of China, College of Optoelectronic Engineering Chongqing University, Chongqing 400044, China

[†]These authors contributed equally to this work.

[*]Corresponding authors. E-mail: kuan.sun@cqu.edu.cn (K.S.); aida@cqu.edu.cn (N.A.N.O.); wwei@cqu.edu.cn (W.W.); zedonglin@alu.ruc.edu.cn (Z.L.); lqzx@cqu.edu.cn (Q.L.)

**The PDF file includes:**

Supplementary Figs. 1 to 22

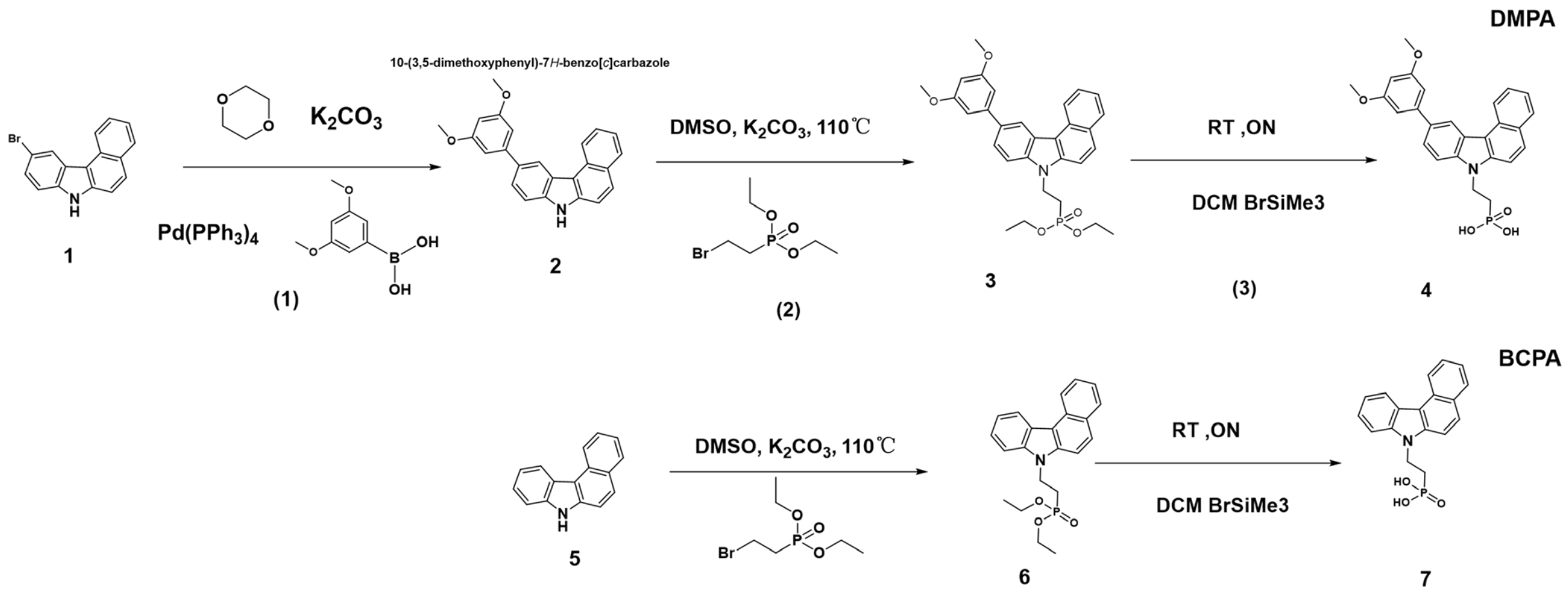


**Supplementary Figure 1 |** Synthetic routes of DMPA and BCPA.

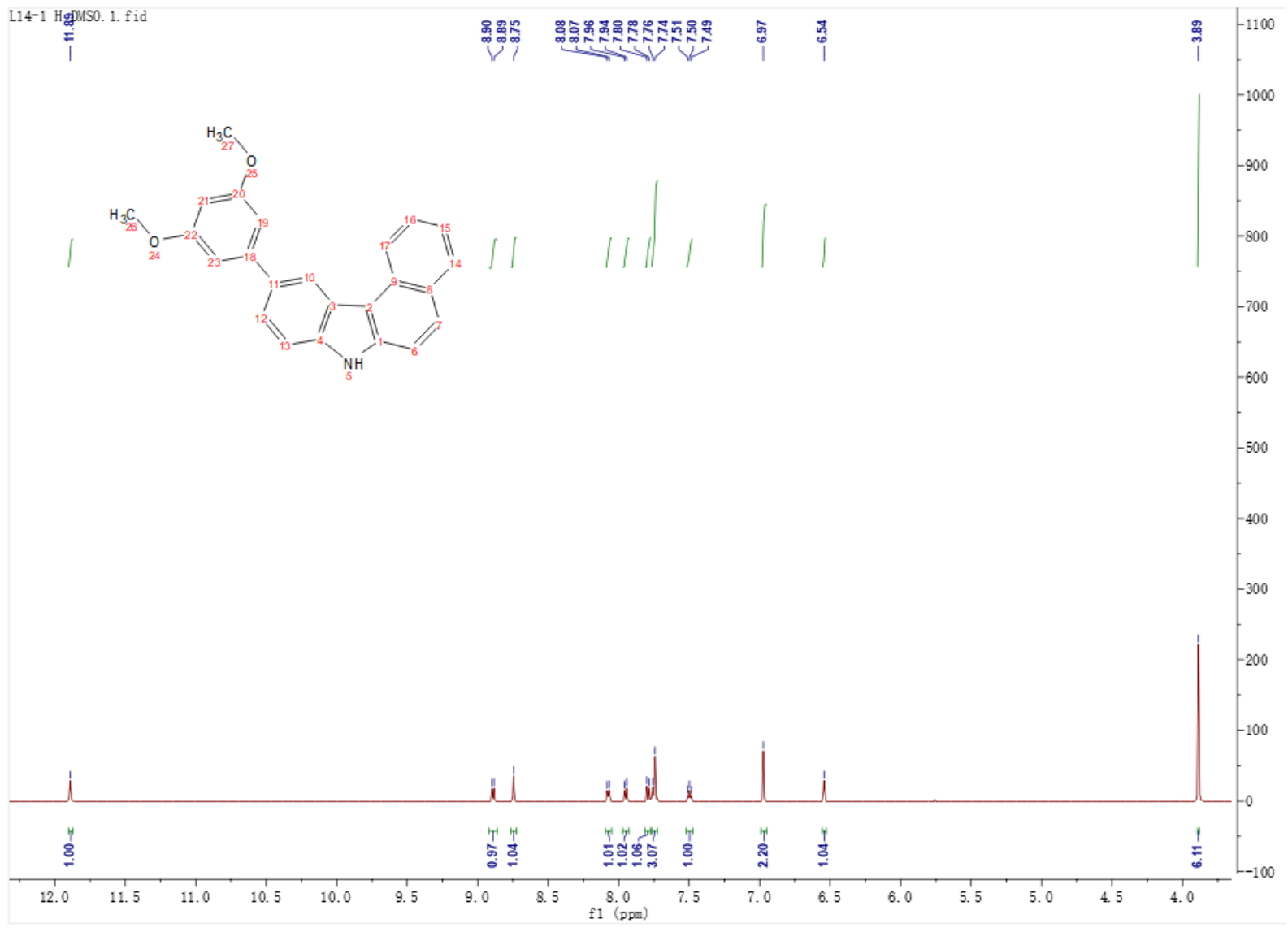

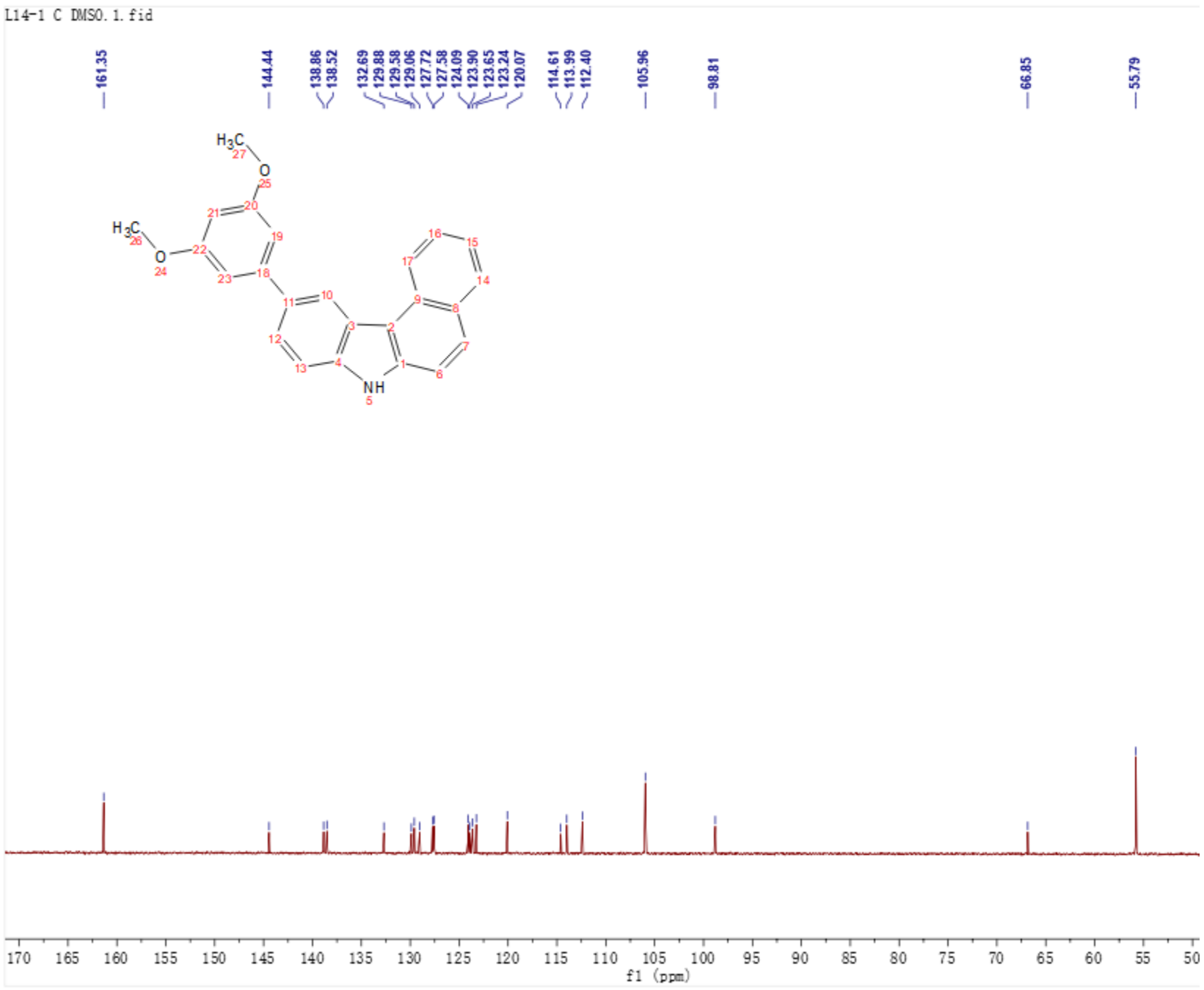

**Supplementary Figure 2 |** The hydrogen spectrum ($^{1}H$ NMR) and carbon spectrum ($^{13}C$ NMR) of compound 2.

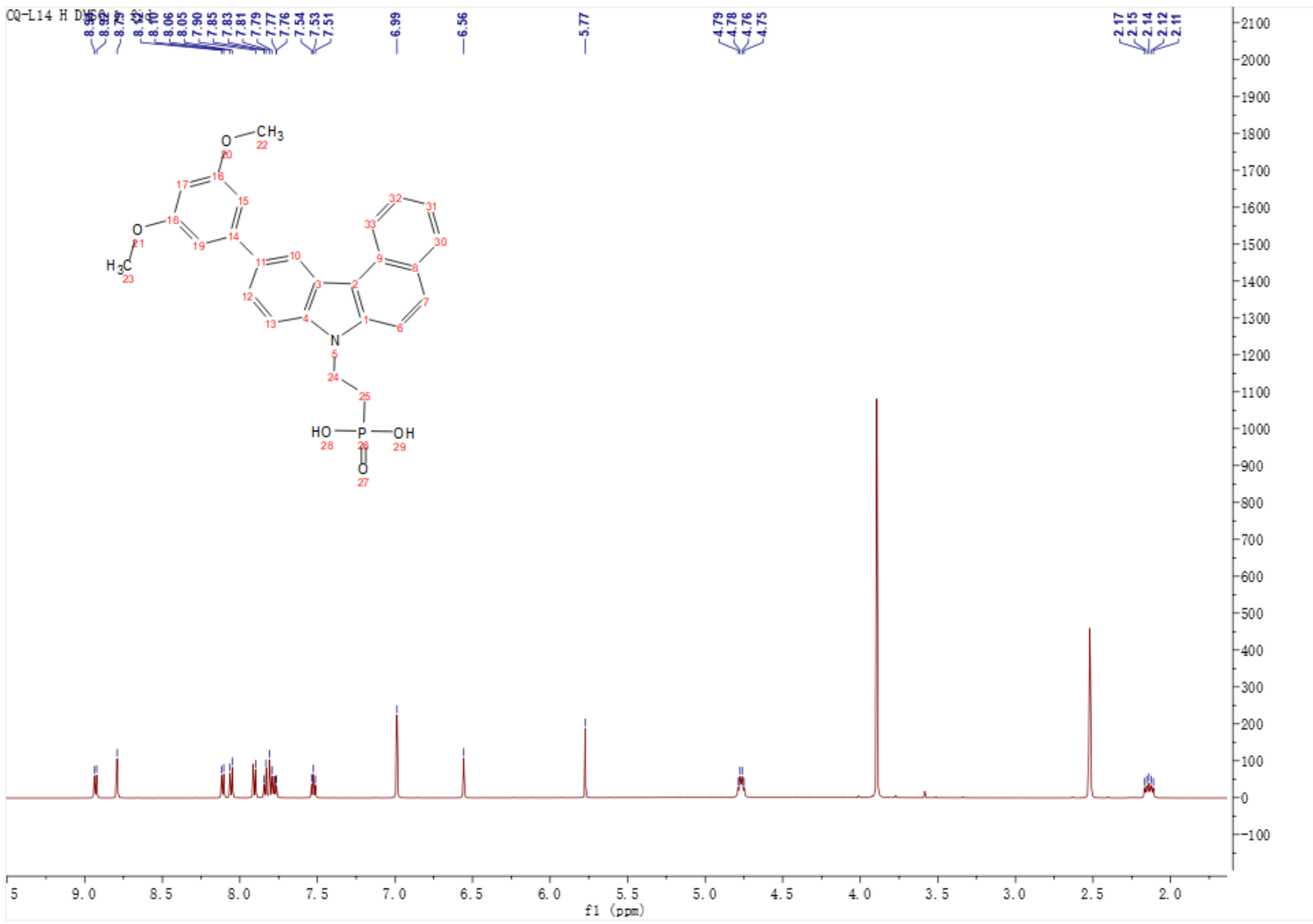


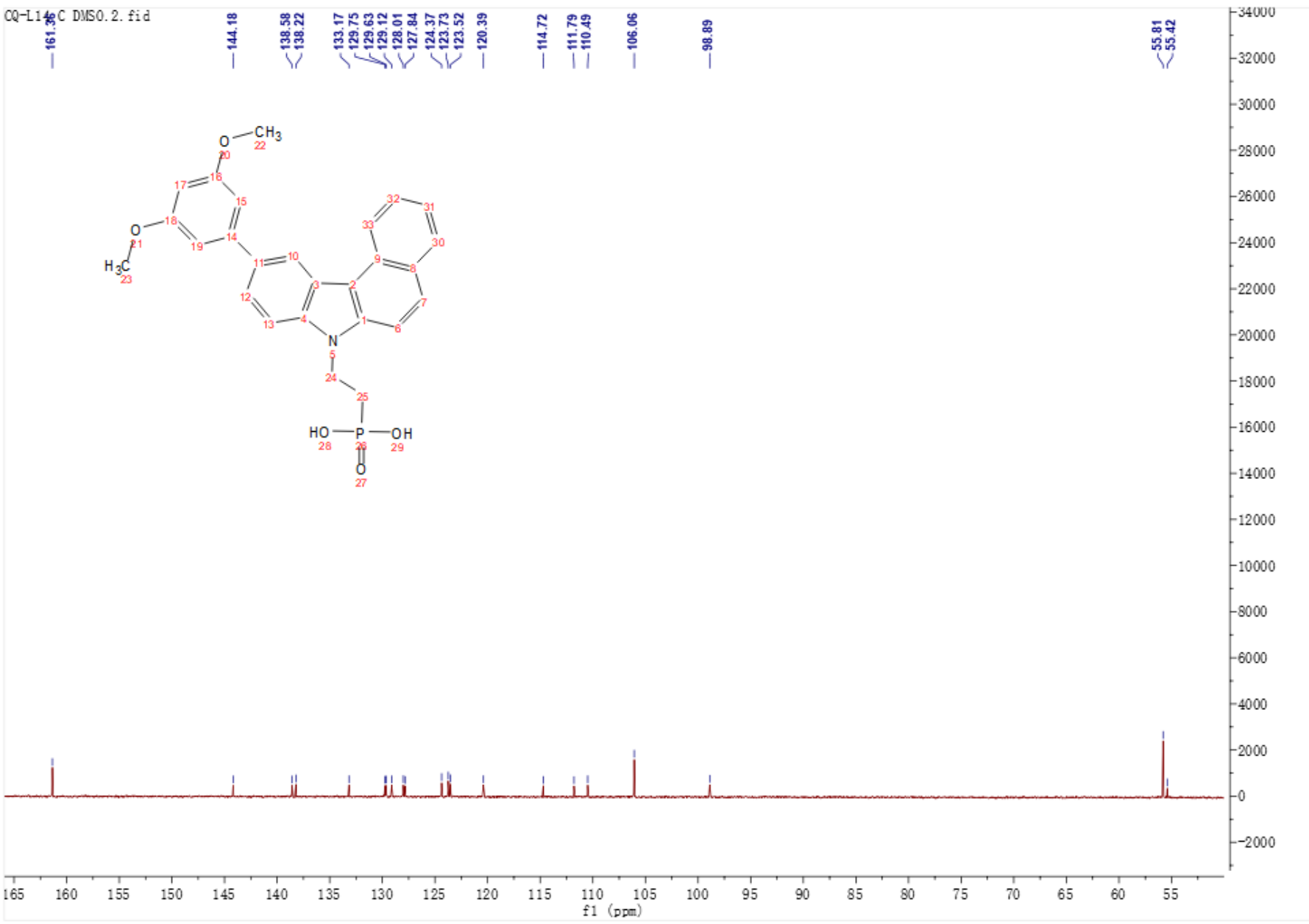


**Supplementary Figure 3 |** The hydrogen spectrum ($^1$H NMR) and carbon spectrum ($^{13}$C NMR) of compound 4.

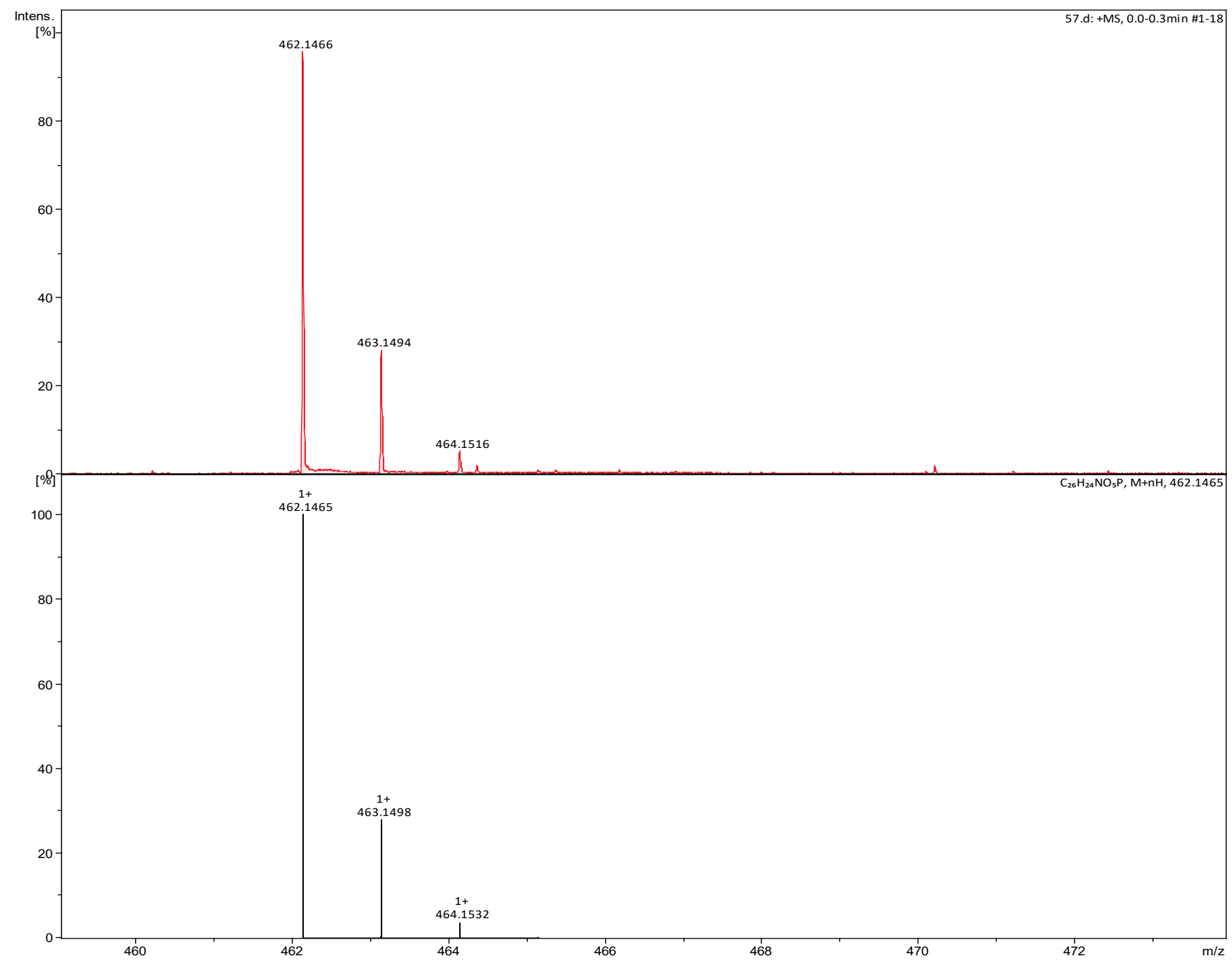


**Supplementary Figure 4 |** High-resolution mass spectrum of compound 4.

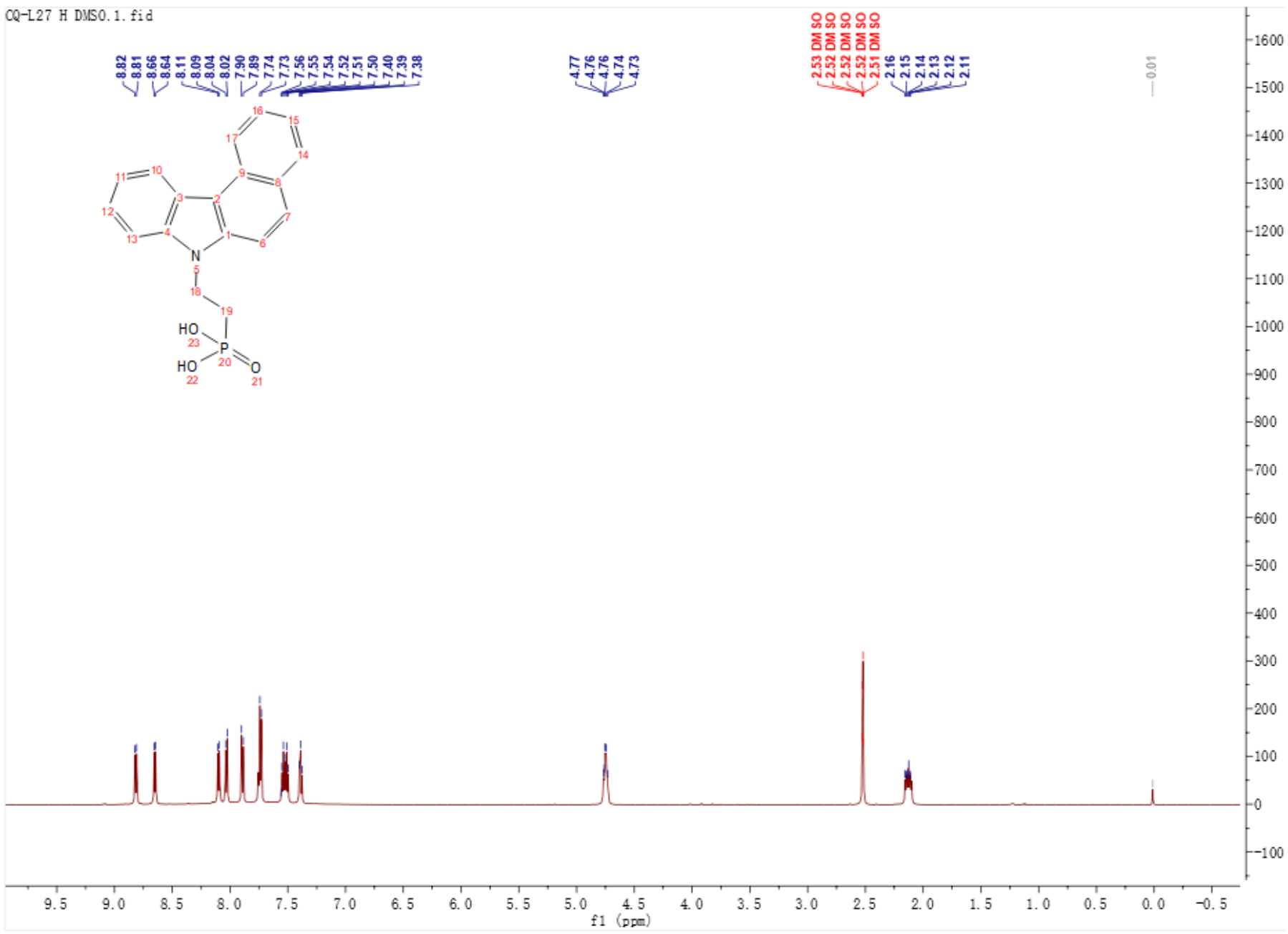


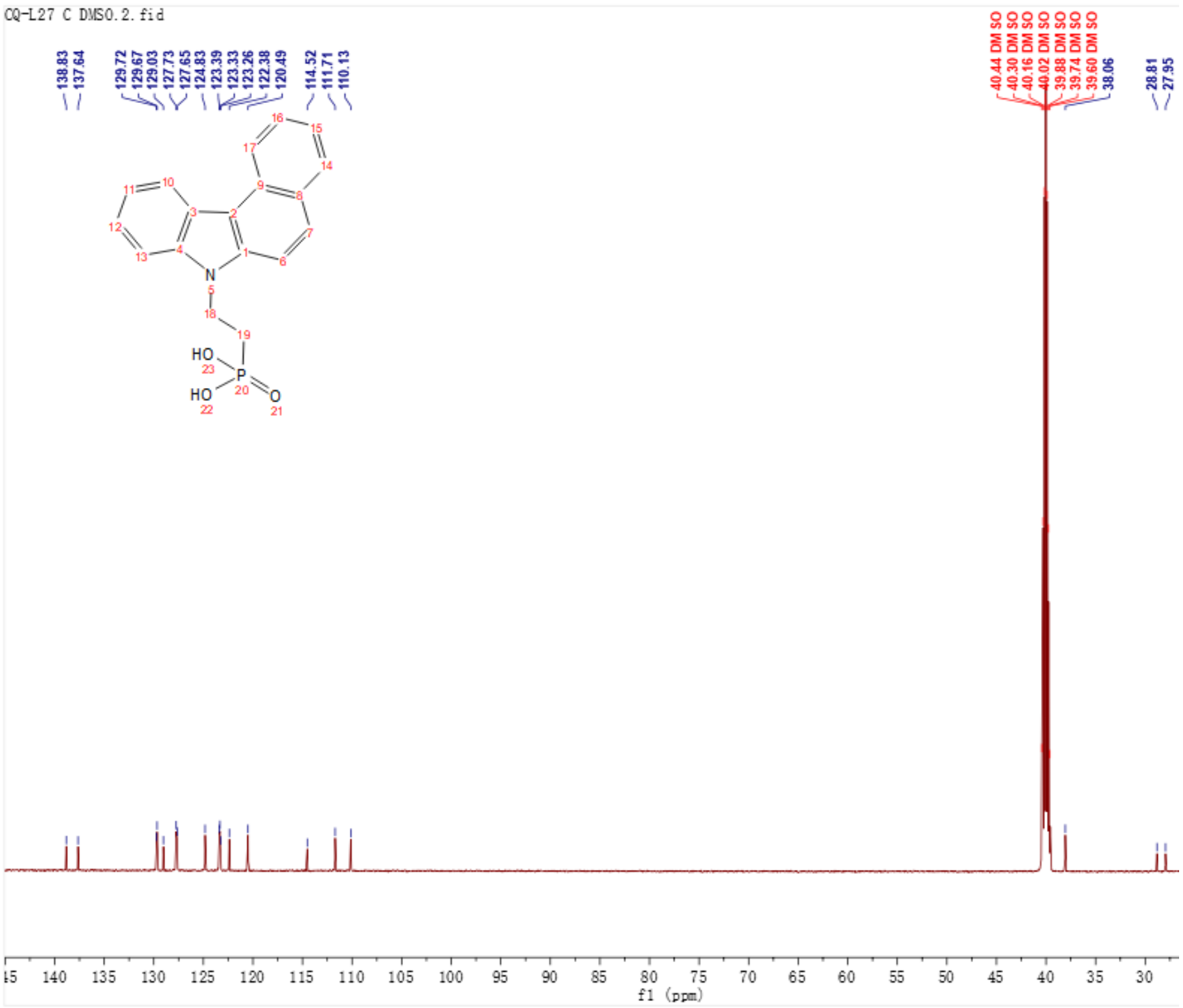


**Supplementary Figure 5 |** The hydrogen spectrum ($^1$H NMR) and carbon spectrum ($^{13}$C NMR) of compound 7.

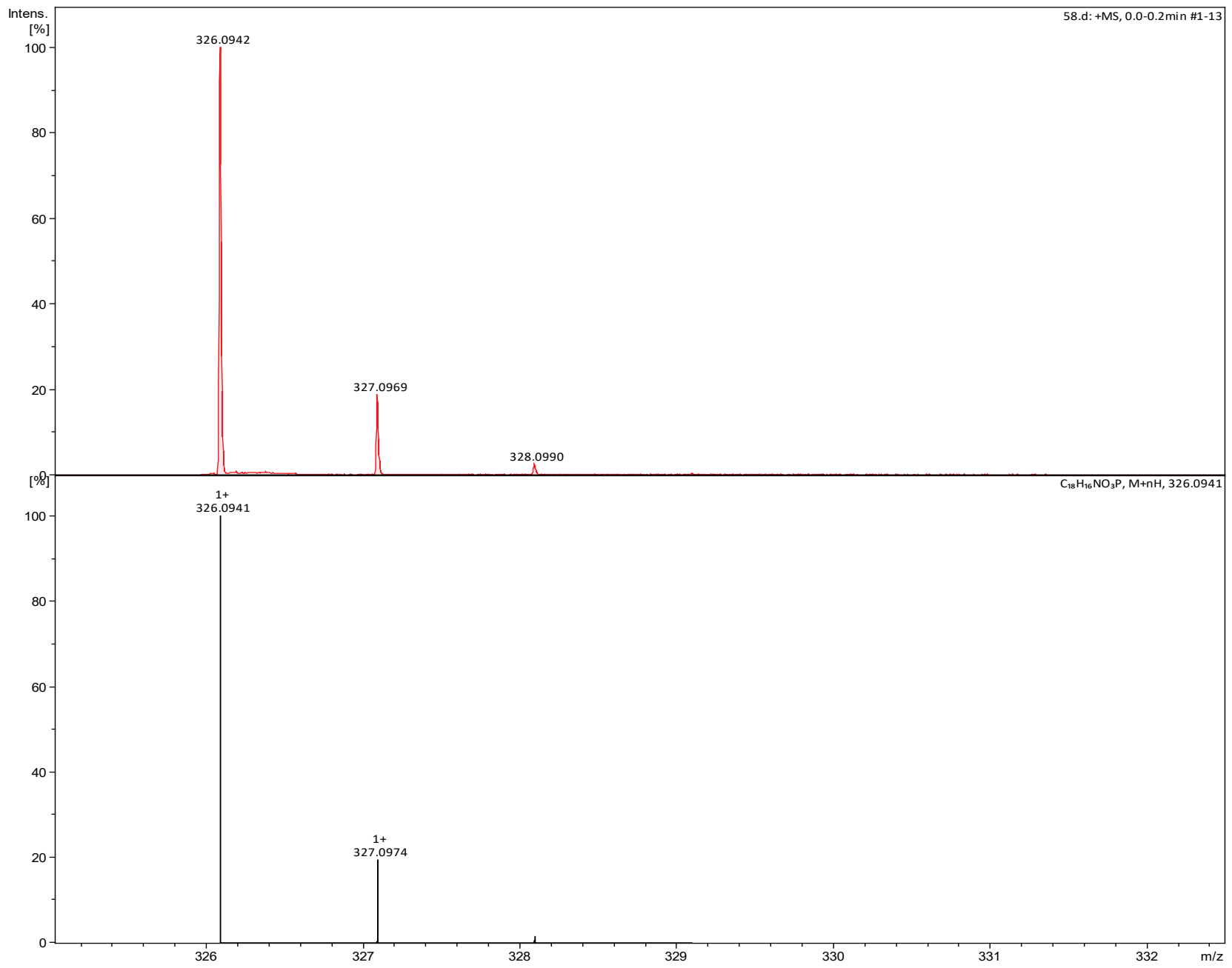


**Supplementary Figure 6 |** High-resolution mass spectrum of compound 7.

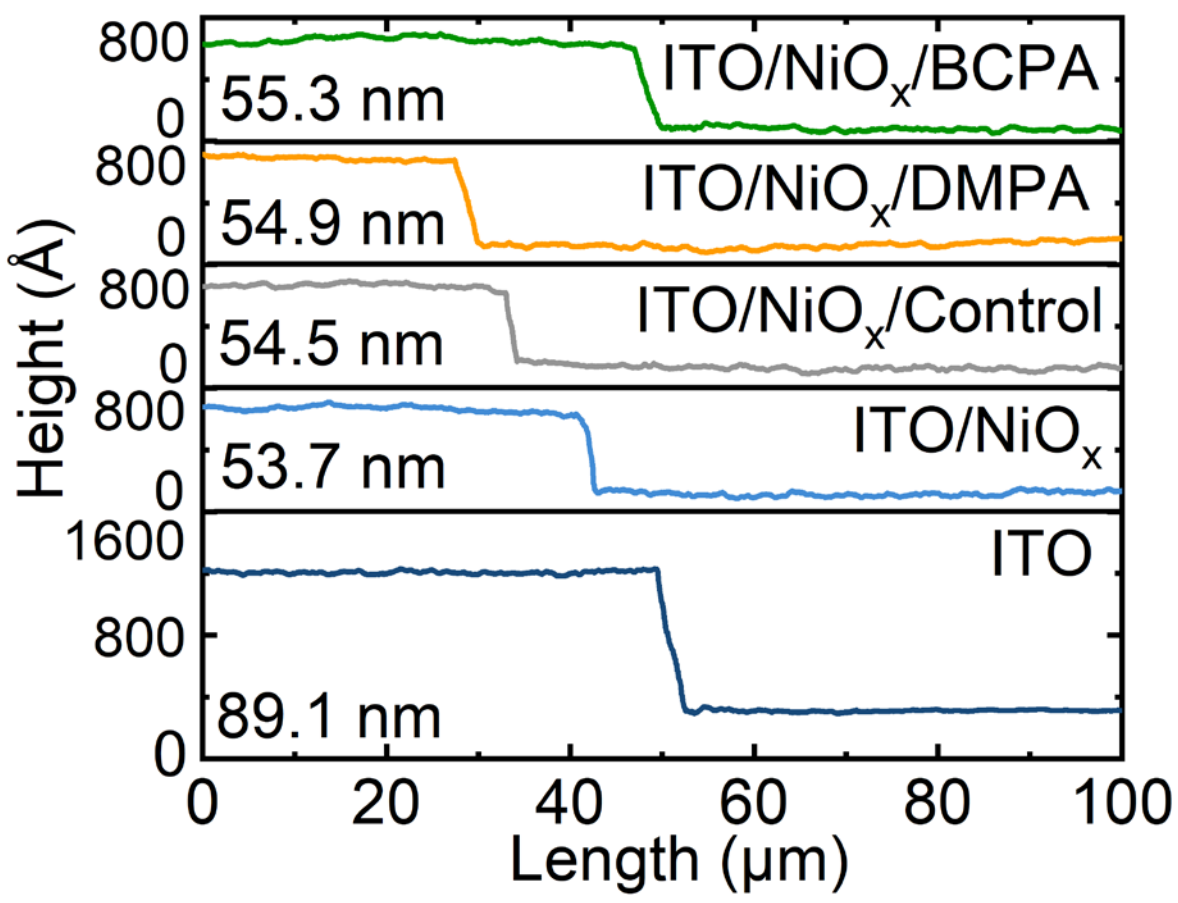


**Supplementary Figure 7 |** The thickness of different films obtained from a stylus profiler.

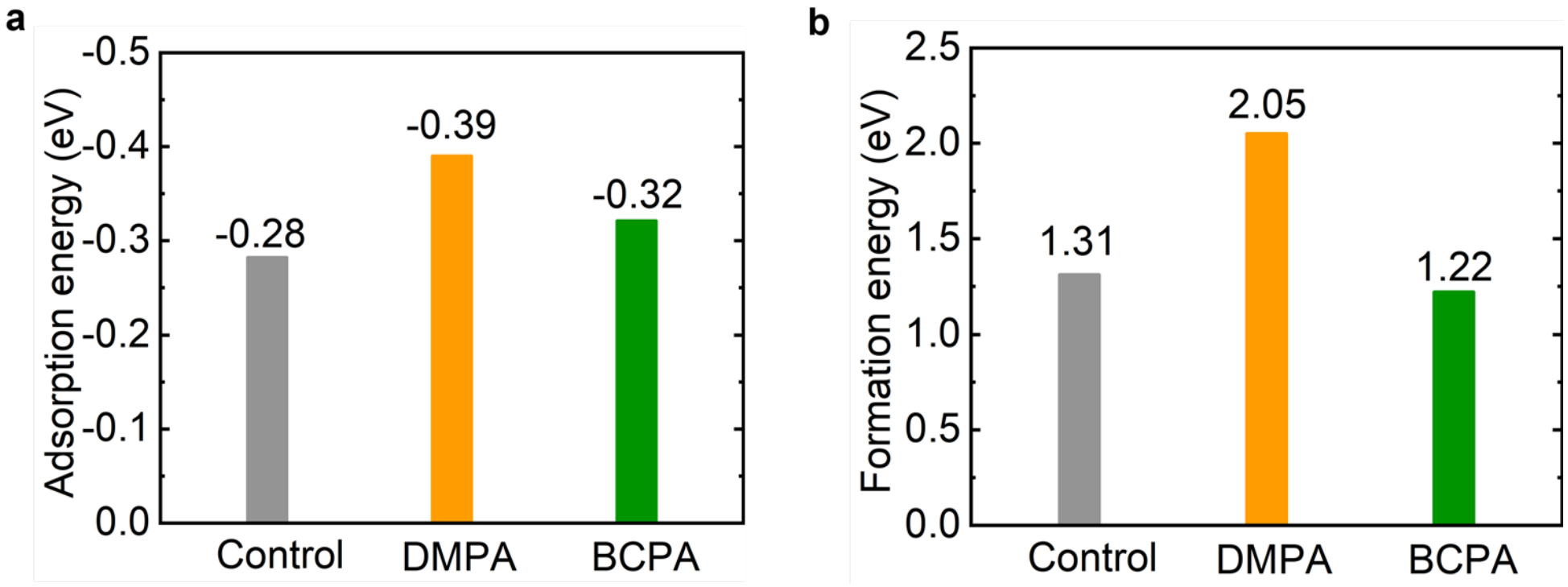


**Supplementary Figure 8 | a,** Adsorption energy ($E_{ad}$) of different SAM molecules on the perovskite surface, obtained from DFT calculations. **b,** Defect formation energies of Pb-related defects in the perovskite at the buried interface, calculated for the control, BCPA, and DMPA-modified samples.

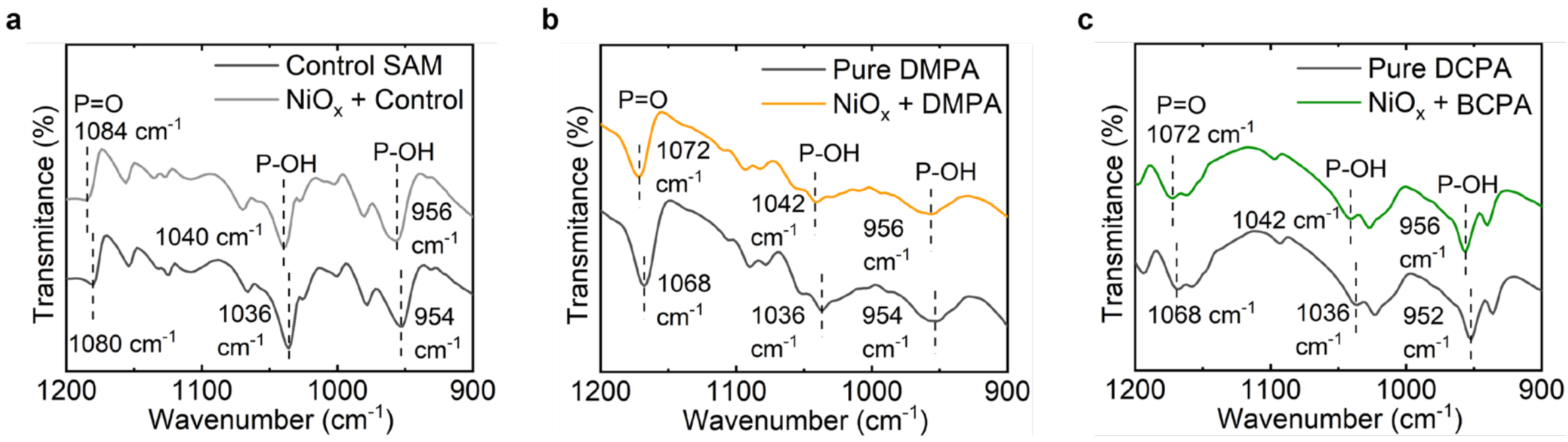


**Supplementary Figure 9 |** FT-IR spectra of **a,** ITO/$NiO_x$/control SAM; **b,** ITO/$NiO_x$/DMPA; **c,** ITO/$NiO_x$/BCPA.

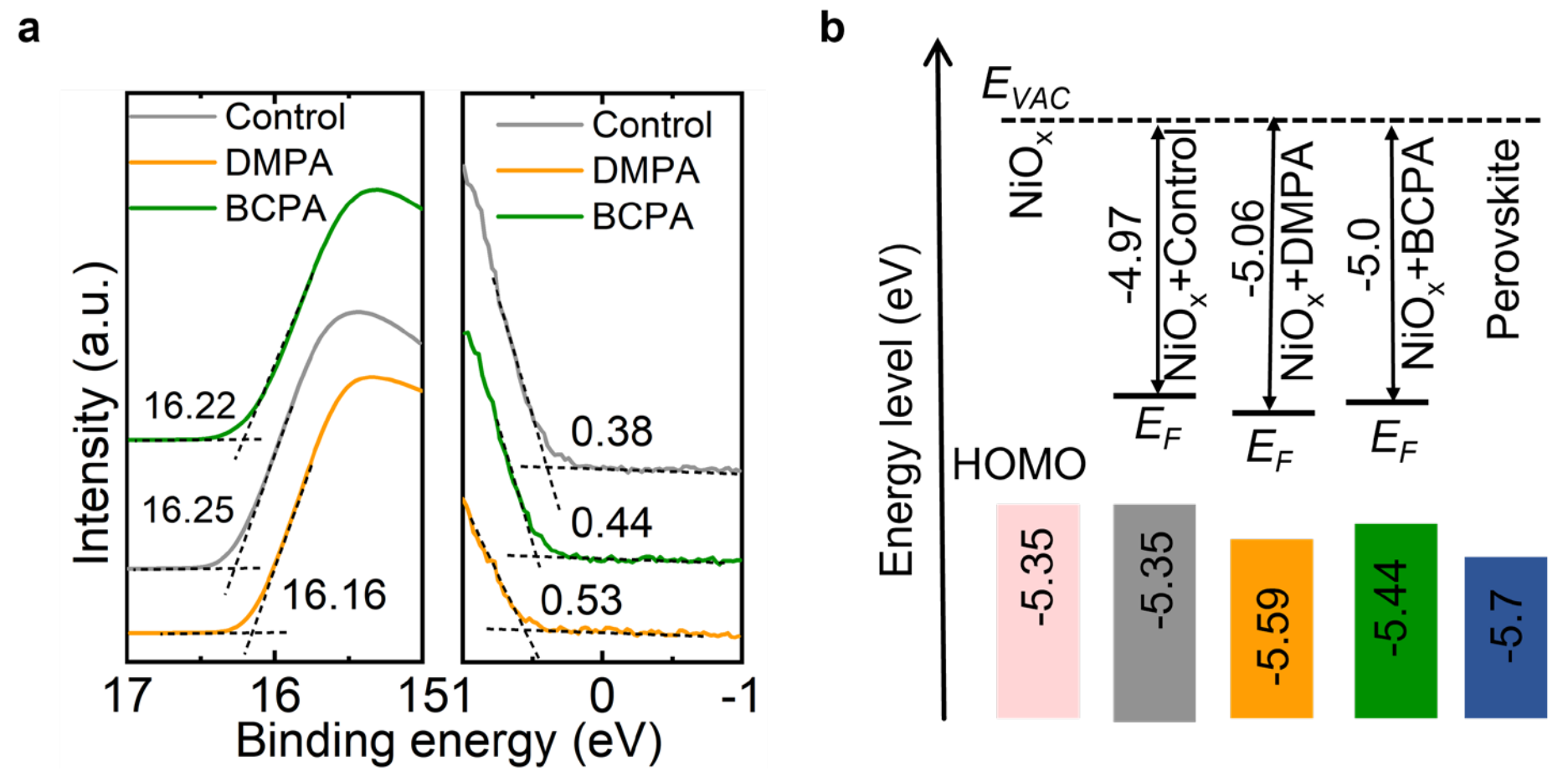


**Supplementary Figure 10 | a,** UPS spectra of $NiO_x$/SAM; **b,** Schematic diagram of energy level arrangement.

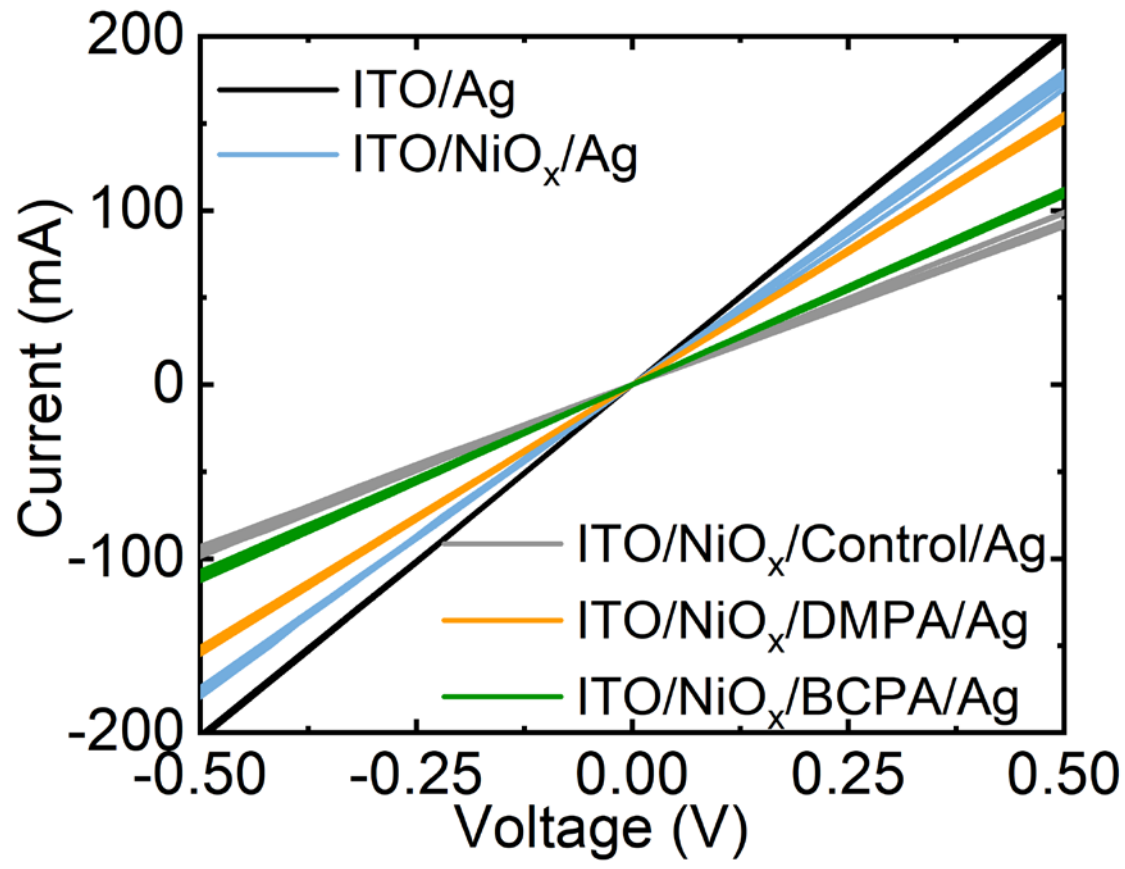


**Supplementary Figure 11 |** The J-V characteristics of different samples revealing their conductivities. Five measurements were conducted for each sample.

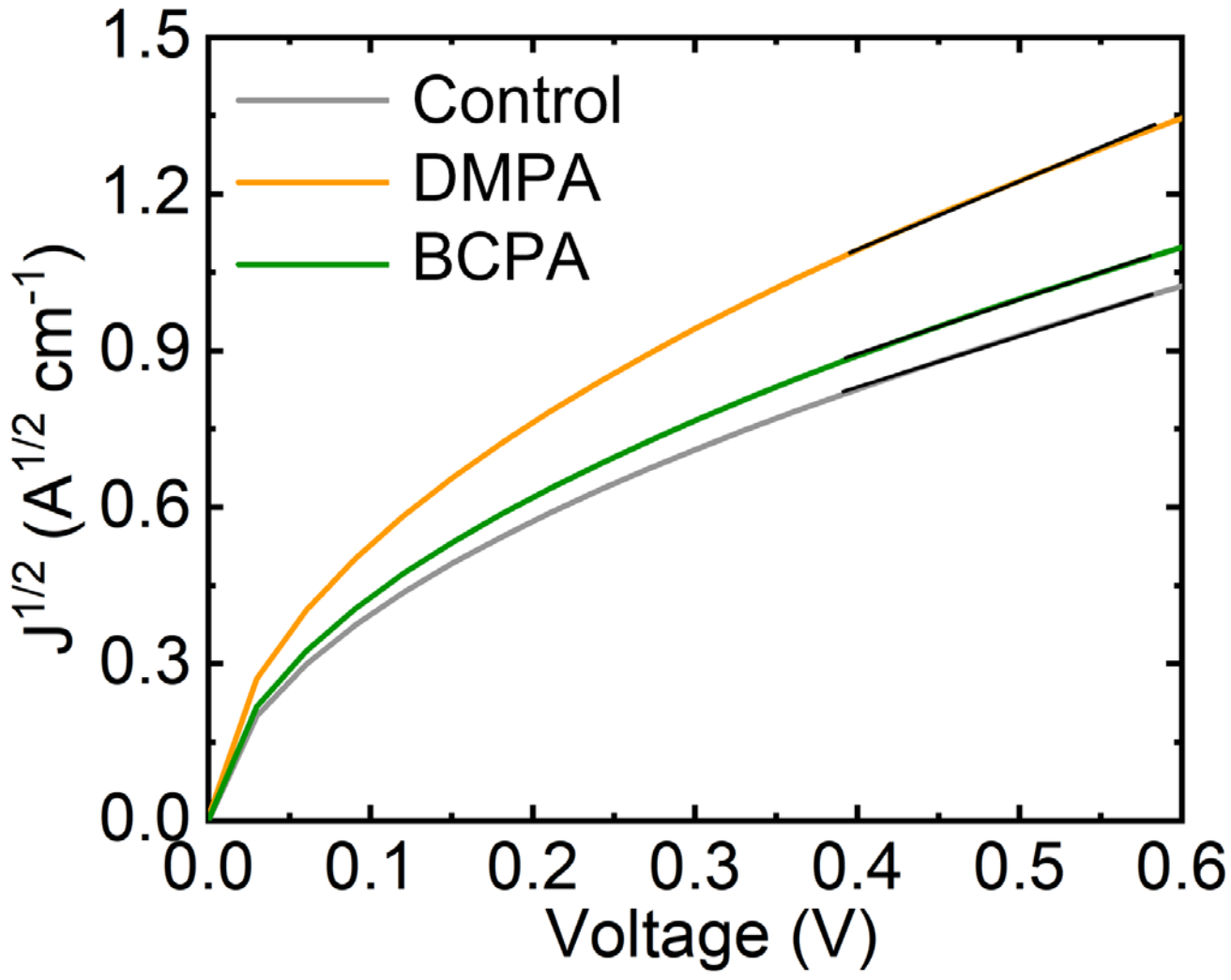


**Supplementary Figure 12 |** Hole mobilities of the two SAMs and the control were determined via SCLC measurements.

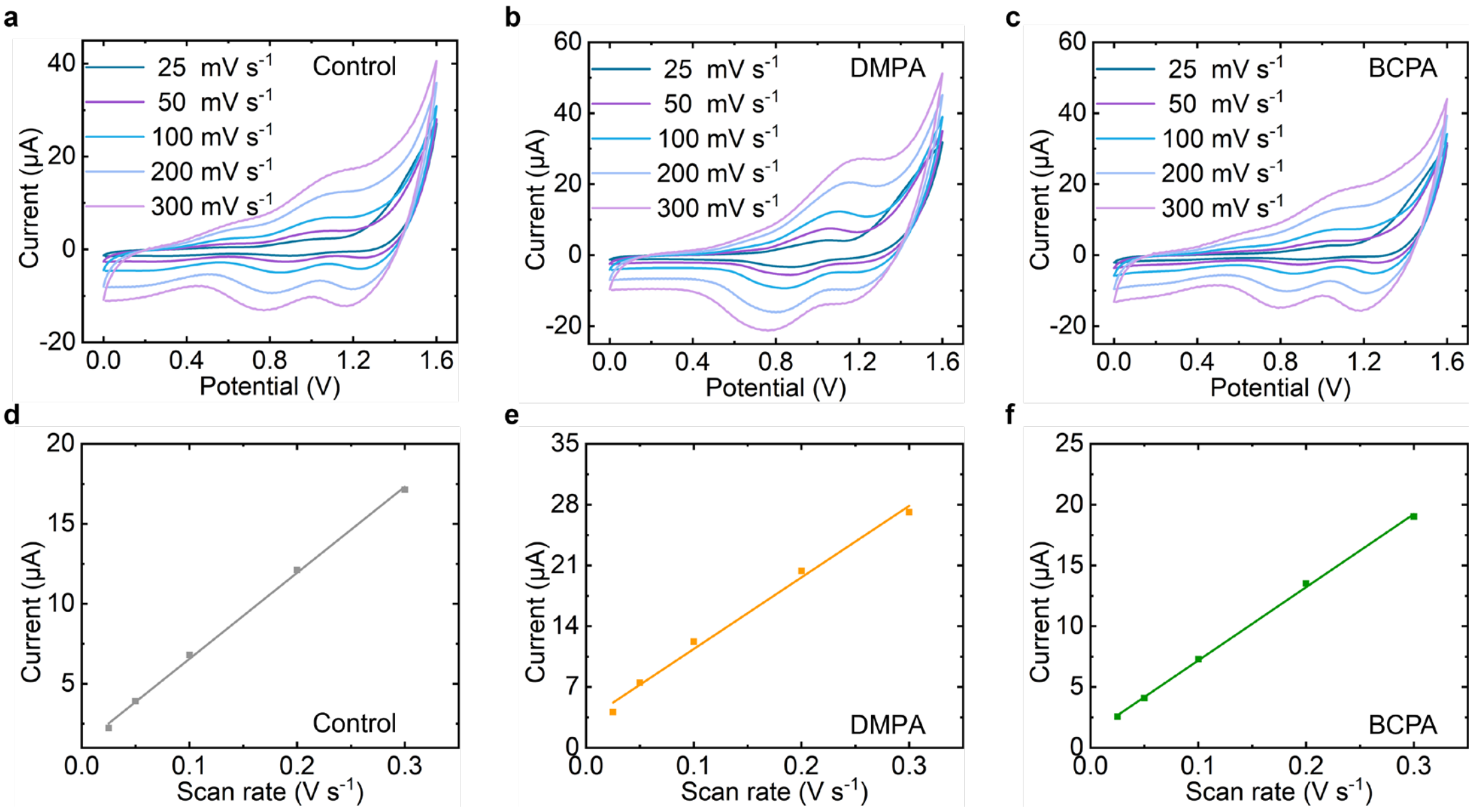


**Supplementary Figure 13 |** Cyclic voltammograms of **a,** ITO/$NiO_x$/control SAM, **b,** ITO/$NiO_x$/DMPA, and **c,** ITO/$NiO_x$/BCPA measured at varying scan rates. Relationship between peak current and scan rate for the oxidation peaks of **d,** ITO/$NiO_x$/control SAM; **e,** ITO/$NiO_x$/DMPA; **f,** ITO/$NiO_x$/BCPA in cyclic voltammetry.

.

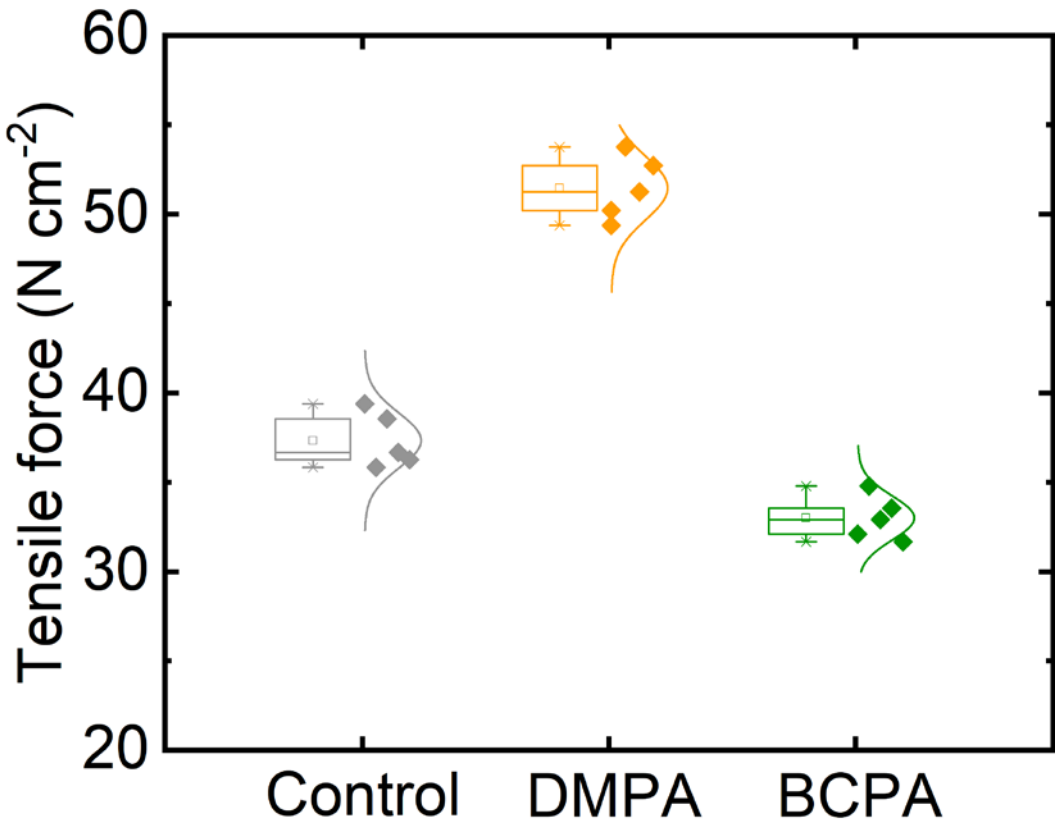


**Supplementary Figure 14 |** Mechanical tensile test results for the ITO/$NiO_x$/SAM/perovskite/UV-adhesive/glass stack. Five independent devices were measured for each SAM (control, DMPA, and BCPA). The data demonstrate the mechanical robustness and interfacial adhesion of the prepared perovskite films under tensile stress.

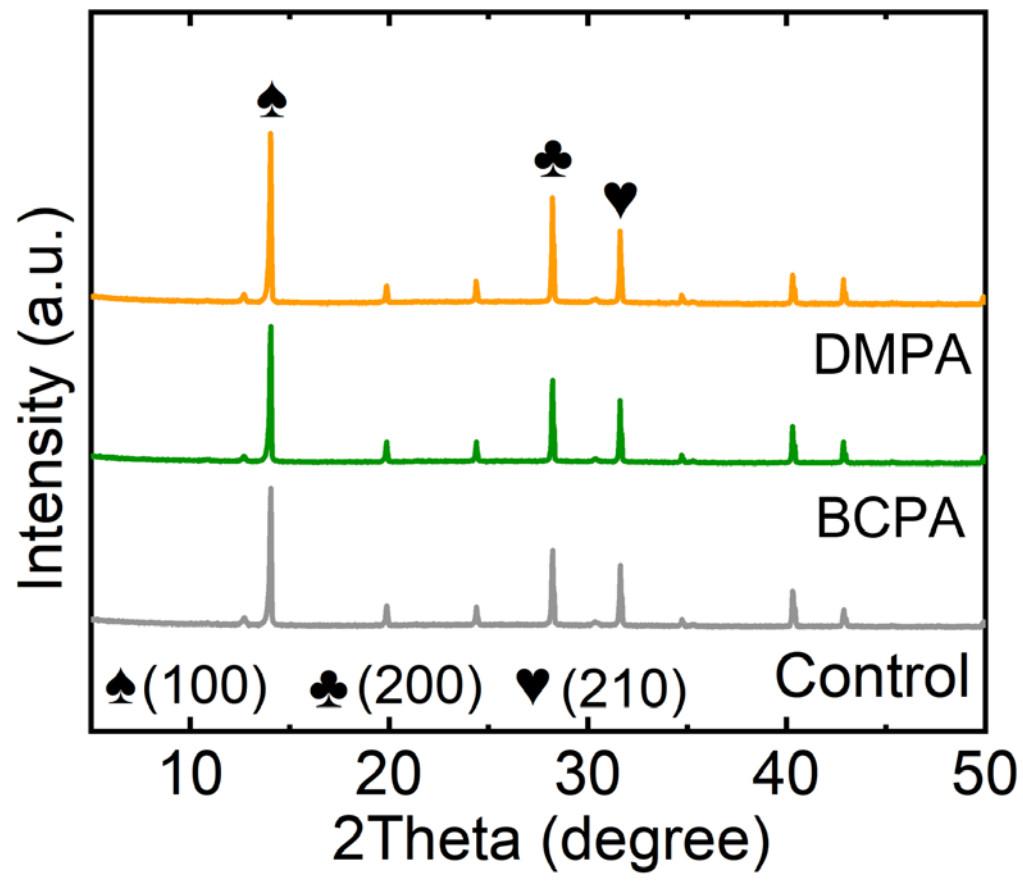


**Supplementary Figure 15 |** XRD spectra of perovskite films deposited on different ITO/$NiO_x$/SAM substrates.

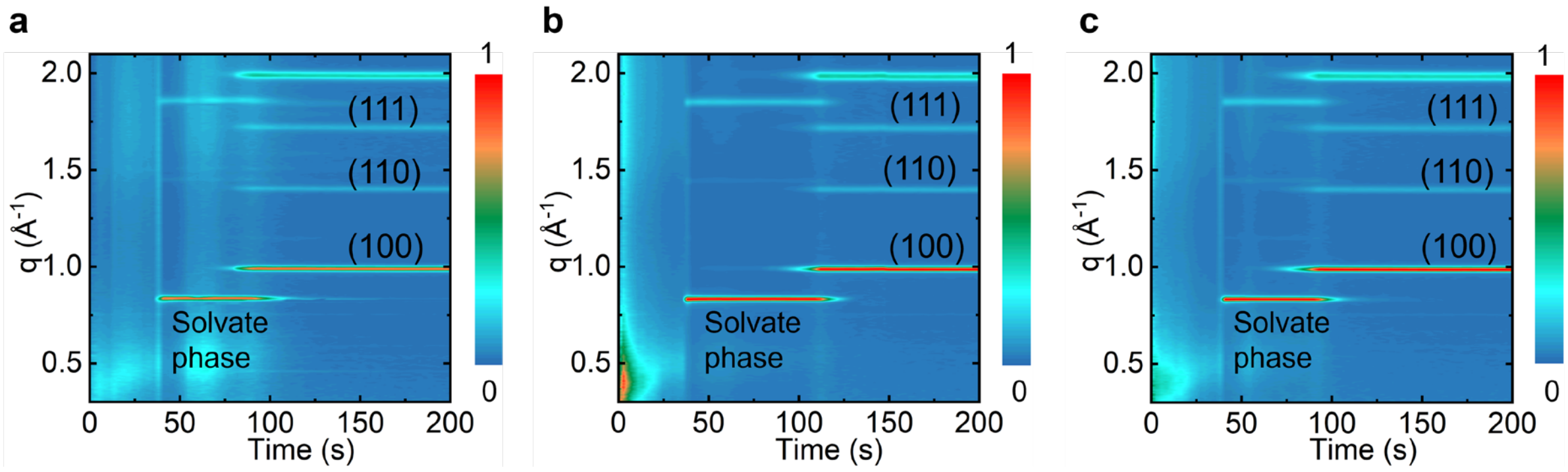


**Supplementary Figure 16 |** In situ GIWAXS false-color maps showing the diffraction intensity as a function of scattering vector q ($Å^{-1}$) and time (s) for **a,** control, **b,** DMPA-modified, and **c,** BCPA-modified perovskite films. The data track the structural evolution during spin-coating and annealing, with the characteristic peaks at q ≈ 1.00, 1.42, and 1.73 $Å^{-1}$ corresponding to the (100), (110), and (111) perovskite planes, respectively.

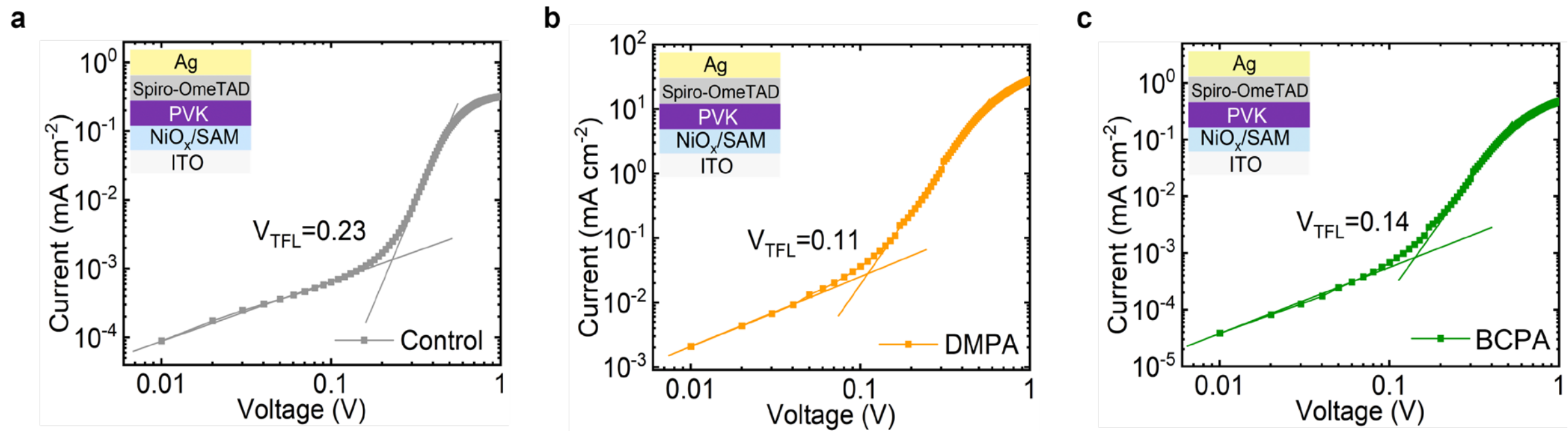


**Supplementary Figure 17 |** Space-charge limited current (SCLC) curves of hole-only devices fabricated with **a,** control, **b,** DMPA-modified, and **c,** BCPA-modified SAMs.

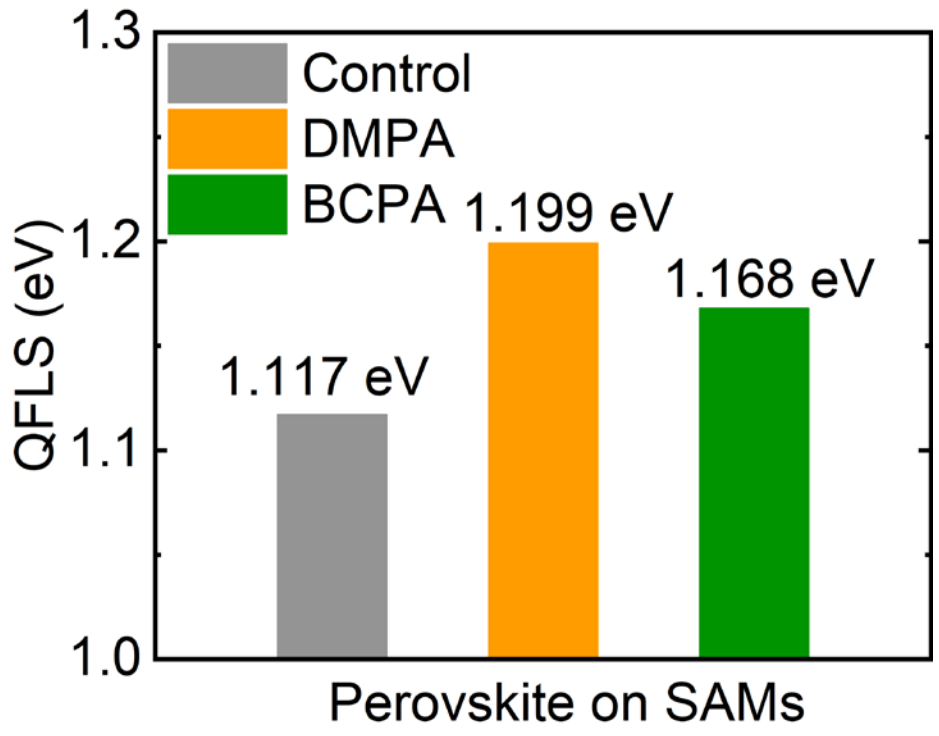


**Supplementary Figure 18 |** Quasi-Fermi-level splitting (QFLS) of perovskite films deposited on different $NiO_x$/SAM substrates.

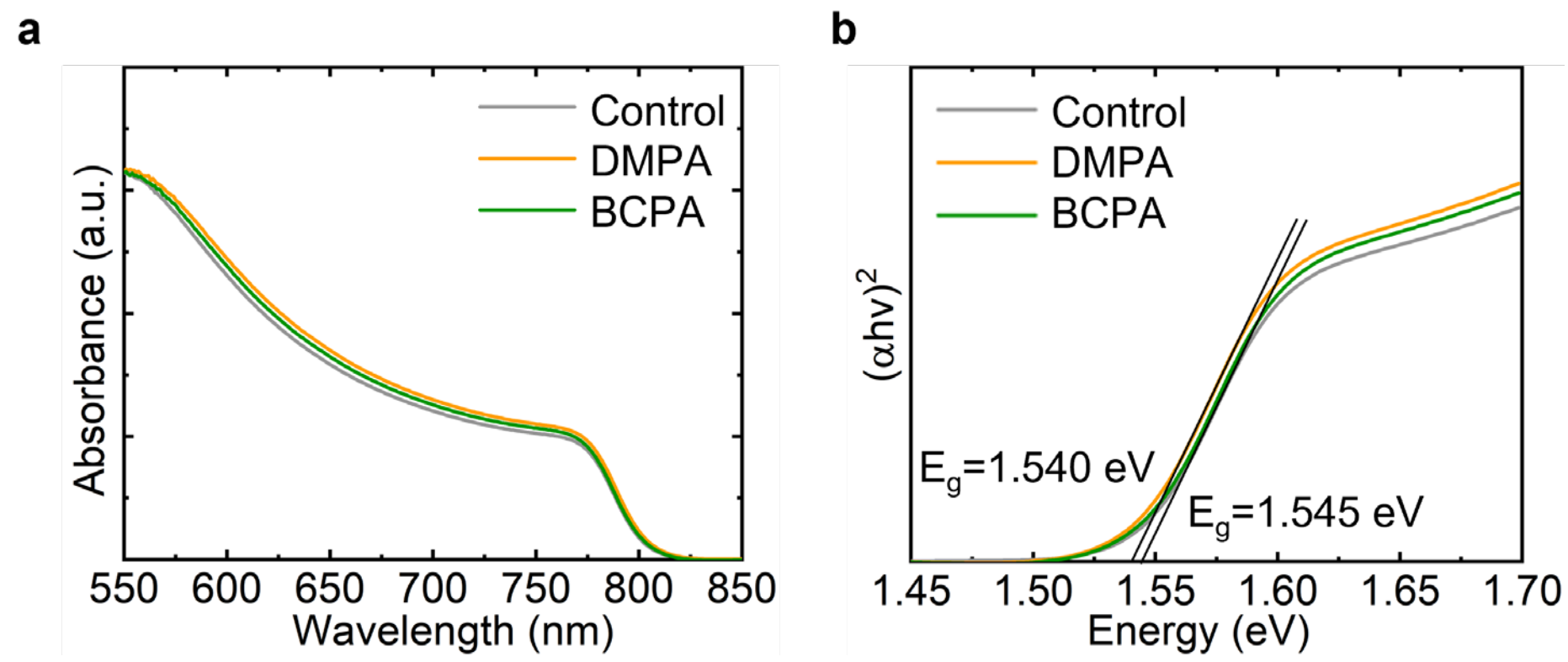


**Supplementary Figure 19 | a,** The UV-vis spectra of perovskite films and **b,** the corresponding Tauc spectra from UV-vis.

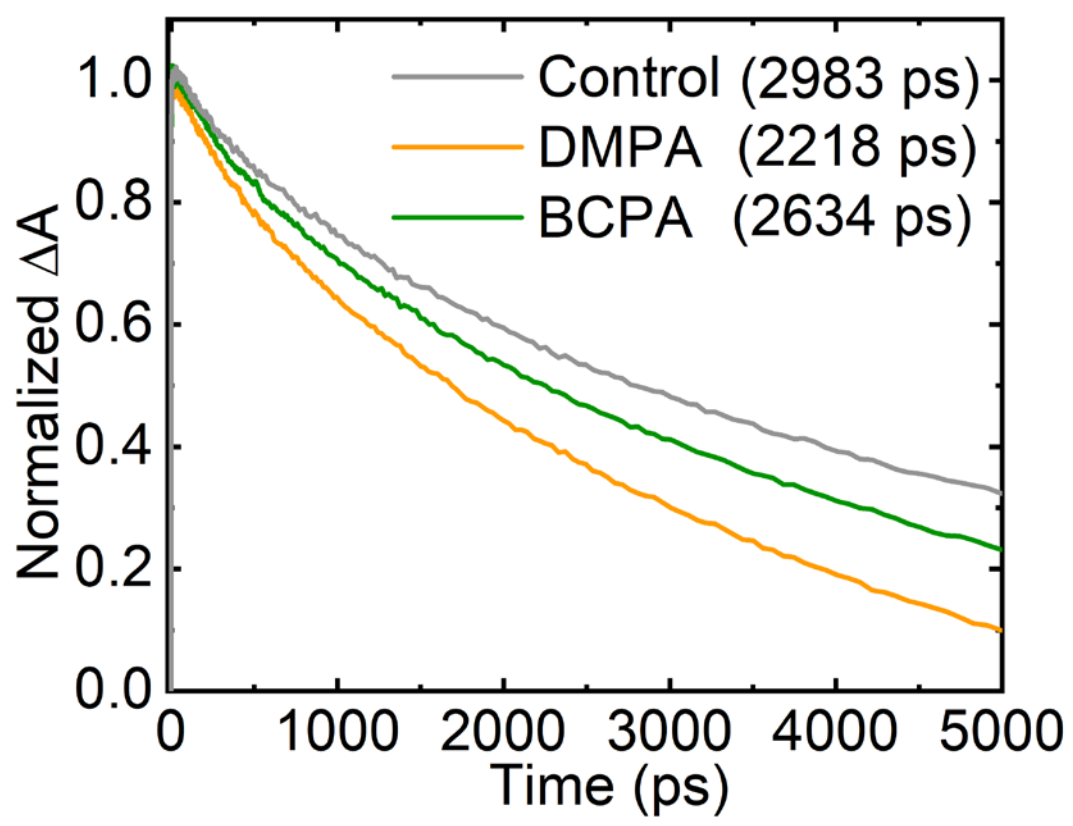


**Supplementary Figure 20 |** Normalized ground-state bleach (GSB) decay traces monitored at 803 nm for perovskite films prepared with control, DMPA, and BCPA SAMs.

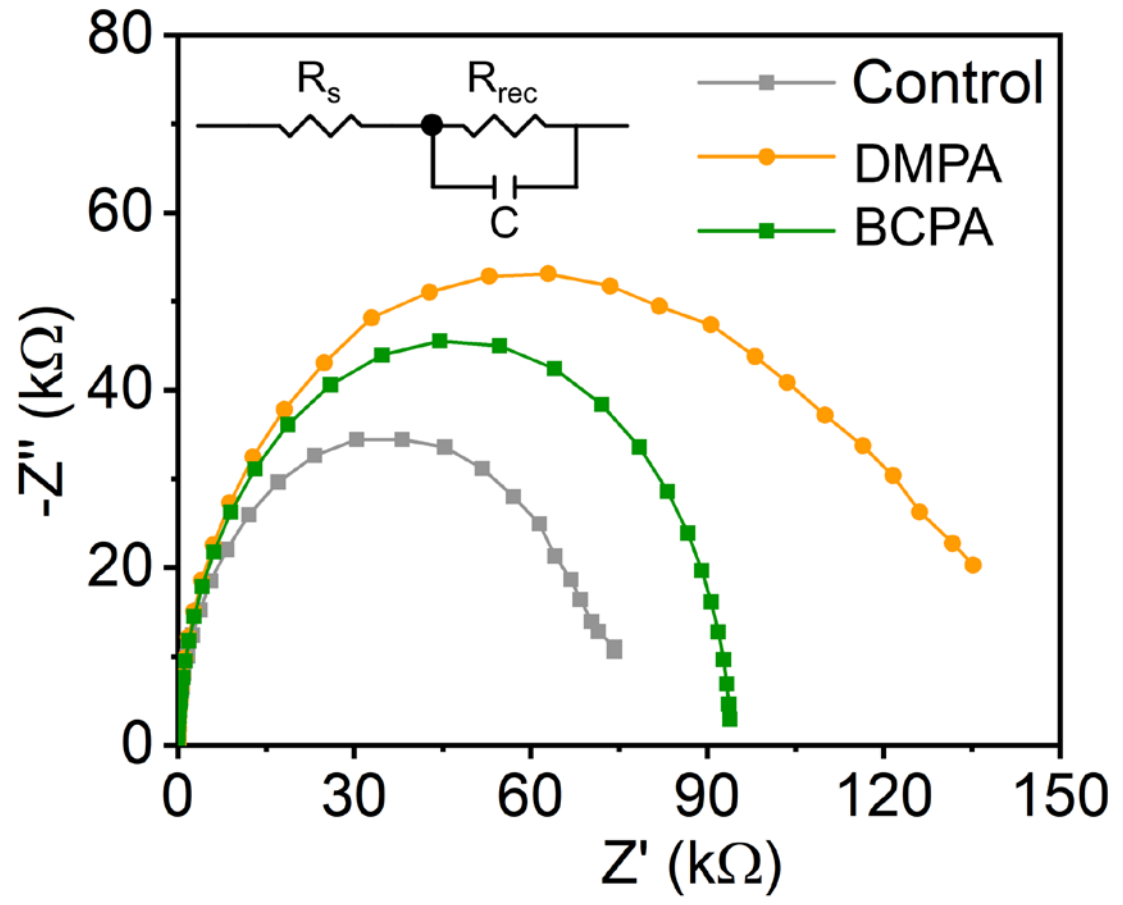


**Supplementary Figure 21 |** Nyquist plots obtained for the corresponding devices.

# 检 测 报 告

Test Report

报告编号：检测字第 202601100460 号
Report No.

防伪码
7b4b84a95c8740a6
82f49e76d9dc6ac7
10f5401a5ae34bab
ac2225ce38918e9a

| | |
|---|---|
| 样 品 名 称<br>Sample Name | FAMACsPbI3 inverted perovskite solar cell |
| 标 称 生 产 单 位<br>Manufacturer | Chongqing University |
| 委 托 单 位<br>Client | Chongqing University |
| 联 络 信 息<br>Contact Information | No.174, Shazheng Subdistrict, Shapingba District, Chongqing, China |
| 检 测 类 别<br>Test Category | Commission test |

（检测专用章）
Stamp

1010450901

授权签字人 陈满满
Approved by

签发日期 2026 年 01 月 23 日
Issue Date Year Month Day

地址：中国・四川・成都玉双路 10 号
Address: No.10, Yushuang Road, Chengdu, Sichuan, China
邮编：610021
Post Code
网址：www.nimtt.cn
Web
电话：028-60828828
Telephone
传真：028-84404149
Fax
邮箱：kfzx@nimtt.com
E-mail

# 声　明

1. 本单位仅对加印“中国测试技术研究院检测专用章”的完整证书负责。

2. 报告无主检人、审核人、授权签字人完整签字无效。

3. 报告涂改无效。

4. 对送样的委托检测报告，检测结果仅对来样负责。

5. 如样品由委托方提供，委托方对样品相关信息的真实性负责。

6. 客户若对本报告有异议，应在收到报告五个工作日内向我单位提出书面意见。

| | | | |
|---|---|---|---|
| 样品名称<br>Sample Name | FAMACsPbI3 inverted perovskite solar cell | 型号规格<br>Model | Single-junction Perovskite solar cell |
| 商　标<br>Trade Mark | / | 质量等级<br>Grade | / |
| 生产日期<br>Production Date | / | 出厂编号/批号<br>Serial No. or Lot No. | CQU-LaFREMD-F9 |
| 受检单位 Institution Inspected — 名称 Name | / | | |
| 受检单位 Institution Inspected — 地址 Address | / | | |
| 标称生产单位 Manufacturer — 名称 Name | Chongqing University | | |
| 标称生产单位 Manufacturer — 地址 Address | No.174, Shazheng Subdistrict, Shapingba District, Chongqing, China | | |
| 委托单位 Client — 名称 Name | Chongqing University | | |
| 委托单位 Client — 地址 Address | No.174, Shazheng Subdistrict, Shapingba District, Chongqing, China | | |
| 抽样方案<br>Sampling Plan | / | 抽样单编号<br>Sampling list No. | / |
| 抽样人员<br>Sampler | / | 抽样日期<br>Sampling Date | / |
| 抽样地点<br>Sampling Site | / | 抽样数量<br>Sampling Quantity | / |
| 抽样基数<br>Basic Number for Sampling | / | 到样日期<br>Receipt Date | 2026 年 01 月 23 日 |
| 送样数量<br>Sample Quantity | 1 | 样品状态<br>Sample Status | Intact |
| 检测地点<br>Test Location | No.10, Yushuang Road, Chengdu, Sichuan, China | 检测日期<br>Test Date | 2026 年 01 月 23 日 |
| 检测依据<br>Test Method | IEC 60904-1:2020 光伏器件 第 1 部分:光伏电流-电压特性的测量 | | |

# Results of Test

## 1. Test Condition

Reference Cell: Mono-Si solar cell (window material: KG1) .

Sample Information: Perovskite solar cells. Dimensions:(1.5×1.5) $cm^2$. Aperture area:0.083 $cm^2$.

Environmental Condition: Temperature: 25±5℃; humidity: 30±10%.

## 2. Methodologies and Settings

(1) In the 3A steady-state solar simulator (spectrum: AM1.5), the irradiance of the solar simulator was first calibrated to 1000 $W/m^2$ with a standard solar cell, the temperature of the solar cell was controlled by a water bath thermostat at 25℃, and then the I-V parameters of the tested sample were measured with a digital source meter.

(2) The parameter settings of the measurement software are shown in Table 1:

Table 1 Parameter Settings for I-V Test

| Scan Mode | Start Voltage(V) | End Voltage(V) | Step (V) | Sweep point delay(ms) | Number of point | Light Soaking Pre-treatment |
|---|---|---|---|---|---|---|
| Reverse scan | 1.2 | -0.1 | 0.01 | 20 | 130 | YES |
| Forward scan | -0.1 | 1.2 | 0.01 | 20 | 130 | YES |

## 3. Test Results

Current-voltage (I-V) curves (reverse scan) are shown in Figures 1 and Table 2:

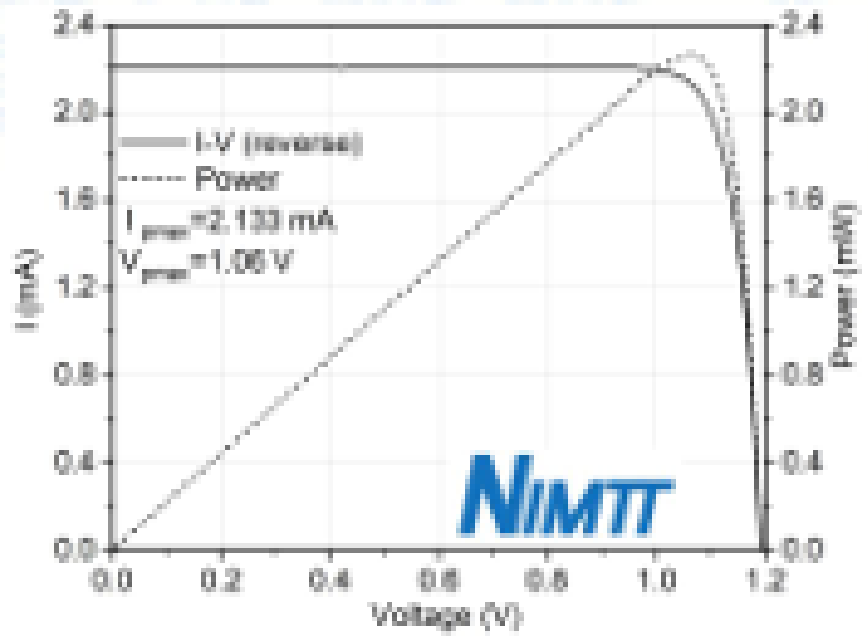


Figure 1 I-V Curve (Reverse Scan)

Table 2 I-V Parameter

| Scan Mode | $I_{sc}$ (mA) | $V_{oc}$ (V) | FF (%) | $P_{max}$ (mW) | $V_{pmax}$ (V) | $I_{pmax}$ (mA) | $\eta$ (%) |
|---|---|---|---|---|---|---|---|
| Reverse scan | 2.202 | 1.192 | 86.12 | 2.260 | 1.06 | 2.133 | 27.22 |

## Results of Test

Current-voltage (I-V) curves (forward scan) are shown in Figures 2 and Table 3:

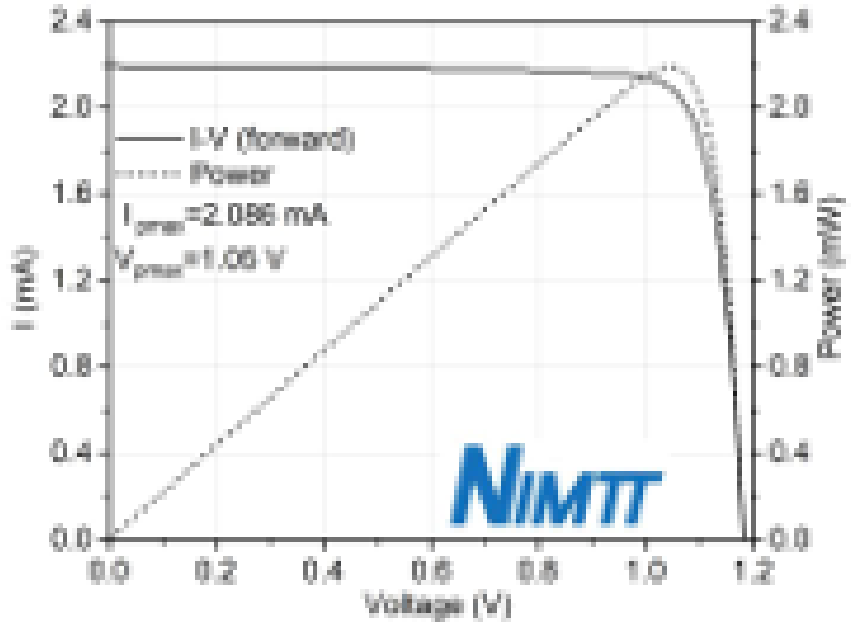


Figure 2 I-V Curve (Forward Scan)

Table 3 I-V Parameter

| Scan Mode | $I_{sc}$ (mA) | $V_{oc}$ (V) | FF (%) | $P_{max}$ (mW) | $V_{pmax}$ (V) | $I_{pmax}$ (mA) | η (%) |
|---|---|---|---|---|---|---|---|
| Forward scan | 2.196 | 1.184 | 84.25 | 2.190 | 1.05 | 2.086 | 26.38 |

备注 Note
1. Reported performance parameters take from one test value.
2. The solar cell area data is determined according to the mask area.



**Supplementary Figure 22** | Certified photovoltaic performance of the DMPA-based perovskite solar cell measured at the National Institute of Measurement and Testing Technology (NIMTT), an accredited photovoltaic certification laboratory.
