# Supplementary material for: Self-Assembled Multilayers Reduce Interfacial Energy Loss in Perovskite Solar Cells
Source: Nat Commun. 2026 Aug 8;17:9993. doi: 10.1038/s41467-026-76497-1 (PMC13590575; doi:10.1038/s41467-026-76497-1)
Supplement: Supplementary file 2 [file 41467_2026_76497_MOESM2_ESM.pdf]

nature research

Corresponding author(s): Kuan Sun

☑ Initial submission ☐ Revised version ☐ Final submission

Solar Cells Reporting Summary

Nature Portfolio wishes to improve the reproducibility of the work that we publish. This form is intended for publication with all accepted papers reporting the characterization of photovoltaic devices and provides structure for consistency and transparency in reporting. Some list items might not apply to an individual manuscript, but all fields must be completed for clarity.

For further information on Nature Research policies, including our data availability policy, see Authors & Referees.

Please check the following details are reported in the manuscript, and provide a brief description or explanation where applicable.

Area of the tested solar cells — ☑ Yes ☐ No

Report the area of the tested solar cells.

The effective testing area is 0.08 cm^2, which is also mentioned in the manuscript.

Method used to determine the device area — ☑ Yes ☐ No

Provide a description of the method and state where this information can be found in the text.

We defined the device area using a shadow mask, which is described in the manuscript.

2. Current-voltage characterization

Current density-voltage (J-V) plots in both forward and backward direction — ☑ Yes ☐ No

We include the J-V curves from both forward and backward directions in the manuscript.

Voltage scan conditions — ☑ Yes ☐ No

Provide a description of the measurement conditions (e.g. scan direction, speed, dwell times).

We applied both forward and backward scans at auto speed and 0 dwell time.

Test environment — ☑ Yes ☐ No

Provide a description of the test conditions (e.g. characterization temperature, atmosphere, humidity).

We performed the testings under a controlled ambient condition (~25 °C, ~30% RH).

Protocol for preconditioning of the device before its characterization — ☑ Yes ☐ No

Provide a description of the protocol.

The devices were tested immediately after cell assembly and then stored in N_2 atmosphere. The champion PCE were recorded 2 days after the cell assembly.

Stability of the J-V characteristic — ☑ Yes ☐ No

Provide a description of the method used. The stability of the J-V characteristic can be verified with time evolution of the maximum power point or with the photocurrent at maximum power point; see ref. 5 for details.

We tracked the photocurrent at maximum power point for stability measurement.

Description of the unusual behaviour observed during the characterization — ☑ Yes ☐ No

Provide a description of hysteresis or any other unusual behaviour observed during the characterization.

We only observed the hysteresis and analyzed it in the manuscript.

Related experimental data — ☑ Yes ☐ No

Provide a description of the related experimental data.

We have included the related data in the manuscript.

External quantum efficiency (EQE) or incident photons to current efficiency (IPCE) — ☑ Yes ☐ No

Provide a description of the technique used.

We use an integrated system (QER3018) from EnliTech to acquire EQE data.

A comparison between the integrated response under the standard reference spectrum and the response measure under the simulator — ☑ Yes ☐ No

The measured J_{SC} is 26.58 mA cm^{-2}; and integrated J_{SC} is 25.8 mA cm^{-2}.

For tandem solar cells, the bias illumination and bias voltage used for each subcell

☐ Yes ☑ No

Provide a description of the measurement conditions.

This work focuses only on single-junction perovskite solar cells.

5. Calibration

Light source and reference cell or sensor used for the characterization

☑ Yes ☐ No

Provide a description of the light source and reference cell or sensor.

We use an Xenon lamp (Newport 6279 NS). Light intensity was adjusted to AM 1.5G one sun (100 mW/cm²) with a NIM calibrated standard Si solar cell.

Confirmation that the reference cell was calibrated and certified

☑ Yes ☐ No

Identify the independent certification laboratory.

The reference Si solar cell was calibrated and certified by NIM.

Calculation of spectral mismatch between the reference cell and the devices under test

☐ Yes ☑ No

Provide a value of the spectral mismatch and/or a description of how it has been taken into account in the measurements.

6. Mask/aperture

Size of the mask/aperture used during testing

☑ Yes ☐ No

Report the size of the mask/aperature.

The mask area is 0.08 cm².

Variation of the measured short-circuit current density with the mask/aperture area

☐ Yes ☑ No

Report the difference in the short-circuit current density values measured with the mask and aperture area.

We always use a mask when recording the JV curves.

7. Performance certification

Identity of the independent certification laboratory that confirmed the photovoltaic performance

☑ Yes ☐ No

Identify the independent certification laboratory.

Certified results from an accredited photovoltaic certification laboratory, National Institute of Measurement and Testing Technology (NIMTT)

A copy of any certificate(s)

☑ Yes ☐ No

Certificate copies should be provided in the Supplementary information. Please state the supplementary item number.

Certified results show in supplementary 22

8. Statistics

Number of solar cells tested

☑ Yes ☐ No

Report how many solar cells have been tested, specifying the number of individual substrates.

We characterized each sample with a statistical set of thirty individual solar cells.

Statistical analysis of the device performance

☑ Yes ☐ No

We conducted a statistical analysis of the device performance.

9. Long-term stability analysis

Type of analysis, bias conditions and environmental conditions

☑ Yes ☐ No

Provide a description of the type of analysis, bias conditions and environmental conditions (e.g. illumination type, temperature, atmosphere humidity, encapsulation method, preconditioning temperature, bias) for each long-term stability analysis carried out; see ref. 7 and 8 for details.

We performed the stability analysis in two ways: 1. unencapsulated cells stored in N_2 atmosphere and tested in air under AM1.5G 1 Sun from time to time; 2. encapsulated cells were examined under continuous one sun equivalent white-light LED illumination at 65 °C in an N_2 atmosphere with maximum power point tracking (MPPT).

1. Shrotriya, V. *et al*. Accurate measurement and characterization of organic solar cells. *Adv. Funct. Mater.* **16**, 2016–2023 (2006).
2. Dennler, G. *et al*. The value of values. *Mat. Today* **10**, 56 (2007).
3. Cravino, A., Schilinsky, P. & Brabec, C. J. Characterization of organic solar cells: the importance of device layout. *Adv. Funct. Mater.* **17**, 3906–3910 (2007).
4. Reese, M. O. *et al*. Consensus stability testing protocols for organic photovoltaic materials and devices. *Sol. Energ. Mat. Sol. C* **95**, 1253–1267 (2011).
5. Snaith H. .J. The perils of solar cell efficiency measurements. *Nat. Photon*. **6**, 337–340 (2012).
6. Luber, E. J. & Buriak, J. M. Reporting performance in organic photovoltaic devices. *ACS Nano* **7**, 4708–4714 (2013).
7. Snaith, H. J. *et al*. Anomalous hysteresis in perovskite solar cells. *J. Phys. Chem. Lett.* **5**, 1511–1515 (2014).
8. Grätzel M. The light and shade of perovskite solar cells. *Nat. Mat*. **13**, 838–842 (2014).
9. Zimmermann E. *et al*. Erroneous efficiency reports harm organic solar cell research. *Nat. Photon*. **8**, 669–672 (2014).
10. Beard M.C., Luther J.M. & Nozik A.J. The promise and challenge of nanostructured solar cells. *Nat. Nanotech*. **9**, 951–954 (2014).
11. Timmreck, R. *et al*. Characterization of tandem organic solar cells. *Nat. Photon.* **9**, 478–479 (2015).

A number of international committees develop industry standards on the characterization of photovoltaic technologies (for example ASTM-E44 and IEC-TC 82), which can provide guidance for academic research.

This checklist template is licensed under a Creative Commons Attribution 4.0 International License, which permits use, sharing, adaptation, distribution and reproduction in any medium or format, as long as you give appropriate credit to the original author(s) and the source, provide a link to the Creative Commons license, and indicate if changes were made. The images or other third party material in this article are included in the article's Creative Commons license, unless indicated otherwise in a credit line to the material. If material is not included in the article's Creative Commons license and your intended use is not permitted by statutory regulation or exceeds the permitted use, you will need to obtain permission directly from the copyright holder. To view a copy of this license, visit http://creativecommons.org/licenses/by/4.0/
