# Supplementary material for: Self-Assembled Multilayers Reduce Interfacial Energy Loss in Perovskite Solar Cells
Source: Nat Commun. 2026 Aug 8;17:9993. doi: 10.1038/s41467-026-76497-1 (PMC13590575; doi:10.1038/s41467-026-76497-1)
Supplement: Supplementary file 3 [file 41467_2026_76497_MOESM3_ESM.pdf]

nature portfolio

Peer Review File

Self-Assembled Multilayers Reduce Interfacial Energy Loss in Perovskite Solar Cells

Corresponding Author: Professor Kuan Sun

This file contains all reviewer reports in order by version, followed by all author rebuttals in order by version.

Version 0:

Reviewer comments:

Reviewer #1

(Remarks to the Author)
Pan and coauthors developed two self-assembled monolayer (SAM) molecules, DMPA and BCPA. DMPA is proposed to promote an ordered and densely packed monolayer through enhanced intermolecular π–π interactions, while the methoxy substituent is suggested to interact with the perovskite layer to passivate defects and guide crystallization, resulting in a reported PCE of 27.59% and good photothermal stability. However, the experimental results are insufficient to convincingly support the proposed mechanism by which DMPA influences device performance. Therefore, in its present form, the work is not recommended for publication in the high-impact journal Nature Communications. The detailed comments are provided below.

1. The authors state that "the extra methoxy group in DMPA not only reinforces intermolecular π–π stacking, leading to a denser and more ordered assembly, but also chemically bonds with the perovskite layer, passivating defects at the buried interface and optimizing perovskite crystallization.". Defect passivation at the buried interface is a key claimed advantage of DMPA; therefore, additional experimental or theoretical evidence is required to elucidate the interaction between the methoxy group and the perovskite layer.

2. In Figures 1m–o, the 2PACz and BCPA layers appear to exhibit a bilayer-like structure, whereas the DMPA layer appears to form a monolayer. However, previous reports suggest that 2PACz does not form a bilayer structure (Nature 2023, 624, 289). Can the authors provide additional experimental evidence to support the proposed SAM layer structures of 2PACz and DMPA? In particular, evidence supporting the proposed "hand-in-hand configuration" of the DMPA layer is required.

3. In Figure 2a, what molecular species do the signals at 1040 cm-1 and 1042 cm-1 correspond to? For example, 954 cm−1 and 1036 cm−1 are attributed to P–OH functional groups and a band near 1068 cm-1 is assigned to P=O.

4. For the conductivity and mobility measurements using the ITO/NiO_x/SAM/Ag structure in Supplementary Fig. 10 and 11, the electrical properties of the ITO layer may influence the measured values, given the relative thicknesses of the SAM, NiO_x, and ITO layers. Please provide additional analysis to these results. Moreover, the energy-level alignment in ITO/NiO_x/SAM/Ag may differ from that in ITO/NiO_x/SAM/perovskite; therefore, characterization of the Ag Fermi-level is recommended to support the energy-level alignment analysis for the ITO/NiO_x/SAM/Ag.

5. Because poor wettability would be caused by the mismatch between nonpolar SAM head groups and polar perovskite precursor solutions, the denser and more uniform DMPA layer would be anticipated to exhibit reduced wettability. However, in Figures 3a–c, the DMPA layer exhibits the smallest contact angle. Please provide further discussion to rationalize this result.

6. In Figures 4d and 4e, the ITO/NiO_x/DMPA/perovskite stack exhibits the lowest PL intensity and the shortest carrier lifetime, which are attributed to efficient carrier extraction. PL intensity is generally correlated with photoluminescence quantum yield (PLQY), which is commonly used to estimate the quasi-Fermi-level splitting (QFLS). It would be helpful to clarify whether the ITO/NiO_x/DMPA/perovskite sample exhibits the highest QFLS among the studied samples. Given the proposed defect passivation arising from interactions between DMPA and the perovskite, one would expect this device to exhibit the largest QFLS.

7. In Supplementary Fig. 16a, in situ UV–vis absorption spectra data are not provided.

8. In Fig. 4j-l, please provide the decay curves of both GSB and free-carrier signals. The GSB signal reflects optical excitation but does not directly represent free-carrier dynamics, such as carrier generation, recombination, or extraction.

9. A certified PCE value is recommended to support the reported PCE of 27.59%.

Reviewer #2

(Remarks to the Author)
This manuscript addresses a critical challenge in perovskite solar cell (PSC) research for optimizing hole transport layers (HTLs) to mitigate aggregation, poor wettability, and weak interfacial interactions in self-assembled monolayers (SAMs) and presents a solution through rational molecular design, which demonstrates advancements in both device efficiency and stability. However, I need to reject the submission based on following adjustment:
1. Novelty of Material Design
From the standpoint of material design, the present work appears to assemble several previously reported SAM architectures, which diminishes the overall novelty. Moreover, concerning the authors' proposed novel molecular self-assembly model termed "hang-in-hand," reliance solely on elementary molecular dynamics simulations is insufficient to substantiate the physical existence of this configuration. And according to the figure 1m-o, it is hard to identify the "more ordered" structure of DMPA compared with the control SAM and BCPA. And for such kind of molecular packing on NiOx could lead to passivation effect of PA group for control SAM and BCPA. More detailed characterizations should be provided to determine the packing model of SAM deposited on NiOx, as well as the thin film thickness, which plays vital role in device performance.
2. Efficiency Claims and Third-Party Validation
The power conversion efficiency reported herein ranks among the highest values achieved to date for perovskite photovoltaic devices employing SAM/NiOx hole-transport layers. However, for a manuscript submitted to a high-impact journal such as Nature Communications, efficiency values must be independently verified by an accredited third-party institution; in the absence of such certification, the reported performance data remain unconvincing.
3. Consistency and Uniformity of Device Performance
The study lacks fundamental investigations into batch-to-batch consistency and spatial uniformity of device efficiency, essential benchmarks for evaluating reproducibility. Consequently, I harbor reservations regarding the reliability of the stated device performance metrics.
4. Scalability to Large-Area Devices
Researchers active in perovskite photovoltaics are well aware that the field has advanced to large-area device and module fabrication. The omission of any demonstration of large-area devices based on this ostensibly high-performance material casts doubt on the scalability of its advantageous properties.
5. Stability Testing Under Thermal Stress
With respect to stability assessment, a device employing only BCP as the electron-blocking layer is unlikely to maintain stable operation under prolonged thermal stress at 65 °C. Therefore, the presented stability data warrant careful scrutiny and may not be fully credible.
6. High-Resolution Mass Spectrometry Reporting
The high-resolution mass spectrometry entry stating "HRMS-ESI m/z Calcd for $C_{18}H_{16}NO_3P$ $[M+H]^+$: 326.09, found 326.09" contains a serious error and does not reflect acceptable reporting standards.
7. Anomalies in J–V Scan Directions
Analysis of the reported J–V characteristics reveals an inconsistency: for the control and BCPA devices, all reverse-scan parameters (VOC, JSC, and fill factor) exceed their forward-scan counterparts, whereas for the DMPA device the reverse-scan VOC and JSC are lower than in the forward scan. This discrepancy merits explanation, as it suggests potential measurement artefacts or inconsistencies in device behavior.
8. Some minor error
In page 8, the mention of "DCPA" should be wrong.
In Page 10, The description of "The surface molecular density of NiOx/DMPA (6.8 × 1013 molecules cm-2) was higher than those of NiOx/Control SAM (5.0 × 1013 molecules cm-2) and NiOx/BCPA (6.8× 1013 molecules cm-2)" is wrong.

Reviewer #3

(Remarks to the Author)
This study reports a strategy to enhance the efficiency and stability of inverted perovskite solar cells by designing a DMPA SAM molecule. The authors propose that DMPA adopts a unique "hand-in-hand" molecular configuration, leading to improved interfacial contact, reduced residual stress, and a high PCE of 27.59%. While the findings are interesting and the performance are impressive, the manuscript requires solid experimental evidence to substantiate the proposed molecular configuration. Additionally, independent certification of the efficiency and further clarification on the stress relief mechanism are needed to support the conclusions.

1. A primary concern is that the authors claim that DMPA adopts a "hand-in-hand" configuration while the Control and BCPA adopt a vertical stacking mode. While the simulation is supportive, it is not experimental evidence. The authors should provide direct experimental evidence of this specific configuration. Without experimental corroboration, the "hand-in-hand" mechanism remains a theoretical hypothesis.

2. A lower initial GSB amplitude could imply less absorption or immediate quenching, but "faster transfer" is usually derived from the decay kinetics (how fast the signal fades), not just the intensity. A normalized decay kinetic plot of the GSB peak should be provided to compare the hole extraction rates.

3. The GIXRD data show a significant reduction in residual stress for DMPA-based films. The mechanism needs more explanation. How does a monolayer at the bottom interface relieve stress throughout the bulk of a thick perovskite film? Expanding on the relationship between the "hand-in-hand" assembly and the relaxation of lattice strain would strengthen the quality and impact of this study.

4. The authors report a very high PCE of 27.59%. For efficiency claims exceeding 27.5% in a high-impact journal, independent certification from an accredited testing laboratory is standard practice and highly recommended.

5. Please provide SEM images with a larger field of view (e.g., 10 μm × 10 μm) to better evaluate the overall uniformity and quality of the perovskite films prepared on different SAMs.

6. What is the specific mechanism responsible for the noticeable EQE enhancement in the 400–650 nm range for the DMPA-based device?

7. Please specify the initial PCE values for each device used in the MPPT tests to provide a clear baseline for stability comparison.

8. Additionally, please ensure that the x- or y-axis span of figs. 2g-2i are identical across all samples to enable a fair visual comparison of the potential distributions.

Version 1:

Reviewer comments:

Reviewer #1

(Remarks to the Author)
The authors have addressed all my concerns, and the manuscript is now suitable for publication in Nature Communications.

Reviewer #2

(Remarks to the Author)
While the authors have addressed the aforementioned comments with revisions and responses, I reject this manuscript for publication in top-tier journal of Nature Communications. The revised manuscript has added four authors, notably including the corresponding author "Wei Wei"; however, the "Author Contributions" section fails to document any contribution from Wei Wei. This constitutes a serious ethical breach in academia."

Reviewer #3

(Remarks to the Author)
The authors have addressed my main concerns in the revised manuscript. The key mechanistic claims have been revised in a more cautious and evidence-based manner, and the additional analyses and clarifications have improved the rigor and clarity of this study. Overall, I consider the revised manuscript suitable for publication after the following minor corrections.
1. Please check the fitting time constants in Supplementary Fig. 20/Fig. R18, as the values in the legend appear inconsistent with the decay curves.
2. Please correct the caption of Fig. 3, where the SEM images should be referred to as Fig. 3d-f rather than Fig. 3d-e.
3. Please update the SI statement "Supplementary Figs. 1 to 17" to match the current number of supplementary figures (22 figures now).

Open Access This Peer Review File is licensed under a Creative Commons Attribution 4.0 International License, which permits use, sharing, adaptation, distribution and reproduction in any medium or format, as long as you give appropriate credit to the original author(s) and the source, provide a link to the Creative Commons license, and indicate if changes were made.
In cases where reviewers are anonymous, credit should be given to 'Anonymous Referee' and the source.
The images or other third party material in this Peer Review File are included in the article's Creative Commons license, unless indicated otherwise in a credit line to the material. If material is not included in the article's Creative Commons license and your intended use is not permitted by statutory regulation or exceeds the permitted use, you will need to obtain permission directly from the copyright holder.
To view a copy of this license, visit https://creativecommons.org/licenses/by/4.0/

Response Letter

Dear Reviewers,

We sincerely thank you for your evaluation of our manuscript (Manuscript ID: NCOMMS-25-100225-T) and for the constructive comments and valuable suggestions. These insights have been instrumental in improving the quality and clarity of our work.

We have carefully addressed all comments and revised the manuscript accordingly. Below, we provide a point-by-point response to each comment. Reviewer comments are presented in **black**, our responses are in **blue**, and the corresponding revisions in the manuscript are highlighted in **red**.

In this revision, we have also updated the manuscript to ensure consistency in the numbering and citation of all figures and tables following the inclusion of additional data and references.

We hope that the revised manuscript meets your expectations. Should you require any further clarification, please do not hesitate to contact us.

Thank you for your consideration.

Sincerely,

Kuan Sun,

School of Energy & Power Engineering, Chongqing University

Tel: +86 13996260014

Email: kuan.sun@cqu.edu.cn

Group website: https://www.x-mol.com/groups/LaFREMD

ResearchGate: https://www.researchgate.net/profile/Kuan_Sun

Reviewer #1 (Remarks to the Author):

Pan and coauthors developed two self-assembled monolayer (SAM) molecules, DMPA and BCPA. DMPA is proposed to promote an ordered and densely packed monolayer through enhanced intermolecular π–π interactions, while the methoxy substituent is suggested to interact with the perovskite layer to passivate defects and guide crystallization, resulting in a reported PCE of 27.59% and good photothermal stability. However, the experimental results are insufficient to convincingly support the proposed mechanism by which DMPA influences device performance. Therefore, in its present form, the work is not recommended for publication in the high-impact journal Nature Communications. The detailed comments are provided below.

Our response: We sincerely thank the reviewer for the thorough evaluation and constructive feedback. We appreciate the acknowledgment of the high device performance and stability achieved in this study. We also acknowledge and understand the reviewer's concern regarding the mechanistic evidence for the role of DMPA.

In response, we have substantially strengthened the experimental support and discussion in the revised manuscript to more clearly establish the mechanisms by which DMPA contributes to device performance. Specifically, we have added new data and expanded the analysis related to DMPA's role in enhancing intermolecular packing, passivating perovskite defects, and regulating crystallization, as detailed in the point-by-point responses below.

We believe that these revisions now provide a solid and convincing foundation for the proposed mechanism. Thank you again for the valuable comments, which have greatly improved the quality of our manuscript.

1. The authors state that "the extra methoxy group in DMPA not only reinforces intermolecular π–π stacking, leading to a denser and more ordered assembly, but also chemically bonds with the perovskite layer, passivating defects at the buried interface and optimizing perovskite crystallization." Defect passivation at the buried interface is a key claimed advantage of DMPA; therefore, additional experimental or theoretical evidence is required to elucidate the interaction between the methoxy group and the

perovskite layer.

Our response: We thank the reviewer for this important comment regarding the need for mechanistic evidence of the methoxy–perovskite interaction. In response, we have strengthened the supporting evidence from three complementary perspectives: (a) literature precedent, (b) theoretical calculations, and (c) in-situ crystallization analysis.

(a) Literature precedent. Recent studies have shown that methoxy-functionalized aromatic molecules can interact effectively with perovskite surfaces and contribute to defect passivation. For example, a report (*J. Am. Chem. Soc.* **2025**, *147*, 8004–8011) demonstrated that "incorporating methoxy groups onto the benzene ring at different positions (ortho, meta, and para) … not only increases anchoring points with the perovskite layer but also fine-tunes the molecular dipole moment." This supports our design rationale: the methoxy group in DMPA is expected to provide additional coordination sites for undercoordinated Pb^{2+} at the perovskite surface, thereby enhancing interfacial binding and passivating defects.

(b) Theoretical calculations. To directly evaluate the interaction strength, we performed DFT calculations of the adsorption energy (E_{ad}) of the SAM molecules on the perovskite surface. DMPA exhibits the most negative E_{ad} (−0.39 eV), compared to the control (−0.28 eV) and BCPA (−0.32 eV), confirming its stronger interfacial affinity.

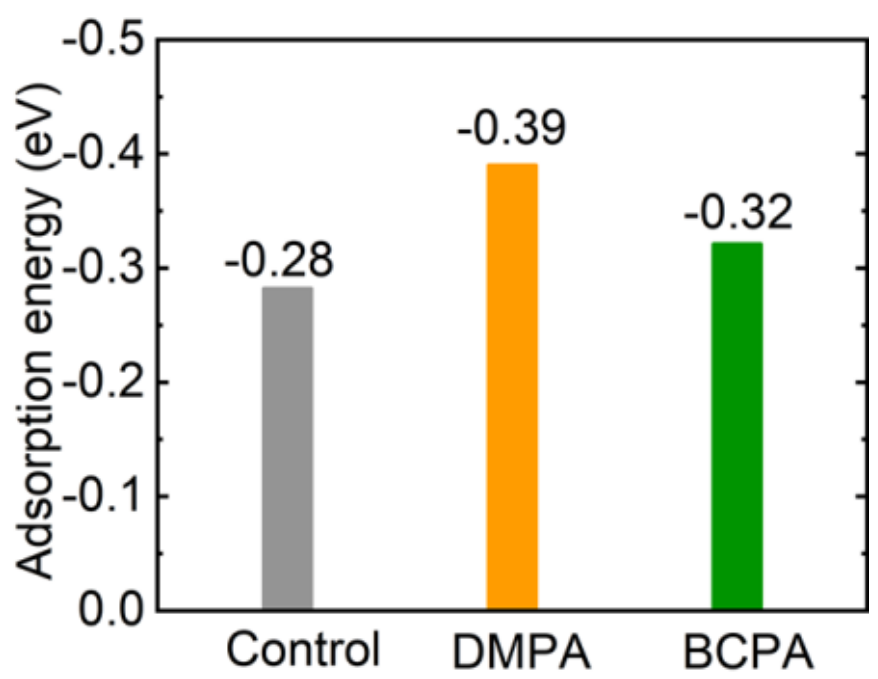

Fig. R1 Adsorption energy (E_{ad}) of different SAM molecules on the perovskite surface, obtained from DFT calculations.

Furthermore, the calculations reveal that the methoxy oxygen atoms in DMPA can form dual O···Pb bonds with undercoordinated Pb^{2+} at the interface. This chelation reduces local octahedral distortion and increases the defect formation energy (from

1.22–1.31 eV for the BCPA/control to 2.05 eV for DMPA), indicating a substantial suppression of defect formation.

These results have been incorporated into the main text as follows: "Additionally, we utilized DFT to determine E_{ad} of these SAM molecules on PVK. Notably, compared to E_{ad} of control (-0.28 eV) and BCPA (-0.32 eV), the E_{ad} of DMPA (-0.39 eV) with perovskite is the most negative (Supplementary Fig. R1), signifying that DMPA possesses the strongest affinity among the three SAMs."

(c) In-situ GIWAXS analysis. To further elucidate the role of methoxy groups in regulating perovskite growth, we performed in-situ GIWAXS measurements during spin-coating and annealing processes (Fig. R2). Time-resolved GIWAXS maps show that DMPA prolongs the solvent-phase duration before the appearance of crystalline diffraction peaks, indicating retarded crystallization kinetics. Such delayed nucleation is known to favor the growth of larger, more ordered domains, consistent with the observed reduction in defect density and improved device performance.

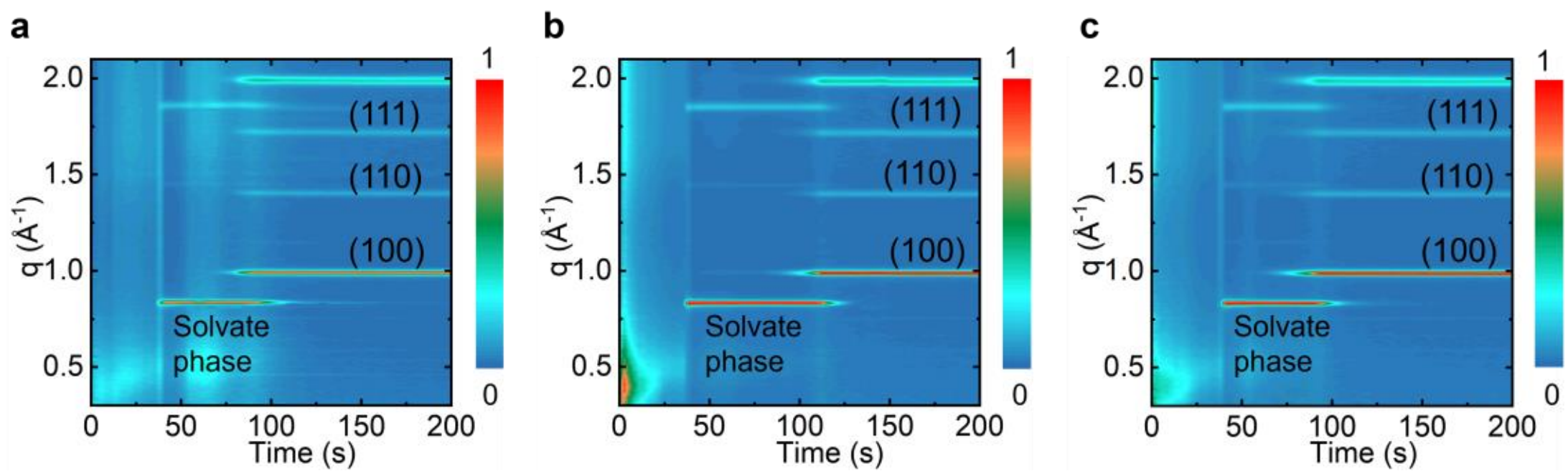

Fig. R2. In-situ GIWAXS false-color maps showing the diffraction intensity as a function of scattering vector q ($Å^{-1}$) and time (s) for (a) control, (b) DMPA-modified, and (c) BCPA-modified perovskite films. The data track the structural evolution during spin-coating and annealing, with the characteristic peaks at $q \approx 1.00$, 1.42, and 1.73 $Å^{-1}$ corresponding to the (100), (110), and (111) perovskite planes, respectively.

The corresponding descriptions are provided below and have been updated in the main text. "To uncover the underlying mechanism for crystal growth, we further conducted in-situ GIWAXS measurement under the programmed spin-coat and annealing procedure. The corresponding false-color maps of the control, DMPA, and

BCPA of the diffraction intensity versus scattering vector q (in units per angstrom) and time (in seconds) are shown in Supplementary Fig. 16a-c. At the moment of antisolvent dripping, a solvent phase emerged. Subsequently, during the annealing process, characteristic diffraction peaks were observed, at q = 1.00, 1.42, and 1.73 $Å^{-1}$, corresponding to the (100), (110), and (111) facets of the perovskite, respectively. Compared to the Control and BCPA, DMPA exhibits a prolonged duration of the solvent phase, suggesting that the SAM molecule modulates perovskite growth. In other words, DMPA retards the crystallization kinetics of the perovskite, which can enhance perovskite crystallization and growth."

Taken together, these results provide a multi-faceted and coherent picture: the methoxy group in DMPA (i) strengthens SAM/perovskite binding via O···Pb coordination, (ii) raises the energy barrier for defect formation, and (iii) modulates crystallization kinetics to yield higher-quality films.

2. In Figures 1m–o, the 2PACz and BCPA layers appear to exhibit a bilayer-like structure, whereas the DMPA layer appears to form a monolayer. However, previous reports suggest that 2PACz does not form a bilayer structure (Nature 2023, 624, 289). Can the authors provide additional experimental evidence to support the proposed SAM layer structures of 2PACz and DMPA? In particular, evidence supporting the proposed "hand-in-hand configuration" of the DMPA layer is required.

Our response: We sincerely thank the reviewer for this insightful comment regarding the structural interpretation of the SAM layers. We fully agree that assigning a bilayer-like structure to 2PACz is not consistent with previous reports (Nature 2023, 624, 289). In light of this, we have carefully re-evaluated our interpretation and revised the manuscript to avoid overinterpretation of the experimental images.

We have removed all descriptions related to the "hand-in-hand" configuration of DMPA, as we acknowledge that the current evidence, including molecular dynamics (MD) simulations, is insufficient to experimentally validate a specific molecular packing model.

To provide a more reliable and evidence-based interpretation, we focus on the

relative aggregation behavior of the SAMs. MD simulations (Fig. R3) indicate that DMPA molecules exhibit reduced intermolecular aggregation and more uniform surface distribution compared to BCPA. In contrast, BCPA shows a stronger tendency toward molecular stacking, which may lead to increased apparent thickness (~1.6 nm) and aggregation features. This interpretation is consistent with literature reports (*Nature Photonics* **2026**, *20*, 232–240; *Nature Communications* **2026**, *17*, 1472; *Nature Photonics* **2026**, *20*, 287-295).

The conclusions of this work do not rely on a specific molecular configuration, but are supported by consistent experimental observations, including improved surface uniformity, enhanced wettability, reduced defect density, and improved device performance.

We have revised the manuscript accordingly to present a more cautious and experimentally supported interpretation. The corresponding descriptions are provided below and have been updated in the main text."Molecular dynamics (MD) simulations visualized the top and side views of Control, BCPA, and DMPA on the NiO_x substrate (Fig. R3). The results show that DMPA SAMs effectively suppress their own aggregation and improve substrate coverage. This can be attributed to the optimal π-π interactions in DMPA, which enhance surface coverage, while the rotating phenyl ring introduces steric hindrance that effectively inhibits molecular stacking. In contrast, BCPA displays a thickness of approximately 1.6 nm, likely due to its greater tendency for molecular aggregation (Supplementary Fig. 7)."

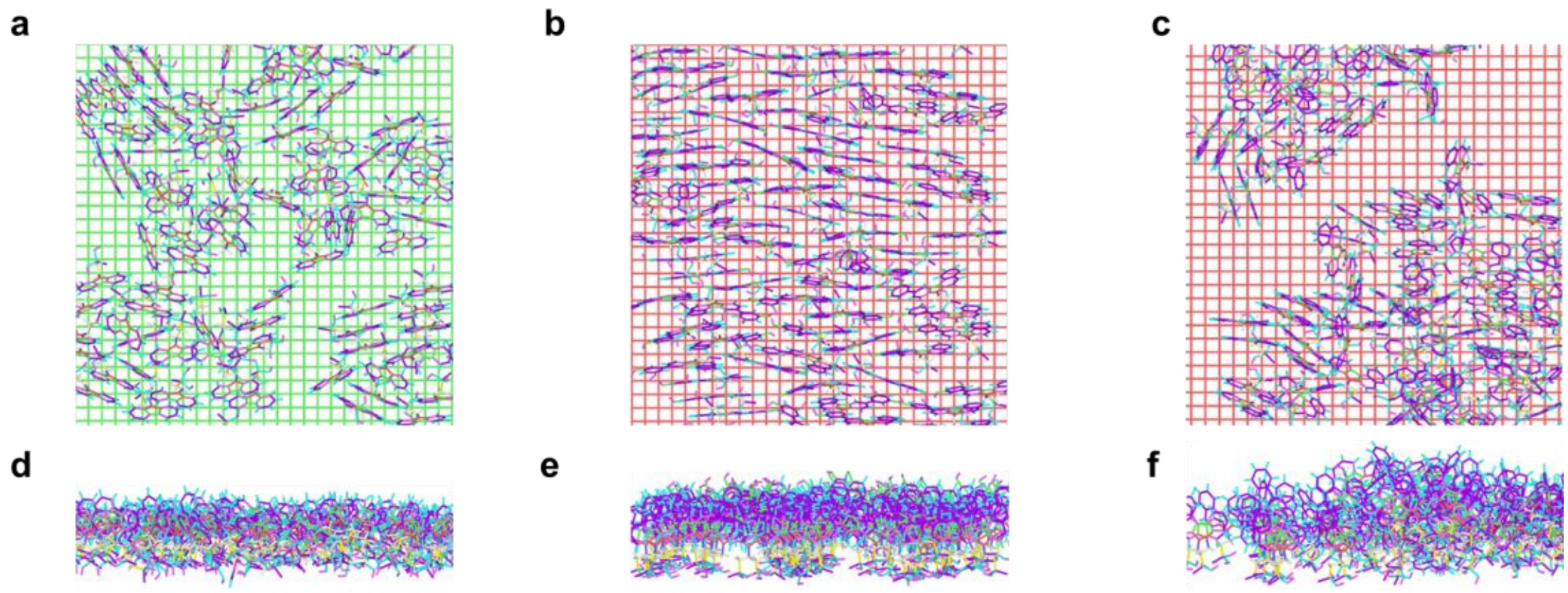

Fig. R3 **a - c** Top-view and **d - f** side-view molecular dynamics snapshots of the SAM

configurations on NiO_x, illustrating differences in packing order, molecular orientation, and surface coverage.

3. In Figure 2a, what molecular species do the signals at 1040 cm^{-1} and 1042 cm^{-1} correspond to? For example, 954 cm^{-1} and 1036 cm^{-1} are attributed to P–OH functional groups and a band near 1068 cm^{-1} is assigned to P=O.

Our response: We thank the reviewer for this helpful question regarding the assignment of the FT-IR peaks. The bands observed at 1040–1042 cm^{-1} are attributed to the stretching vibration of the P–OH group, which is consistent with previous reports (*Science* **2023**, *382*, 284–289; *Joule* **2025**, *3*, 101815). Based on this assignment, we used the absorption band at 1042 cm^{-1} for FT-IR mapping to evaluate the spatial homogeneity of the SAM films. The corresponding description has been clarified in the revised manuscript. "FT-IR mapping was performed to evaluate the spatial homogeneity of the SAMs. The mapping was based on the absorption band at approximately 1042 cm^{-1}, which corresponds to the stretching vibration of the P–OH group."

4. For the conductivity and mobility measurements using the ITO/NiO_x/SAM/Ag structure in Supplementary Fig. 10 and 11, the electrical properties of the ITO layer may influence the measured values, given the relative thicknesses of the SAM, NiO_x, and ITO layers. Please provide additional analysis to these results. Moreover, the energy-level alignment in ITO/NiO_x/SAM/Ag may differ from that in ITO/NiO_x/SAM/perovskite; therefore, characterization of the Ag Fermi-level is recommended to support the energy-level alignment analysis for the ITO/NiO_x/SAM/Ag.

Our response: We thank the reviewer for this insightful comment regarding the potential influence of the ITO layer and the energy-level alignment in the ITO/NiO_x/SAM/Ag structure.

We agree that the relative thicknesses of ITO, NiO_x, and SAM layers may affect the measured electrical properties. To evaluate this effect, we designed a series of

control structures, including ITO/Ag, ITO/NiO_x/Ag, and ITO/NiO_x/SAM/Ag (with different SAMs). As shown in Figure R4, the current–voltage characteristics for each structure exhibit good reproducibility, and the differences between devices primarily arise upon introducing different SAM layers.

In our measurements, Ag serves as a metallic top electrode providing an ohmic contact, as evidenced by the linear current–voltage characteristics passing through the origin. Under such conditions, the transport is dominated by bulk and interfacial resistance rather than injection barriers at the Ag interface. Therefore, the exact position of the Ag Fermi level does not play a determining role in the analysis of relative conductivity and mobility trends in this configuration. We hope this explanation adequately addresses the reviewer's concerns.

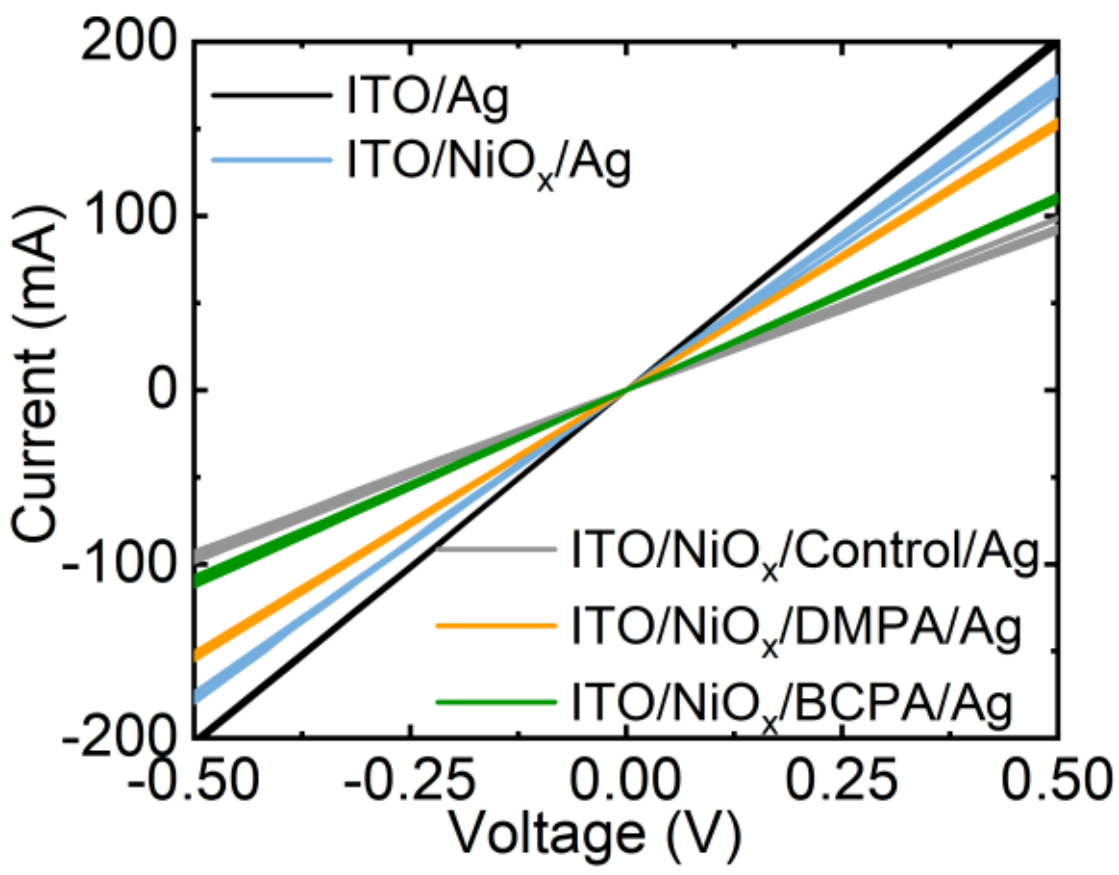

Fig. R4. The conductivity of different samples, with five curves per sample.

5. Because poor wettability would be caused by the mismatch between nonpolar SAM head groups and polar perovskite precursor solutions, the denser and more uniform DMPA layer would be anticipated to exhibit reduced wettability. However, in Figures 3a–c, the DMPA layer exhibits the smallest contact angle. Please provide further discussion to rationalize this result.

Our response: We thank the reviewer for this important comment regarding the seemingly counterintuitive wettability behavior. We agree that densely packed SAMs with nonpolar aromatic backbones are expected to exhibit reduced wettability toward polar perovskite precursor solutions. However, in the case of DMPA, the improved

wettability originates from the dominant contribution of methoxy-induced interfacial interactions and surface uniformity.

(a) Methoxy-induced interfacial interactions. The methoxy groups in DMPA introduce polar oxygen atoms that can interact with the precursor solution. Specifically, these groups can coordinate with Pb^{2+} species and form weak hydrogen-bonding interactions with organic cations such as FAI. These interactions enhance the affinity between the SAM surface and the polar precursor solution, facilitating liquid spreading.

(b) Improved surface uniformity. In addition, the more homogeneous and less aggregated DMPA layer provides a smoother and energetically more uniform surface, which is favorable for wetting. In contrast, aggregated SAM domains (e.g., in BCPA) can introduce local heterogeneity that hinders liquid spreading.

Such behavior is consistent with recent literature, where methoxy terminal groups have been shown to enhance the wettability of SAM-modified surfaces (*Nature Photonics* **2026**, *20*, 287-295).

Therefore, despite its dense packing, the DMPA layer exhibits improved wettability due to the combined effects of methoxy-induced polarity, interfacial interactions, and surface homogeneity, as reflected by the reduced contact angle in Figures 3a–c.

6. In Figures 4d and 4e, the ITO/NiO_x/DMPA/perovskite stack exhibits the lowest PL intensity and the shortest carrier lifetime, which are attributed to efficient carrier extraction. PL intensity is generally correlated with photoluminescence quantum yield (PLQY), which is commonly used to estimate the quasi-Fermi-level splitting (QFLS). It would be helpful to clarify whether the ITO/NiO_x/DMPA/perovskite sample exhibits the highest QFLS among the studied samples. Given the proposed defect passivation arising from interactions between DMPA and the perovskite, one would expect this device to exhibit the largest QFLS.

Our response: We thank the reviewer for this insightful comment regarding the relationship among PL intensity, carrier lifetime, and quasi-Fermi level splitting (QFLS).

(a) QFLS analysis. In response to the reviewer's suggestion, we have measured the QFLS values based on photoluminescence quantum yield (PLQY), as shown in Figure R4 (Supplementary Fig. 18). The DMPA-based sample exhibits the highest QFLS value (1.20 eV), compared to the control (1.12 eV) and BCPA (1.17 eV), which is consistent with the trend in open-circuit voltage (V_{OC}). This result confirms that DMPA effectively suppresses non-radiative recombination losses, in line with the proposed defect passivation mechanism.

(b) Distinguishing defect passivation and charge extraction. We note that the PL results in Figures 4d–e were obtained from device-relevant stacks (ITO/NiO_x/SAM/perovskite), where efficient charge extraction at the interface plays a dominant role. In this configuration, reduced PL intensity and shorter carrier lifetime for DMPA indicate faster carrier extraction rather than increased non-radiative recombination (similar with our previous work of (*Adv. Funct. Mater.* **2025**, *35*, 2420084).

To decouple these effects, we further performed PL measurements on samples without charge transport layers (glass/SAM/perovskite, Figures 4a–b). In this case, the DMPA-treated sample exhibits stronger PL intensity and longer carrier lifetime, indicating reduced trap-assisted recombination in the perovskite film.

The corresponding descriptions are provided below and have been updated in the main text. "To quantify the non-radiative recombination losses at the HTL/perovskite interface, photoluminescence quantum yield (PLQY) measurements were executed to quantify quasi-Fermi level splitting (QFLS) (Supplementary Fig. 18). The DMPA sample showed the highest QFLS value of 1.20 eV than control (1.12 eV) and BCPA (1.17 eV), which aligns well with the corresponding open-circuit voltage (V_{OC}) trend. Due to the reduction of morphological defects such as pinholes at the interface in the DMPA group, DMPA more effectively suppresses non-radiative recombination compared to the control and BCPA. The QFLS measurements also corroborate this result."

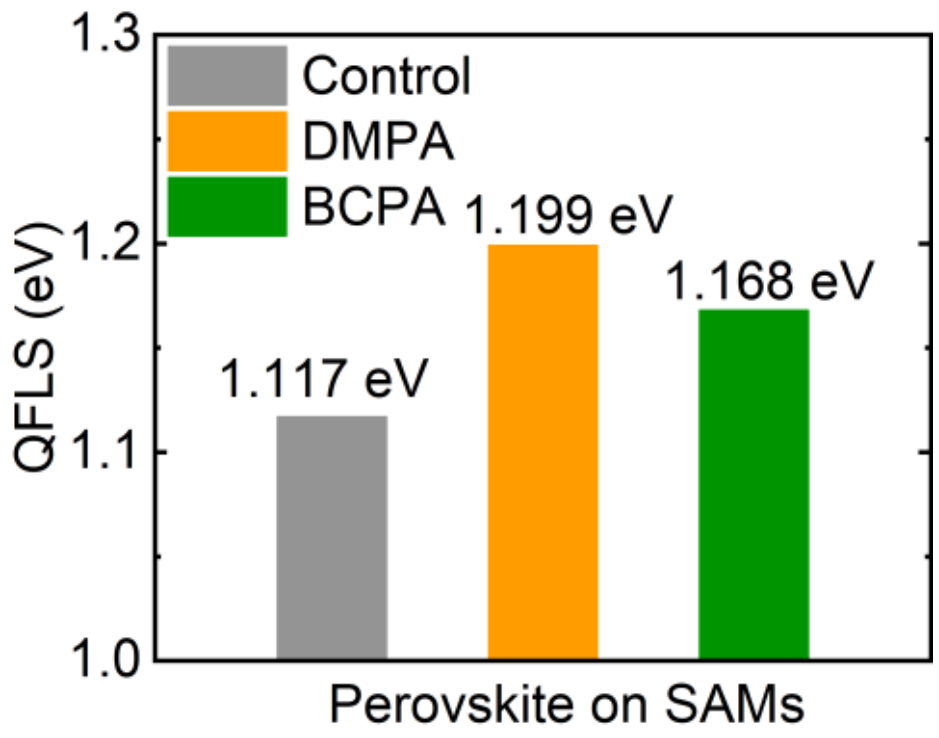

Fig. R5. QFLS of perovskite films based on SAMs

7. In Supplementary Fig. 16a, in-situ UV–vis absorption spectra data are not provided.

Our response: We thank the reviewer for pointing out this mistake. The term "in-situ" in the original manuscript was inaccurate and has been removed in the revised version to avoid any confusion. The corresponding description has been corrected in the manuscript.

8. In Fig. 4j-l, please provide the decay curves of both GSB and free-carrier signals. The GSB signal reflects optical excitation but does not directly represent free-carrier dynamics, such as carrier generation, recombination, or extraction.

Our response: We thank the reviewer for this insightful comment regarding the distinction between ground-state bleaching (GSB) and free-carrier dynamics. We agree that the GSB signal primarily reflects state filling and does not directly represent free-carrier dynamics such as carrier recombination or extraction. In the revised manuscript, we have carefully clarified this point and avoided overinterpretation of the GSB signal.

In our study, the transient absorption measurements were performed on NiO_x/SAM/perovskite stacks, where interfacial charge extraction plays a significant role in the decay dynamics. Under these conditions, the recovery of the GSB signal is influenced not only by carrier recombination but also by carrier extraction at the interface. Therefore, the faster GSB recovery observed for the DMPA-treated sample is interpreted as a relative indicator of more efficient charge extraction, rather than a direct measurement of free-carrier recombination dynamics.

We have revised the corresponding discussion in the manuscript to clearly state

this limitation and to emphasize that the transient absorption results are used for comparative analysis of interfacial charge transfer behavior.

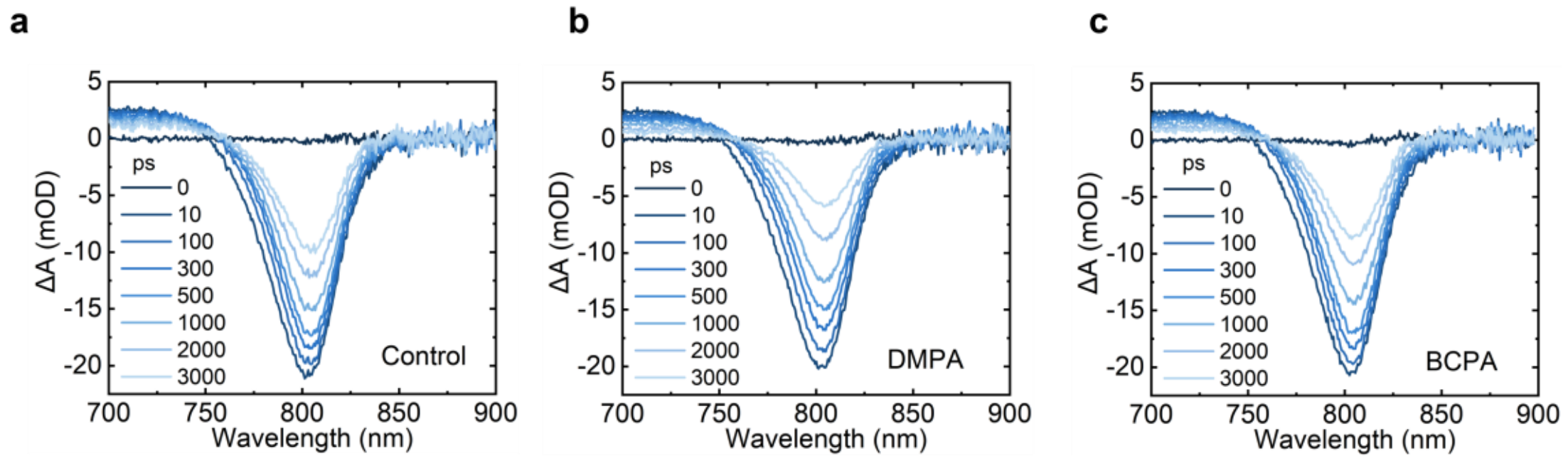

Fig. R6. Femtosecond transient absorption spectra at a selected pump－probe delay time of (a) NiO_x/control/perovskite films, (b) NiO_x/DMPA/perovskite films and (c) NiO_x/BCPA/perovskite films. ΔA, difference in optical density between the excited state and the ground state of a sample.

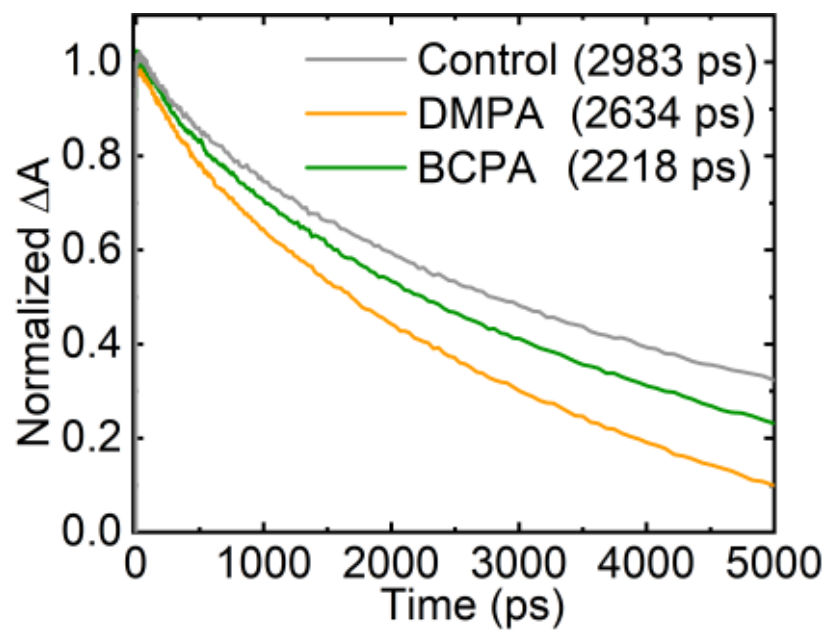

Fig. R7. Corresponding GSB decay at 803 nm of the different SAM treatments.

The corresponding descriptions are provided below and have been updated in the main text. "We further employed femtosecond transient absorption (TA) spectroscopy to investigate the interfacial charge dynamics in NiO_x/SAM/perovskite stacks (Fig. 4j–l). All samples exhibit a characteristic ground-state bleaching (GSB) signal centered at ~803 nm, corresponding to state filling of the perovskite absorber. It should be noted that the GSB signal primarily reflects the population of excited states and does not directly represent free-carrier dynamics such as recombination or transport. In device-relevant structures, the recovery of the GSB signal is governed by a combination of carrier recombination and interfacial charge extraction processes.

The DMPA sample exhibits a faster GSB recovery compared to the control and

BCPA samples (Supplementary Fig. 20). This behavior suggests more efficient interfacial hole extraction at the NiO_x/SAM/perovskite interface in the presence of DMPA. “

9. A certified PCE value is recommended to support the reported PCE of 27.59%.

Our response: We agree with the reviewer for this valuable suggestion. We have provided a third-party certification report, where the certified PCE is up to 27.2% as shown in Fig R8. The certification report has been updated in the Supporting Information.

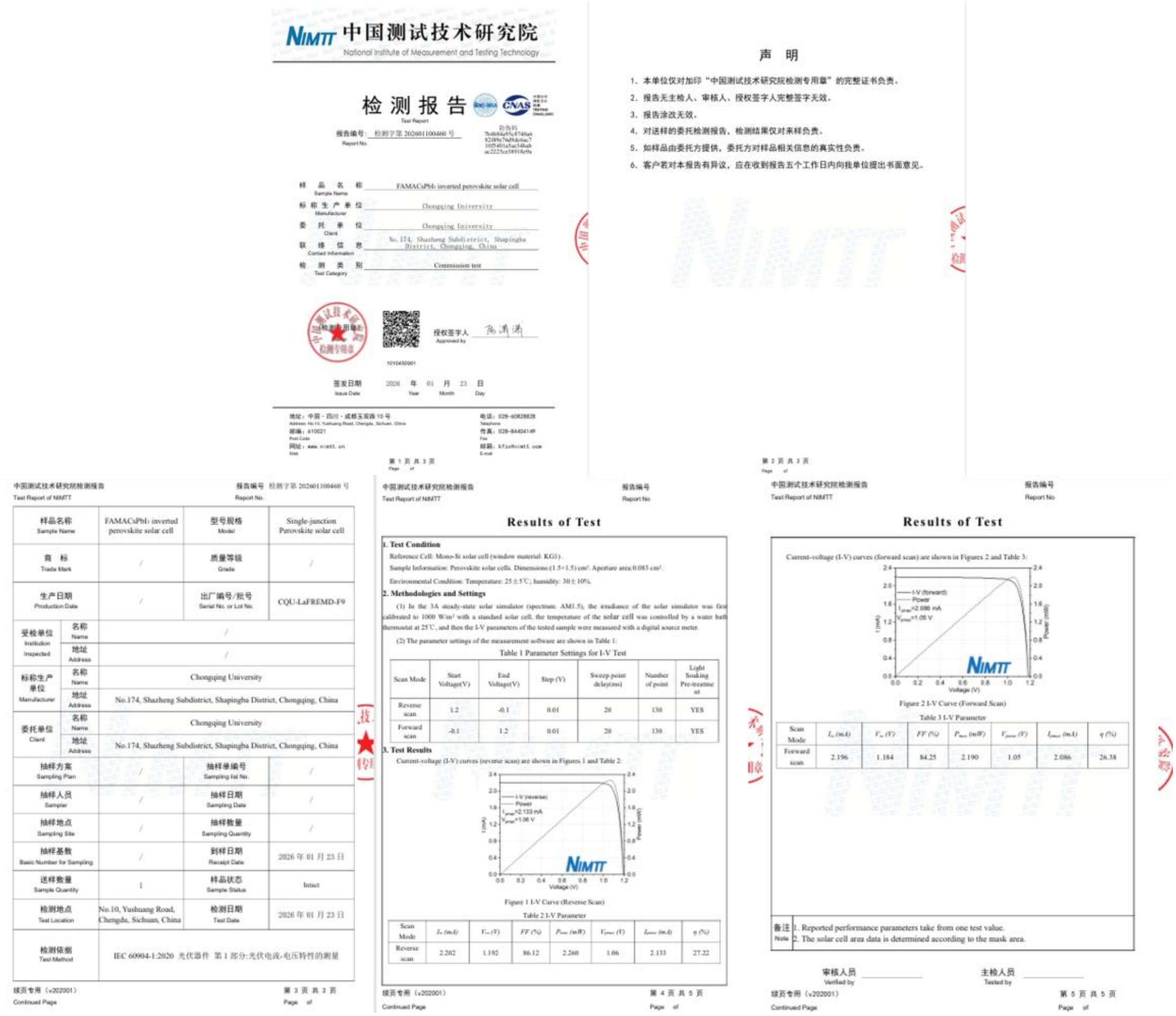

Fig. R8 Certified results from an accredited photovoltaic certification laboratory, National Institute of Measurement and Testing Technology (NIMTT).

Reviewer #2 (Remarks to the Author):

This manuscript addresses a critical challenge in perovskite solar cell (PSC) research for optimizing hole transport layers (HTLs) to mitigate aggregation, poor wettability, and weak interfacial interactions in self-assembled monolayers (SAMs) and presents a solution through rational molecular design, which demonstrates advancements in both device efficiency and stability. However, I need to reject the submission based on following adjustment:

1. Novelty of Material Design

From the standpoint of material design, the present work appears to assemble several previously reported SAM architectures, which diminishes the overall novelty. Moreover, concerning the authors' proposed novel molecular self-assembly model termed "hang-in-hand," reliance solely on elementary molecular dynamics simulations is insufficient to substantiate the physical existence of this configuration. And according to the figure 1m-o, it is hard to identify the "more ordered" structure of DMPA compared with the control SAM and BCPA. And for such kind of molecular packing on NiO_x could lead to passivation effect of PA group for control SAM and BCPA. More detailed characterizations should be provided to determine the packing model of SAM deposited on NiOx, as well as the thin film thickness, which plays vital role in device performance.

Our response: We thank the reviewer for this comprehensive and insightful comment regarding the novelty of the material design, the proposed molecular configuration, and the structural characterization of the SAM layers. We also appreciate our editor and reviewer for providing us an opportunity to response our reviewer's concern.

(a) On the novelty of molecular design. We agree that SAM-based hole-selective layers have been widely explored. But in this work, our focus is not merely on introducing a new SAM architecture, in contrary, we emphasis more on rational molecular design that integrates multiple functions into a single molecule. Specifically, DMPA is designed to (1) introduce methoxy-functionalized aromatic units for enhanced intermolecular interactions and interfacial affinity, (2) improve wettability toward polar

precursor solutions, and (3) optimize interfacial energetics for efficient charge extraction. We have revised the manuscript to clarify this design rationale and avoid overstating structural novelty as follow: "Molecular dynamics (MD) simulations visualized the top and side views of Control, BCPA, and DMPA on the NiO_x substrate (Fig. 1g-l). The results show that DMPA SAM effectively suppress its own aggregation and improve substrate coverage. This can be attributed to the optimal π-π interactions in DMPA, which enhance surface coverage, while the rotating phenyl ring introduces steric hindrance that effectively inhibits molecular stacking. In contrast, BCPA displays a thickness of approximately 1.6 nm, likely due to its greater tendency for molecular aggregation (Supplementary Fig. 7)."

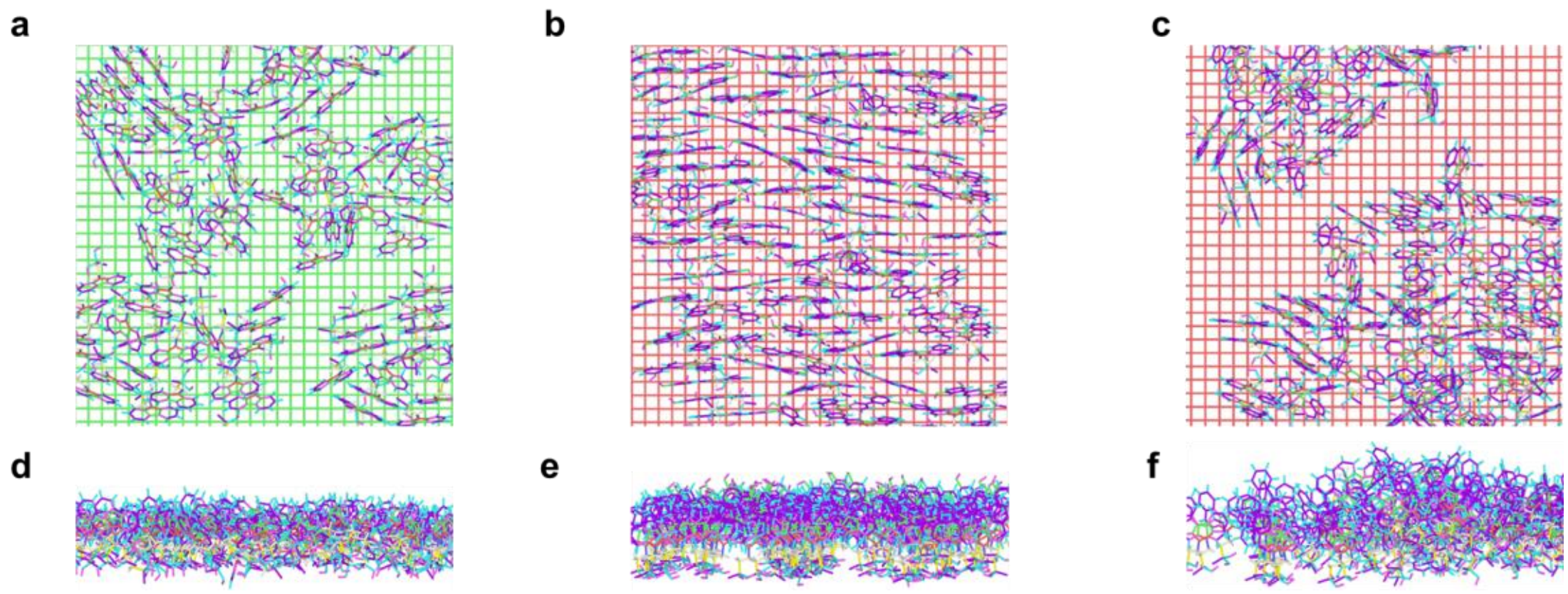

Fig. R9 **a - c** Top-view and **d - f** side-view molecular dynamics snapshots of the SAM configurations on NiO_x, illustrating differences in packing order, molecular orientation, and surface coverage.

(b) On the proposed "hand-in-hand" configuration. We fully agree that the current evidence is insufficient to experimentally validate a specific molecular packing model such as the "hand-in-hand" configuration. In response, we have removed all related descriptions from the revised manuscript to avoid overinterpretation. Instead, we adopt an evidence-based interpretation, attributing the improved performance to enhanced intermolecular interactions, optimized molecular packing, and improved surface coverage, consistent with previous reports (Fig. R10; *Nature* **2023**, *624*, 289; *Nature*

Photonics **2026**, *20*, 232–240; *Nature Communications* **2026**, *17*, 1472; *Nature Photonics* **2026**, *20*, 287-295).

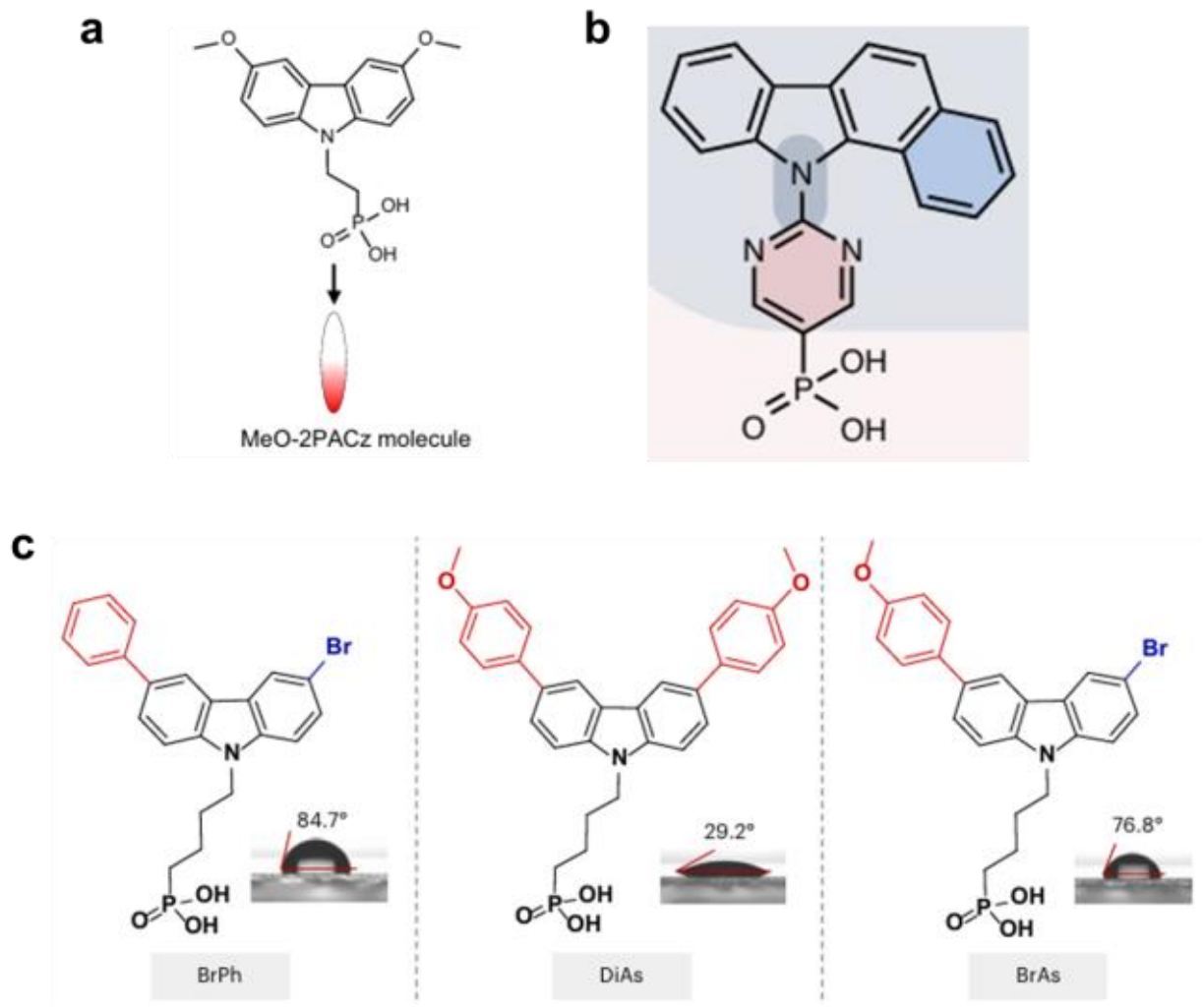

Fig. R10. Recent published work of SAMs (a) *Nature Communications* **2025**, *16*, 7245. (b) *Nature Photonics* **2026**, *20*, 232－240. (c) *Nature Photonics* **2026**, *20*, 287-295.

(c) On structural characterization of SAM packing. We acknowledge that directly resolving the precise molecular packing configuration of SAMs on NiO_x remains experimentally challenging. Instead of relying on a specific structural model, we provide indirect but consistent experimental evidence supporting improved SAM quality, including enhanced surface uniformity, improved wettability, and more reproducible device performance. These observations collectively indicate more uniform and effective surface coverage of the DMPA layer compared to the control SAM and BCPA.

(d) Film thickness. We have measured the thickness of the SAM layers and included the results in Fig. R11. The thickness is in the range of 0.8 ~ 1.6 nm, this further supports that the observed performance differences originate from molecular-level interfacial modification.

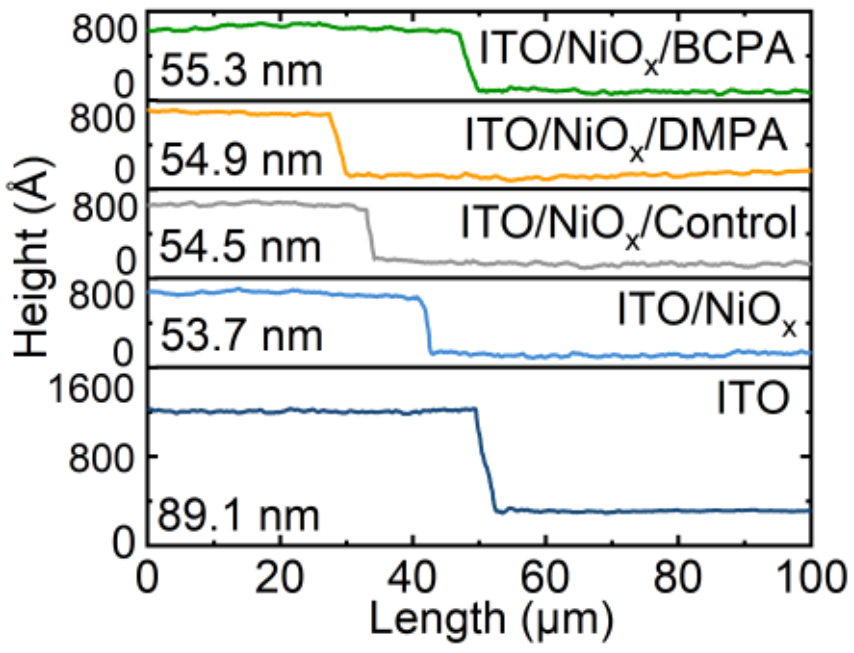

Fig. R11. The thickness of films.

Overall, we have revised the manuscript to (1) remove unsupported structural claims, (2) emphasize experimentally supported mechanisms, and (3) provide a more rigorous and balanced interpretation of the SAM structure–property relationships.

We appreciate the reviewer's comments, which have significantly improved the clarity and rigor of our work.

2. Efficiency Claims and Third-Party Validation

The power conversion efficiency reported herein ranks among the highest values achieved to date for perovskite photovoltaic devices employing SAM/NiOx hole-transport layers. However, for a manuscript submitted to a high-impact journal such as Nature Communications, efficiency values must be independently verified by an accredited third-party institution; in the absence of such certification, the reported performance data remain unconvincing.

Our response: We agree with the reviewer for this valuable suggestion. We have provided a third-party certification report (certified PCE: 27.22%) in Fig R12. The certification report has been updated in the supporting information.

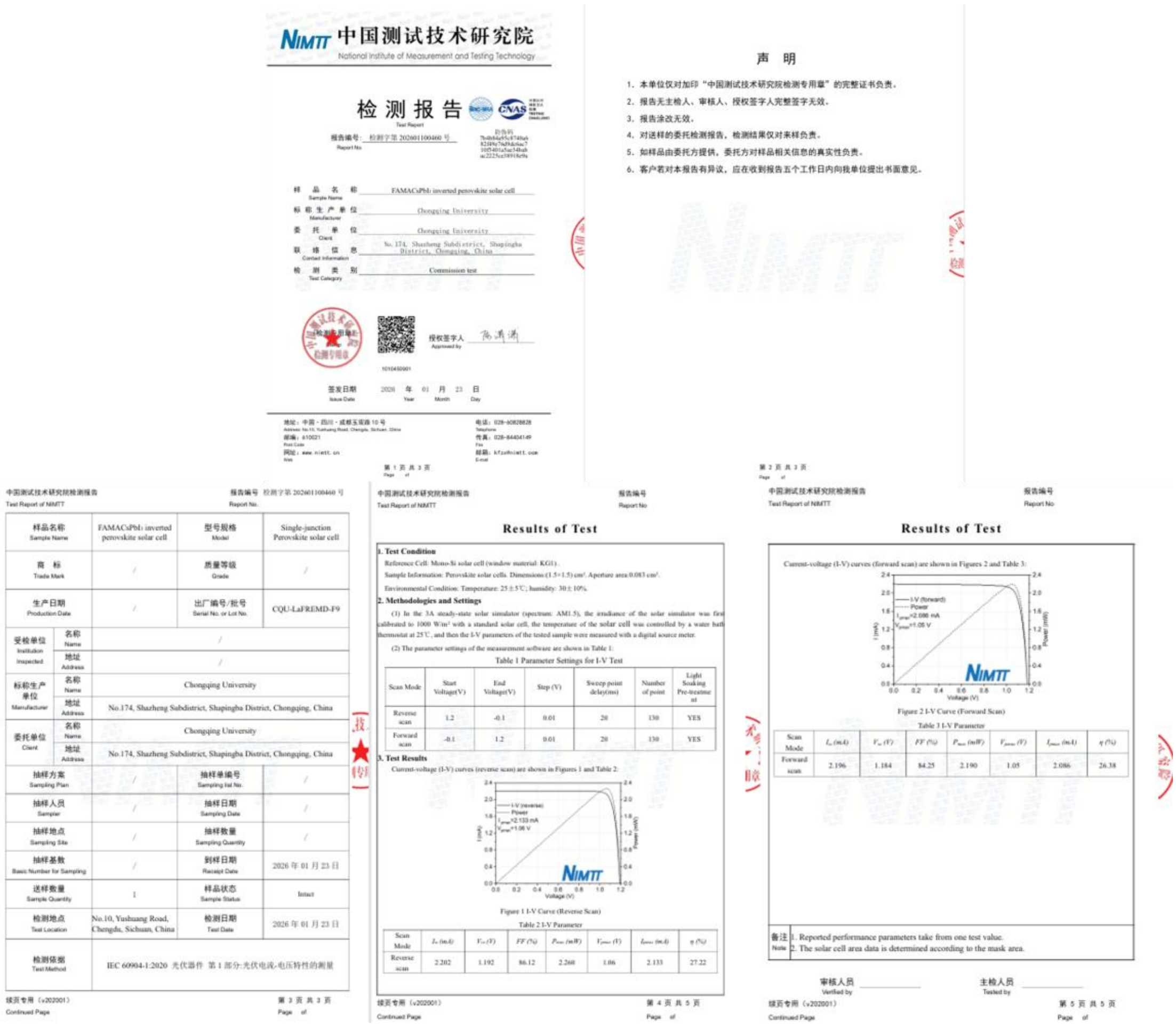

Fig. R12. The third-party certification report.

3. Consistency and Uniformity of Device Performance

The study lacks fundamental investigations into batch-to-batch consistency and spatial uniformity of device efficiency, essential benchmarks for evaluating reproducibility. Consequently, I harbor reservations regarding the reliability of the stated device performance metrics.

Our response: We sincerely thank the reviewer for this important comment regarding the reproducibility and uniformity of device performance.

To evaluate batch-to-batch consistency, we fabricated a total of 30 devices across six independent batches. As shown in Figure 5c, the devices exhibit a narrow distribution of PCE values with good reproducibility, demonstrating consistent device performance across different fabrication runs. Furthermore, to ensure consistency during fabrication, experimental conditions were carefully controlled, including

maintaining a stable glovebox environment (periodic purification, controlled atmosphere) and a constant temperature range of 20 - 23 °C.

These results collectively demonstrate the high reproducibility and uniformity of the device performance, supporting the reliability of the reported metrics.

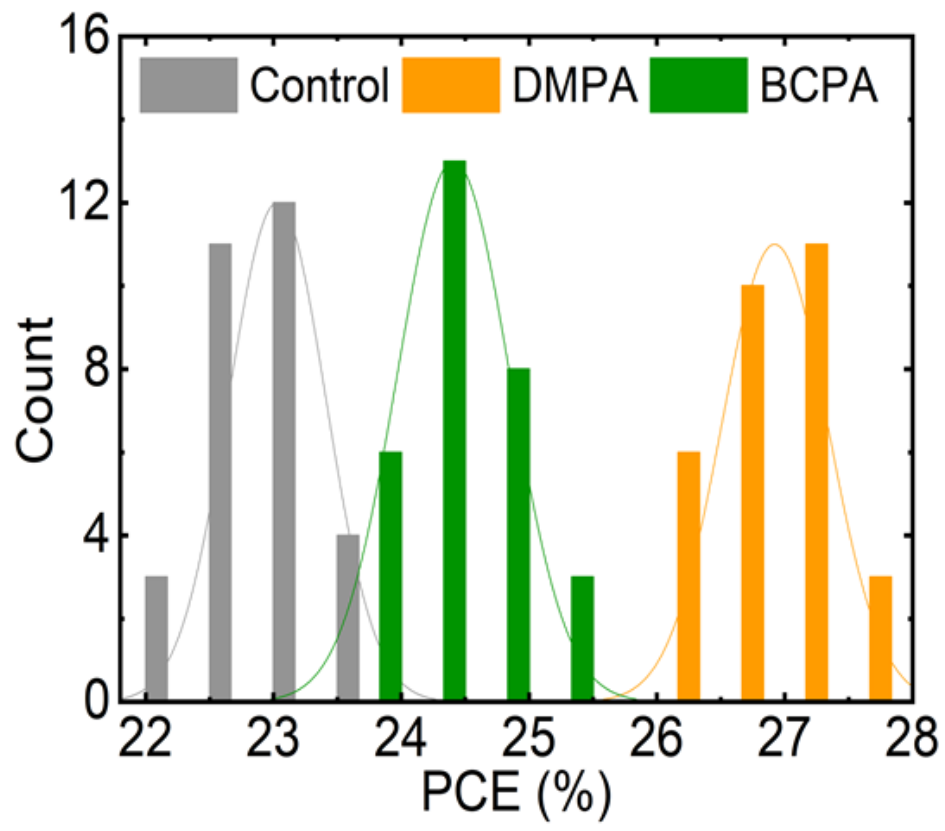

Fig. R13. The statistics of the PCE values for each type of the 30 PSCs.

4. Scalability to Large-Area Devices

Researchers active in perovskite photovoltaics are well aware that the field has advanced to large-area device and module fabrication. The omission of any demonstration of large-area devices based on this ostensibly high-performance material casts doubt on the scalability of its advantageous properties.

Our response: We sincerely thank the reviewer for this important comment regarding the scalability of our approach. We agree that demonstrating large-area devices or modules is an important step toward practical application. Due to current experimental limitations, we have not yet fabricated large-area devices. However, we would like to emphasize that the key contribution of this work lies in the molecular design of the SAM layer, which is inherently compatible with scalable fabrication processes. The DMPA modification is introduced via a solution-based deposition method, which can be extended to large-area coating techniques such as blade coating or slot-die coating. Furthermore, as demonstrated in Figure 5c, the devices exhibit excellent reproducibility across multiple batches, indicating stable and controllable film formation. These features are essential prerequisites for scaling up to large-area devices.

Therefore, while large-area devices are beyond the scope of the current study, the

demonstrated uniformity, reproducibility, and process compatibility strongly support the scalability potential of this approach.

5. Stability Testing Under Thermal Stress

With respect to stability assessment, a device employing only BCP as the electron-blocking layer is unlikely to maintain stable operation under prolonged thermal stress at 65 °C. Therefore, the presented stability data warrant careful scrutiny and may not be fully credible.

Our response: We thank the reviewer for this important comment regarding the thermal stability of the device. We would like to clarify that the maximum power point (MPP) stability measurements were conducted on devices employing a C_{60}/SnO_2 electron transport structure, without the use of BCP. We agree with the reviewer that BCP can exhibit limited thermal stability under prolonged thermal stress, and therefore it was intentionally excluded from the device architecture used for stability evaluation.

We apologize for the insufficient description in the original manuscript, which may have caused confusion regarding the device structure used for the stability tests. In the revised version, we have clarified the exact device configuration and testing conditions for the MPP measurements and displayed as follow: "The stability test following the ISOS-L-2 protocol was conducted under continuous heating at ~65 °C, AM1.5G illumination (100 mW cm^{-2}, white LED) and open-circuit condition. The device configuration is ITO/NiO_x/SAM/PVK/PDI/C_{60}/SnO_2/Ag. The PCEs of the devices were tracked over time, and the J–V measurements were performed after cooling the devices down to room temperature. The MPPT of the devices was conducted under AM1.5G illumination at ~65 °C. The voltage at the maximum power point was automatically adjusted and applied, and the power output of the devices was tracked over time."

These details have been added to the manuscript to ensure accuracy and transparency.

6. High-Resolution Mass Spectrometry Reporting

The high-resolution mass spectrometry entry stating "HRMS-ESI m/z Calcd for $C_{18}H_{16}NO_3P$ $[M+H]^+$: 326.09, found 326.09" contains a serious error and does not reflect acceptable reporting standards.

Our response: We thank the reviewer for pointing out this important issue regarding the HRMS reporting. We acknowledge that the original HRMS data were not presented with sufficient precision. In the revised manuscript, we have corrected the values and provided accurate masses with appropriate significant figures and mass deviations (in ppm).

The following revisions have been made in the manuscript:

"HRMS (ESI^+) m/z calcd for $C_{26}H_{24}NO_5P$ $[M+H]^+$ 462.1465, found 462.1466 ($\Delta = 0.22$ ppm)." and "HRMS (ESI^+) m/z calcd for $C_{18}H_{16}NO_3P$ $[M+H]^+$ 326.0941, found 326.0942 ($\Delta = 0.31$ ppm)."

Thank you again for your constructive feedback, which has significantly strengthened our study.

7. Anomalies in J–V Scan Directions

Analysis of the reported J–V characteristics reveals an inconsistency: for the control and BCPA devices, all reverse-scan parameters (V_{OC}, J_{SC}, and fill factor) exceed their forward-scan counterparts, whereas for the DMPA device the reverse-scan V_{OC} and J_{SC} are lower than in the forward scan. This discrepancy merits explanation, as it suggests potential measurement artefacts or inconsistencies in device behavior.

Our response: We thank the reviewers for the insightful comment. We believe the observed inconsistencies might come from two aspects: (i) The incorporation of DMPA reduced the presence of voids, thereby suppressing non-radiative recombination and consequently improving the device performance parameters. (ii) Device performance is routinely measured multiple times, and the sequence of scan direction in perovskite solar cells can influence the measured performance. In the revised manuscript, we have taken caution to the sequence of scan directions and updated the relevant data in the manuscript and displayed as follow: "For the champion devices, *J-V* parameters under reverse and forward scans are summarized as follows: for the DMPA device, V_{OC} =

1.195 V and 1.193 V, J_{SC} = 26.58 mA cm^{-2} and 26.53 mA cm^{-2}, *FF* = 86.89% and 84.86%, PCE = 27.59% and 26.86%, respectively". We thank the reviewer again for helping to improve the quality of our paper.

8. Some minor error

In page 8, the mention of "DCPA" should be wrong.

In Page 10, The description of "The surface molecular density of NiO_x/DMPA (6.8 × 1013 molecules cm^{-2}) was higher than those of NiO_x/Control SAM (5.0 × 1013 molecules cm^{-2}) and NiO_x/BCPA (6.8× 1013 molecules cm^{-2})" is wrong.

Our response: We thank the reviewer for carefully identifying these errors. The corresponding revisions have been made in the main text and displayed as follow: "DCPA" changed to "DMPA"; and numbers are corrected "The surface molecular density of NiO_x/DMPA (6.8 × 10^{13} molecules cm^{-2}) was higher than those of NiO_x/Control SAM (4.5 × 10^{13} molecules cm^{-2}) and NiO_x/BCPA (5.0 × 10^{13} molecules cm^{-2})"

Reviewer #3 (Remarks to the Author):

This study reports a strategy to enhance the efficiency and stability of inverted perovskite solar cells by designing a DMPA SAM molecule. The authors propose that DMPA adopts a unique "hand-in-hand" molecular configuration, leading to improved interfacial contact, reduced residual stress, and a high PCE of 27.59%. While the findings are interesting and the performance are impressive, the manuscript requires solid experimental evidence to substantiate the proposed molecular configuration. Additionally, independent certification of the efficiency and further clarification on the stress relief mechanism are needed to support the conclusions.

1. A primary concern is that the authors claim that DMPA adopts a "hand-in-hand" configuration while the Control and BCPA adopt a vertical stacking mode. While the simulation is supportive, it is not experimental evidence. The authors should provide direct experimental evidence of this specific configuration. Without experimental corroboration, the "hand-in-hand" mechanism remains a theoretical hypothesis.

Our response: We sincerely thank the reviewer for this insightful and important comment regarding the proposed “hand-in-hand” molecular configuration. We fully agree that the current evidence, primarily based on theoretical simulations, is insufficient to unambiguously establish a specific molecular configuration such as the “hand-in-hand” arrangement. In light of this, we have carefully re-evaluated our interpretation and removed all related descriptions from the revised manuscript to avoid overinterpretation.

Based on our simulations, together with experimental observations and supported by literature reports (*Nature* **2023**, *624*, 289; *Nature Photonics* **2026**, *20*, 232–240; *Nature Communications* **2026**, *17*, 1472; *Nature Photonics* **2026**, *20*, 287-295), we attribute the improved performance of DMPA to enhanced intermolecular interactions, optimized molecular packing, and improved surface coverage. Importantly, the conclusions of this work do not rely on the existence of a particular molecular arrangement, but are instead supported by consistent experimental observations,

including improved film morphology, reduced defect density, enhanced charge extraction, and reduced residual stress.

We have revised the manuscript accordingly to present a more cautious and evidence-supported interpretation.

The corresponding descriptions are provided as follow: "Molecular dynamics (MD) simulations visualized the top and side views of Control, BCPA, and DMPA on the NiO_x substrate (Fig. 1g-l). The results show that DMPA SAM effectively suppress its own aggregation and improve substrate coverage. This can be attributed to the optimal π-π interactions in DMPA, which enhance surface coverage, while the rotating phenyl ring introduces steric hindrance that effectively inhibits molecular stacking. In contrast, BCPA displays a thickness of approximately 1.6 nm, likely due to its greater tendency for molecular aggregation (Supplementary Fig. 7).

Additionally, we utilized DFT to determine E_{ad} of these SAMs on PVK. Notably, compared to E_{ad} of control (-0.28 eV) and BCPA (-0.32 eV), the E_{ad} of DMPA (-0.39 eV) with perovskite is the most negative (Supplementary Fig. 8a), signifying that DMPA possesses the strongest affinity among the three SAMs."

We appreciate the reviewer's comment, which help us to improve the rigor and clarity of our work.

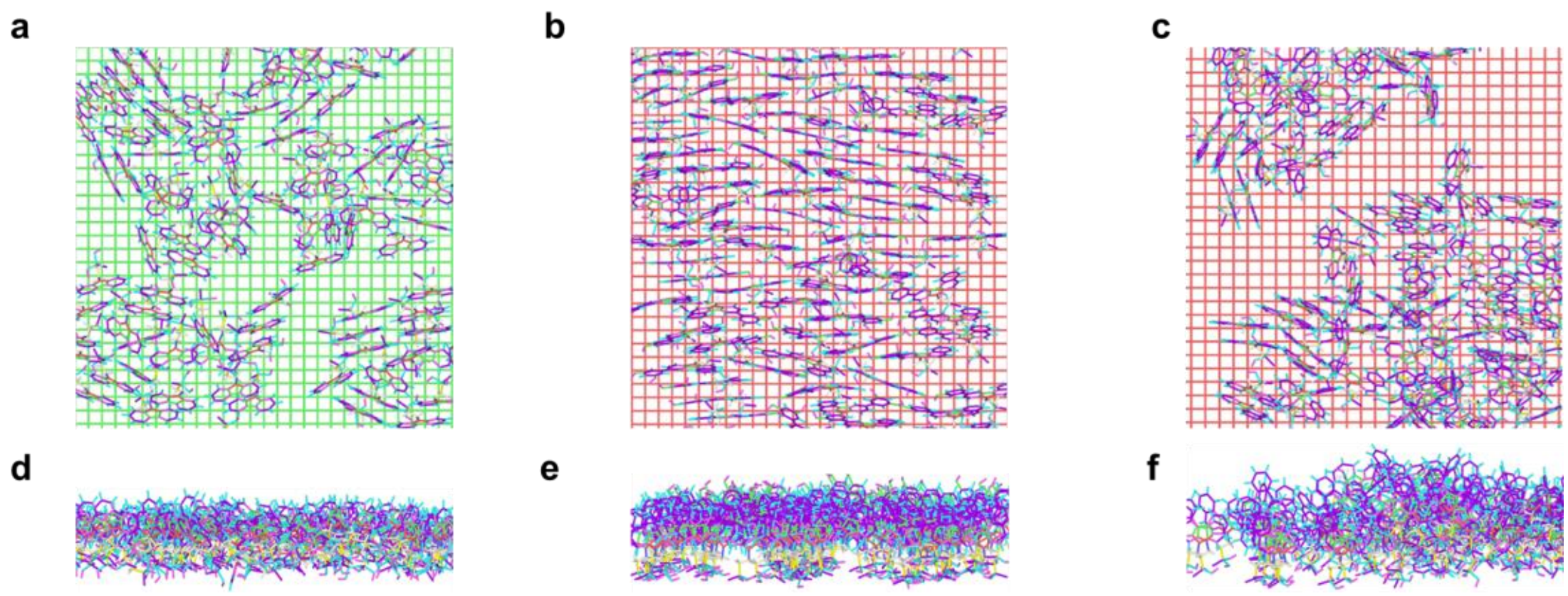

Fig. R14 **a - c** Top-view and **d - e** side-view molecular dynamics snapshots of the SAM configurations on NiO_x, illustrating differences in packing order, molecular orientation, and surface coverage.

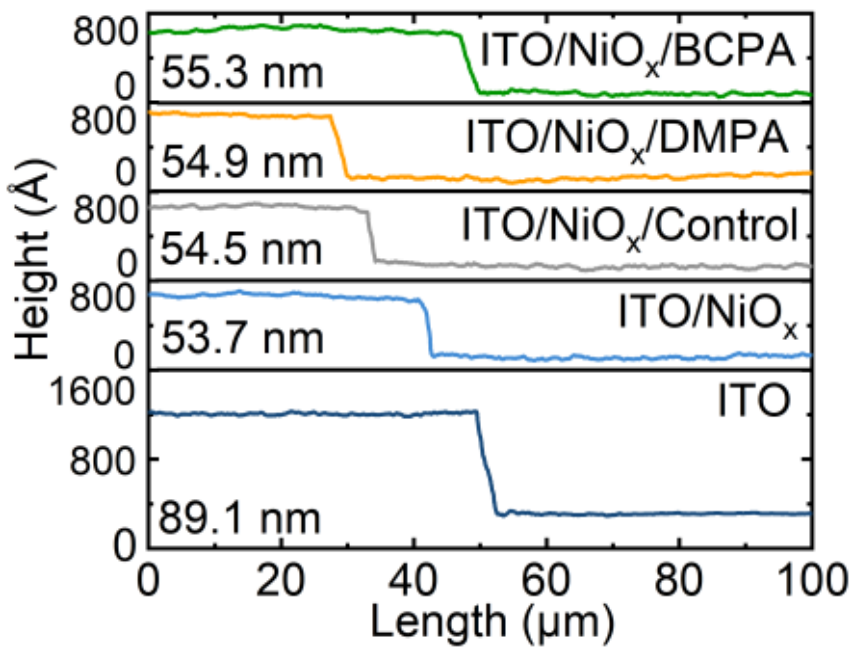

Fig. R15. The thickness of films.

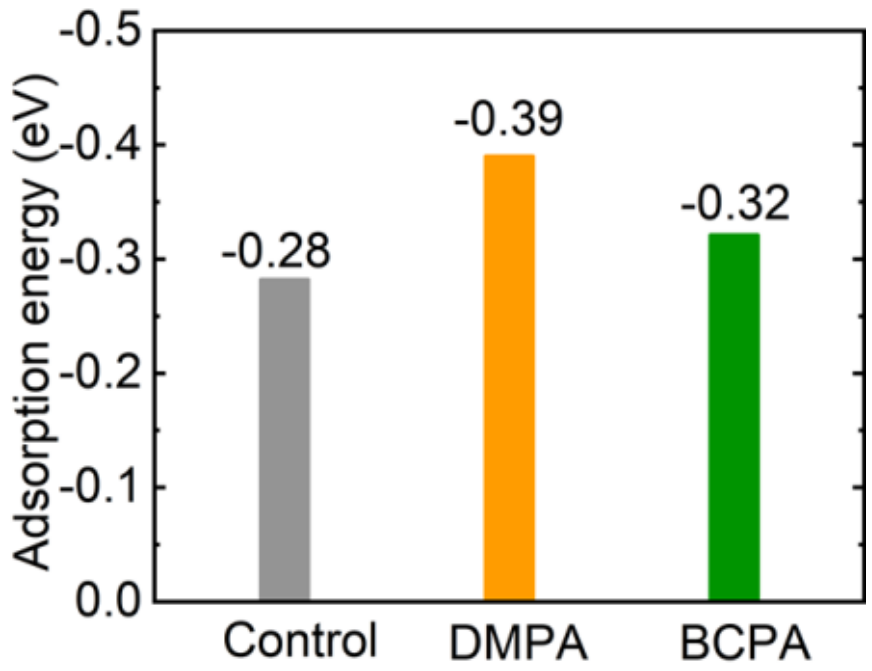

Fig. R16 Adsorption energy (E_{ad}) of different SAM molecules on the perovskite surface, obtained from DFT calculations.

2. A lower initial GSB amplitude could imply less absorption or immediate quenching, but "faster transfer" is usually derived from the decay kinetics (how fast the signal fades), not just the intensity. A normalized decay kinetic plot of the GSB peak should be provided to compare the hole extraction rates.

Our response: We thank the reviewers for their insightful comments regarding the interpretation of the transient absorption results. We agree that the ground-state bleaching (GSB) signal primarily reflects state filling and that its initial amplitude may be influenced by factors such as absorption differences or ultrafast processes. Therefore, the initial GSB intensity cannot be directly used to infer charge transfer dynamics.

To address this point, we have extracted and provided normalized decay kinetics of the GSB signal at ~803 nm for all samples (Fig. R18). The normalized decay curves clearly show that the DMPA-treated sample exhibits a faster decay compared to the

control and BCPA samples. In device-relevant NiO_x/SAM/perovskite, the recovery of the GSB signal is governed by both carrier recombination and interfacial charge extraction. Therefore, the faster decay observed for the DMPA sample is attributed to more efficient hole extraction at the NiO_x/SAM/perovskite interface.

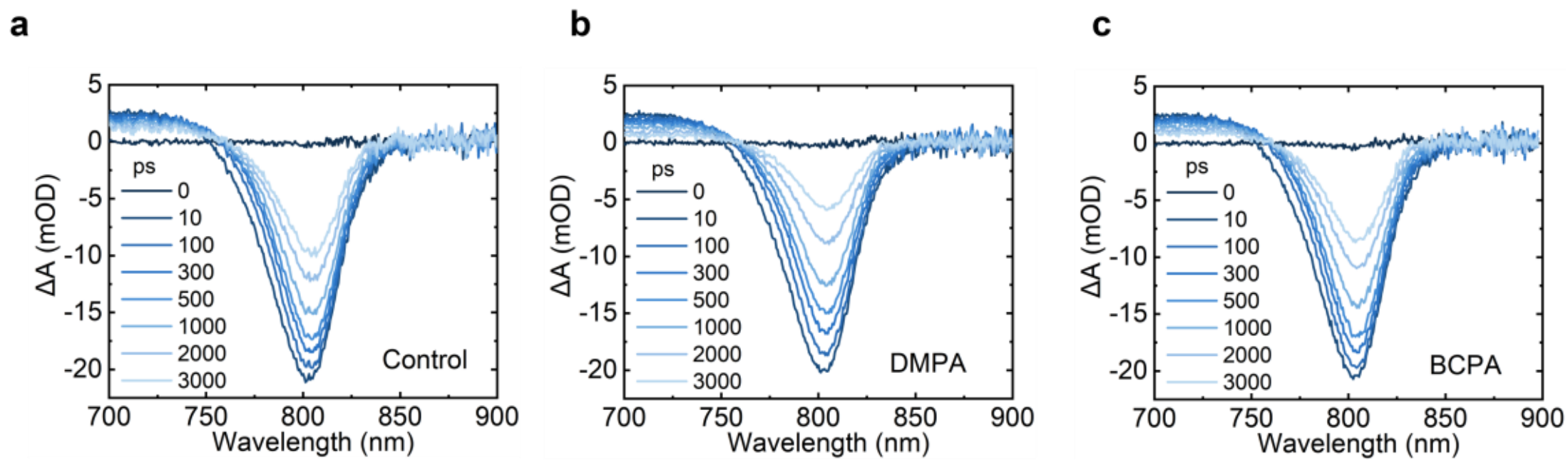

Fig. R17. Femtosecond transient absorption spectra at a selected pump − probe delay time of (a) NiO_x/control/perovskite films, (b) NiO_x/DMPA/perovskite films and (c) NiOx/BCPA/perovskite films. ΔA, difference in optical density between the excited state and the ground state of a sample.

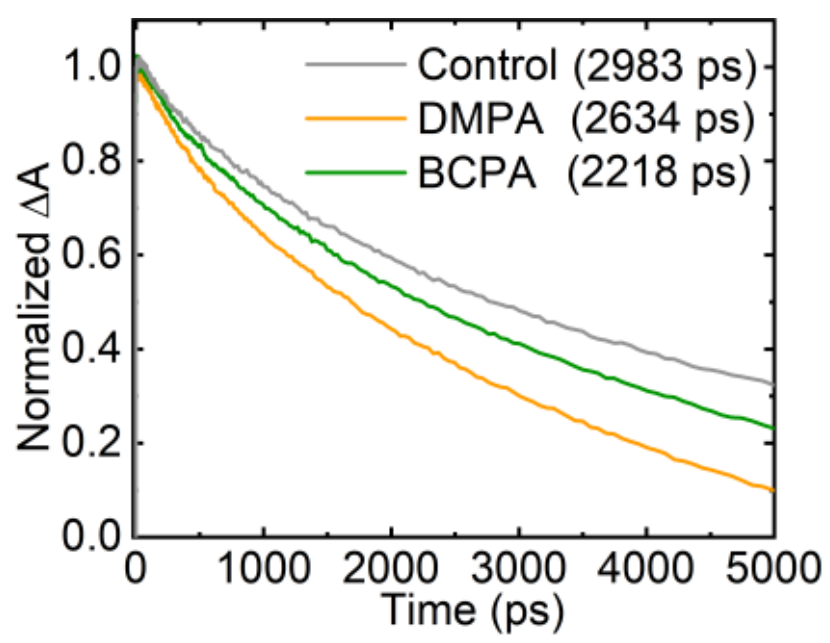

Fig. R18. Corresponding GSB decay at 803 nm of the different SAM treatment.

The corresponding descriptions are provided below and have been updated in the main text. "We further employed femtosecond transient absorption (TA) spectroscopy to investigate the interfacial charge dynamics in NiO_x/SAM/perovskite stacks (Fig. 4j–l). All samples exhibit a characteristic ground-state bleaching (GSB) signal centered at ~803 nm, corresponding to state filling of the perovskite absorber. It should be noted that the GSB signal primarily reflects the population of excited states and does not directly represent free-carrier dynamics such as recombination or transport. In device-relevant structures, the recovery of the GSB signal is governed by a combination of

carrier recombination and interfacial charge extraction processes.

The DMPA sample exhibits a faster GSB recovery compared to the control and BCPA samples (Supplementary Fig. 20). This behavior suggests more efficient interfacial hole extraction at the NiO_x/SAM/perovskite interface in the presence of DMPA."

We appreciate the reviewer's comments, which have helped us refine the interpretation and improve the rigor of the analysis.

3. The GIXRD data show a significant reduction in residual stress for DMPA-based films. The mechanism needs more explanation. How does a monolayer at the bottom interface relieve stress throughout the bulk of a thick perovskite film? Expanding on the relationship between the "hand-in-hand" assembly and the relaxation of lattice strain would strengthen the quality and impact of this study.

Our response: We thank the reviewer for this insightful comment regarding the mechanism by which the interfacial SAM influences the residual stress in the perovskite film. We agree that a monolayer at the buried interface cannot directly relax stress throughout the bulk film via a purely local effect. Instead, its influence is mediated through the regulation of crystallization dynamics and microstructure evolution during film formation.

Specifically, the DMPA layer modifies the nucleation and growth process of the perovskite. As revealed by in-situ GIWAXS measurements (Fig. R19), the DMPA-treated films exhibit a prolonged solvent-phase intermediate, indicating retarded crystallization kinetics. Such delayed crystallization typically leads to reduced nucleation density and extended crystal growth time, favoring the formation of larger and more ordered grains. This improved microstructure reduces grain boundary density and alleviates lattice mismatch between adjacent domains, which are major sources of residual stress in polycrystalline perovskite films. Therefore, the reduction in residual stress originates from the bulk microstructural evolution induced by interfacial regulation, rather than a direct mechanical effect of the SAM layer.

In addition, the flexible molecular structure of DMPA, featuring a rotatable

phenyl group, can accommodate local interfacial strain during the early stages of crystal formation, consistent with previous reports on interfacial molecular design (*Angew. Chem. Int. Ed.* **2025**,*138*, e21774) (Fig. R20).

We have revised the manuscript to clarify this mechanism and to avoid overinterpretation of direct stress relaxation by the SAM layer.

The corresponding descriptions are provided as folllow: "To uncover the underlying mechanism for crystal growth, we further conducted in-situ GIWAXS measurement under the programmed spin-coat and annealing procedure. The corresponding false-color maps of the control, DMPA, and BCPA of the diffraction intensity versus scattering vector q (in units per angstrom) and time (in seconds) are shown in Supplementary Fig. 16a-c. At the moment of antisolvent dripping, a solvent phase emerged. Subsequently, during the annealing process, characteristic diffraction peaks were observed, at q = 1.00, 1.42, and 1.73 $Å^{-1}$, corresponding to the (100), (110), and (111) facets of the perovskite, respectively. Compared to the Control and BCPA, DMPA exhibits a prolonged duration of the solvent phase, suggesting that the SAM molecule modulates perovskite growth. In other words, DMPA retards the crystallization kinetics of the perovskite, which can enhance perovskite crystallization and growth."

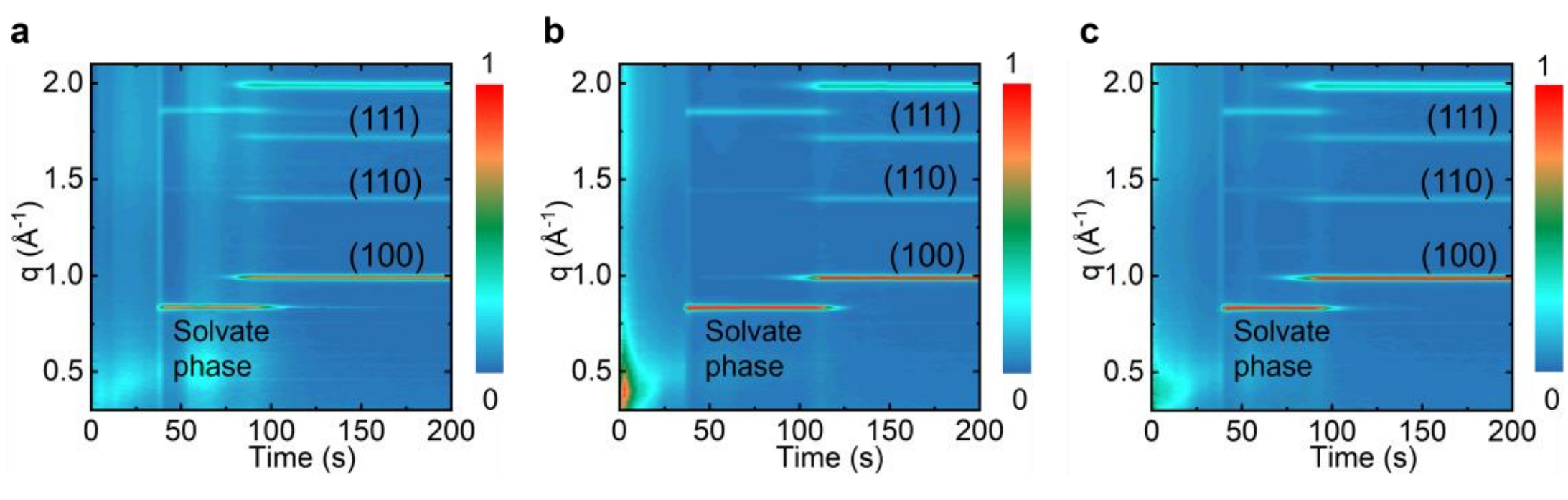

Fig. R19. False-color maps versus q (in units per angstrom) and time (in seconds) of the (a) control and (b) DMPA (c) BCPA PVK films in-situ GIWAXS tests.

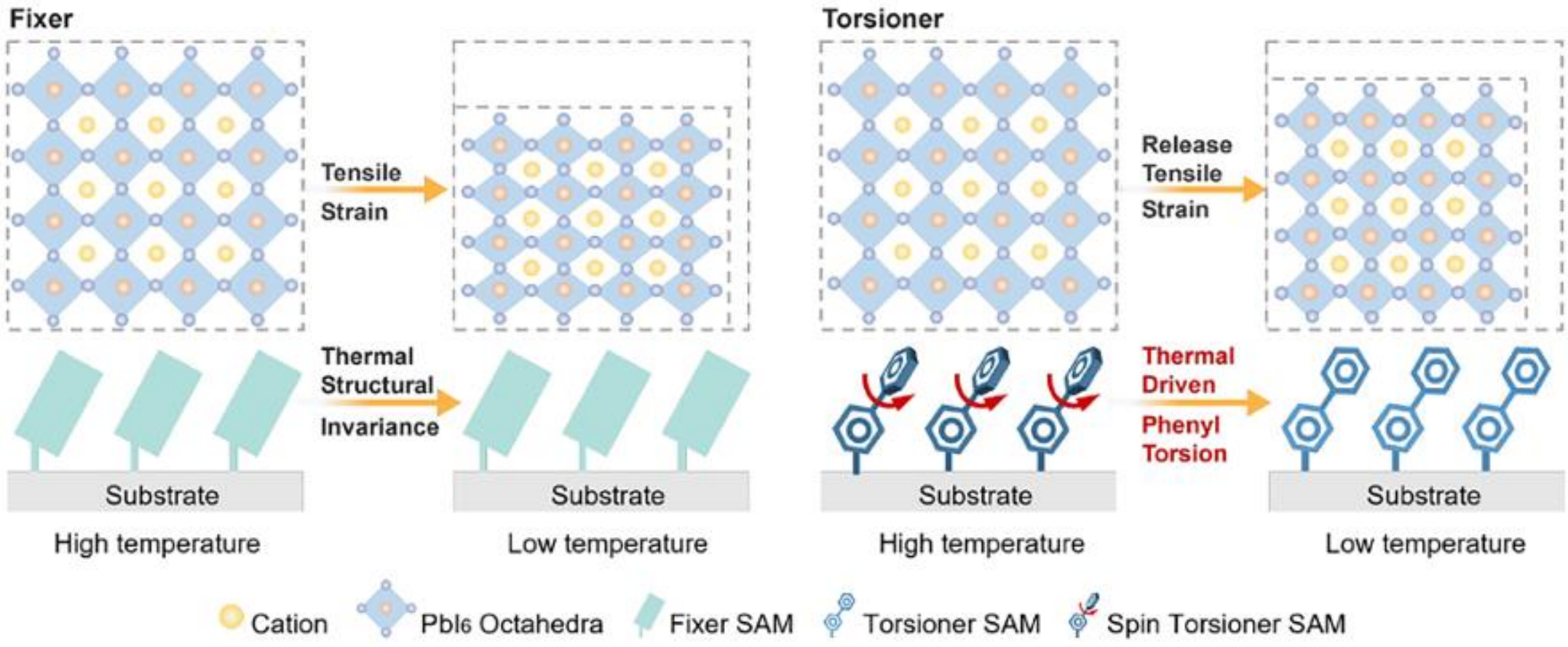

Figure R20. Reversible, thermal driven phenyl torsion in Torsioner SAM. Proposed mechanism for strain relief in perovskite films via Torsioner SAM.

4. The authors report a very high PCE of 27.59%. For efficiency claims exceeding 27.5% in a high-impact journal, independent certification from an accredited testing laboratory is standard practice and highly recommended.

Our response: We agree with the reviewer for this valuable suggestion. We have provided a third-party certification report (certified PCE: 27.22%) in Fig R21. The certification report has been updated in the supporting information.

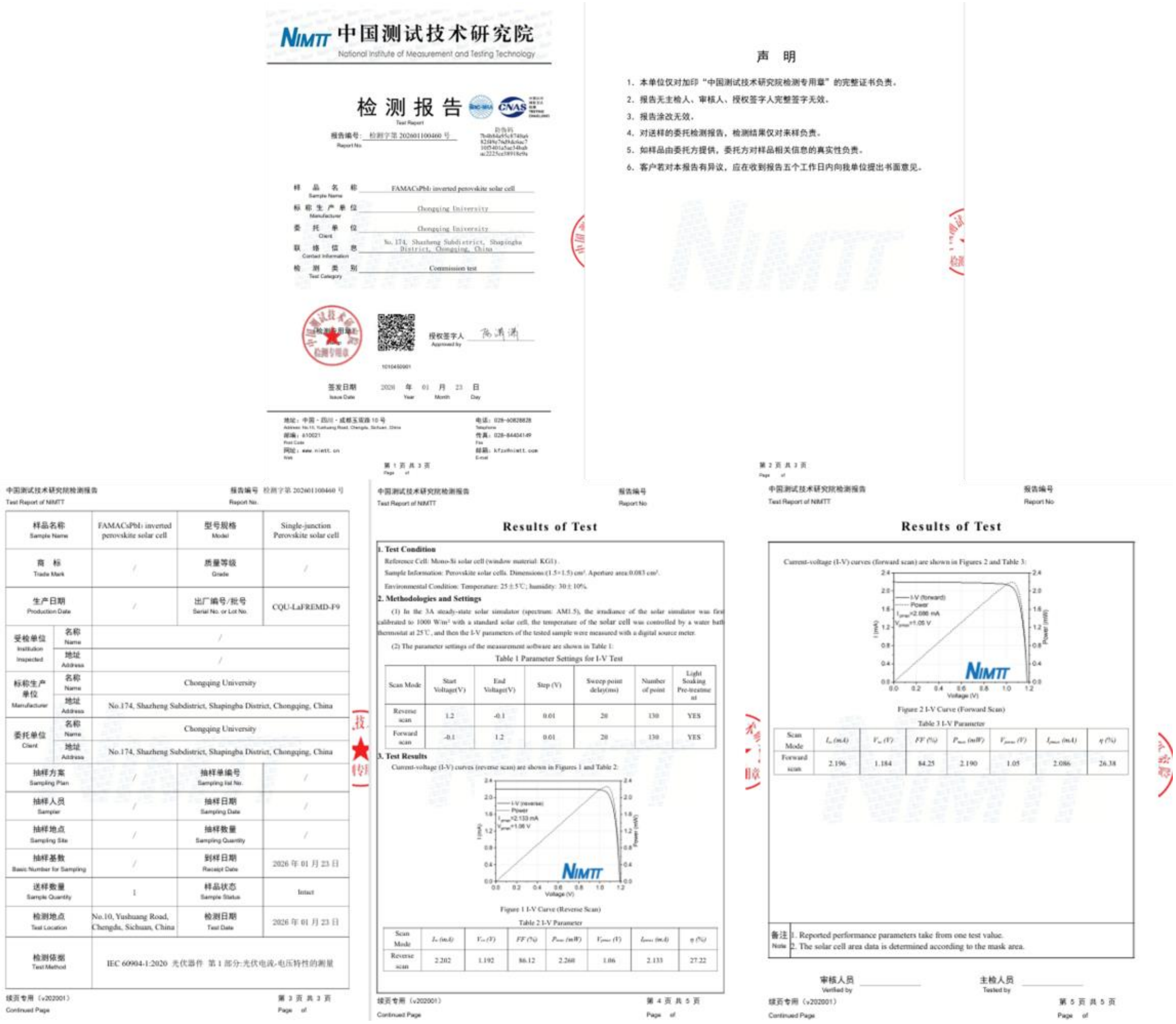

Scan Mode	I_{sc} (mA)	V_{oc} (V)	FF (%)	P_{max} (mW)	V_{pmax} (V)	I_{pmax} (mA)	η (%)
Reverse scan	2.202	1.192	86.12	2.260	1.06	2.133	27.22

Scan Mode	I_{sc} (mA)	V_{oc} (V)	FF (%)	P_{max} (mW)	V_{pmax} (V)	I_{pmax} (mA)	η (%)
Forward scan	2.196	1.184	84.25	2.190	1.05	2.086	26.38

Fig. R21. The third-party certification report.

5. Please provide SEM images with a larger field of view (e.g., 10 μm × 10 μm) to better evaluate the overall uniformity and quality of the perovskite films prepared on different SAMs.

Our response: We thank the reviewer for this valuable suggestion. In response, we have characterized the perovskite films using scanning electron microscopy (SEM) with a larger field of view (10 μm × 10 μm) to better evaluate the film uniformity and morphology across different SAMs (Fig. R22). The SEM image has been updated in the main text (Fig. 3d - f).

These images clearly show that the DMPA exhibit a more uniform and compact morphology with reduced pinholes and improved grain connectivity compared to the control and BCPA. This provides more direct evidence of enhanced film quality.

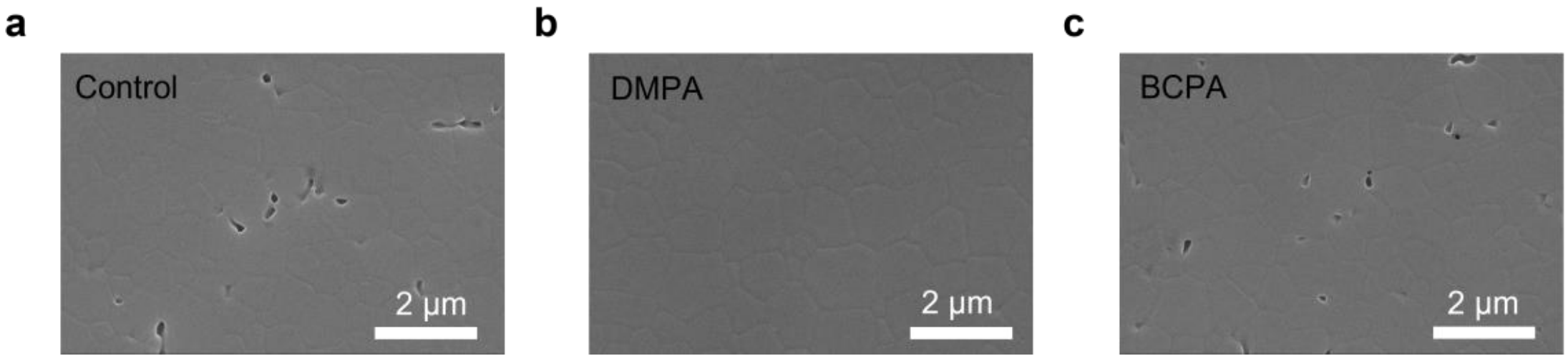

Fig. R22. SEM images of the bottom surface of perovskite deposited on different SAMs.

6. What is the specific mechanism responsible for the noticeable EQE enhancement in the 400–650 nm range for the DMPA-based device?

Our response: We thank the reviewer for this insightful question regarding the origin of the EQE enhancement in the 400 - 650 nm range. We attribute the improved EQE primarily to enhanced charge collection efficiency rather than optical absorption (the UV-vis absorption spectra Supplementary Fig. 19). Specifically, the following factors contribute to the observed enhancement:

(a) Improved interfacial quality and reduced recombination. The introduction of DMPA effectively passivates defects at the buried interface and suppresses void formation, leading to reduced non-radiative recombination. This improves the internal quantum efficiency (IQE), which is the dominant factor governing EQE in this spectral range.

(b) Enhanced charge extraction. As evidenced by transient absorption (TA) and PL measurements, the DMPA-based devices exhibit more efficient interfacial charge extraction. This reduces carrier loss before collection, particularly for photogenerated carriers created near the front interface.

(c) Energy-level alignment. DMPA improves the interfacial energetics between NiO_x and the perovskite, facilitating more efficient hole transfer and reducing energy loss during charge extraction (such as: Recent published work of SAMs (a) *Nature Communications* **2025**, *16*, 7245. (b) *Nature Photonics* **2026**, *20*, 287-295.).

Therefore, the EQE enhancement in the 400 - 650 nm region is mainly attributed to improved internal quantum efficiency resulting from defect passivation and more efficient charge extraction.

7. Please specify the initial PCE values for each device used in the MPPT tests to provide a clear baseline for stability comparison.

Our response: We sincerely appreciate the reviewer's insightful and valuable suggestion. The initial power conversion efficiency (PCE) values have been annotated in Figure 5h: 23.3% for the control device, 24.9% for the BCPA-based device, and 26.8% for the DMPA-based device.

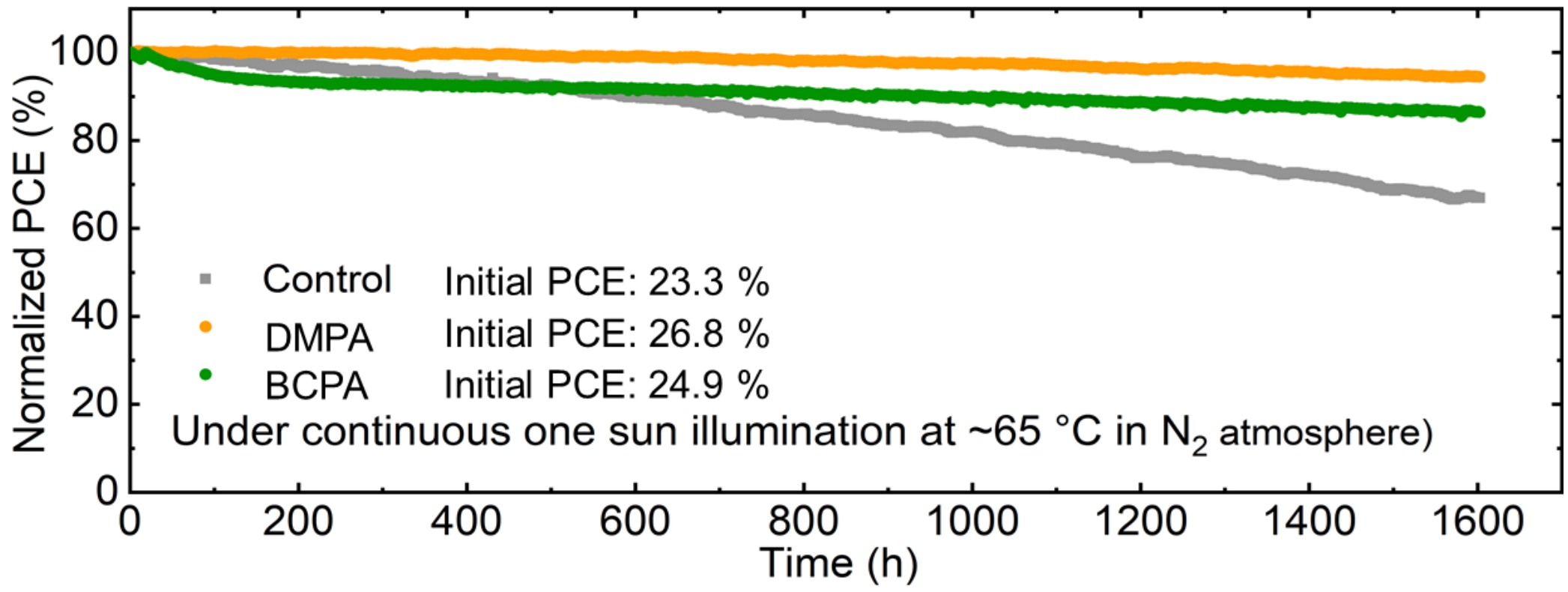

Fig. R23. MPP tracking under N_2 and simulated 1 sun AM 1.5 G illumination for devices (reaching an operating temperature of 65 ± 10 °C).

8. Additionally, please ensure that the x- or y-axis span of figs. 2g-2i are identical across all samples to enable a fair visual comparison of the potential distributions.

Our response: We appreciate the reviewer's insightful suggestion. We have revised Figures 2g - 2i by standardizing the x- and y-axis ranges across all samples. This ensures a consistent scale for the potential distributions and enables a direct and fair visual comparison. The updated figures have been incorporated into the revised manuscript.

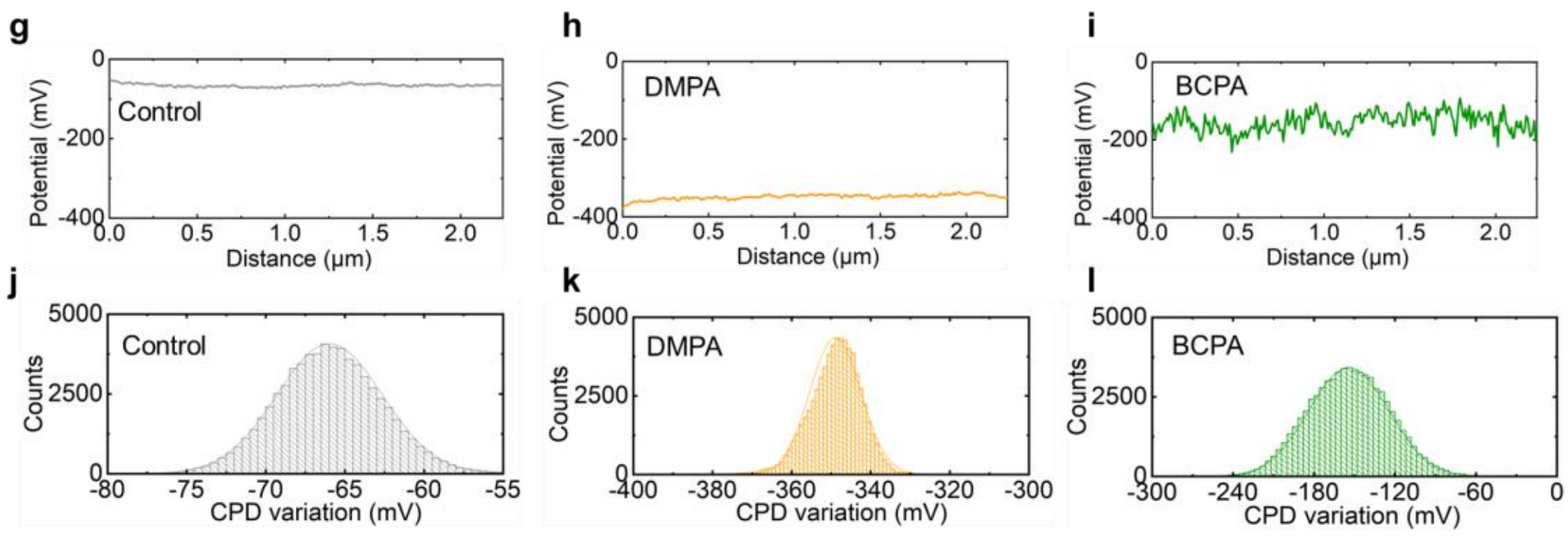

Fig. R24. The CPD line scan extracted from the dotted line position for **g** NiOx/ Control

SAM, **h** NiOx/DMPA and **i** NiOx/BCPA. The CPD distribution histogram of **j** NiOx/Control SAM, **k** NiOx/DMPA and **l** NiOx/BCPA.
